# Supplementary material for: Quantitative Proteomics Analysis of Lytic KSHV Infection in Human Endothelial Cells Reveals Targets of Viral Immune Modulation
Source: Cell Rep. 2020 Oct 13;33(2):108249. doi: 10.1016/j.celrep.2020.108249 (PMC7567700; doi:10.1016/j.celrep.2020.108249)
Supplement: Document S1. Figures S1–S7 [file mmc1.pdf]

Cell Reports, Volume *33*

Supplemental Information

Quantitative Proteomics Analysis of Lytic KSHV Infection in Human Endothelial Cells Reveals Targets of Viral Immune Modulation

Ildar Gabaev, James C. Williamson, Thomas W.M. Crozier, Thomas F. Schulz, and Paul J. Lehner

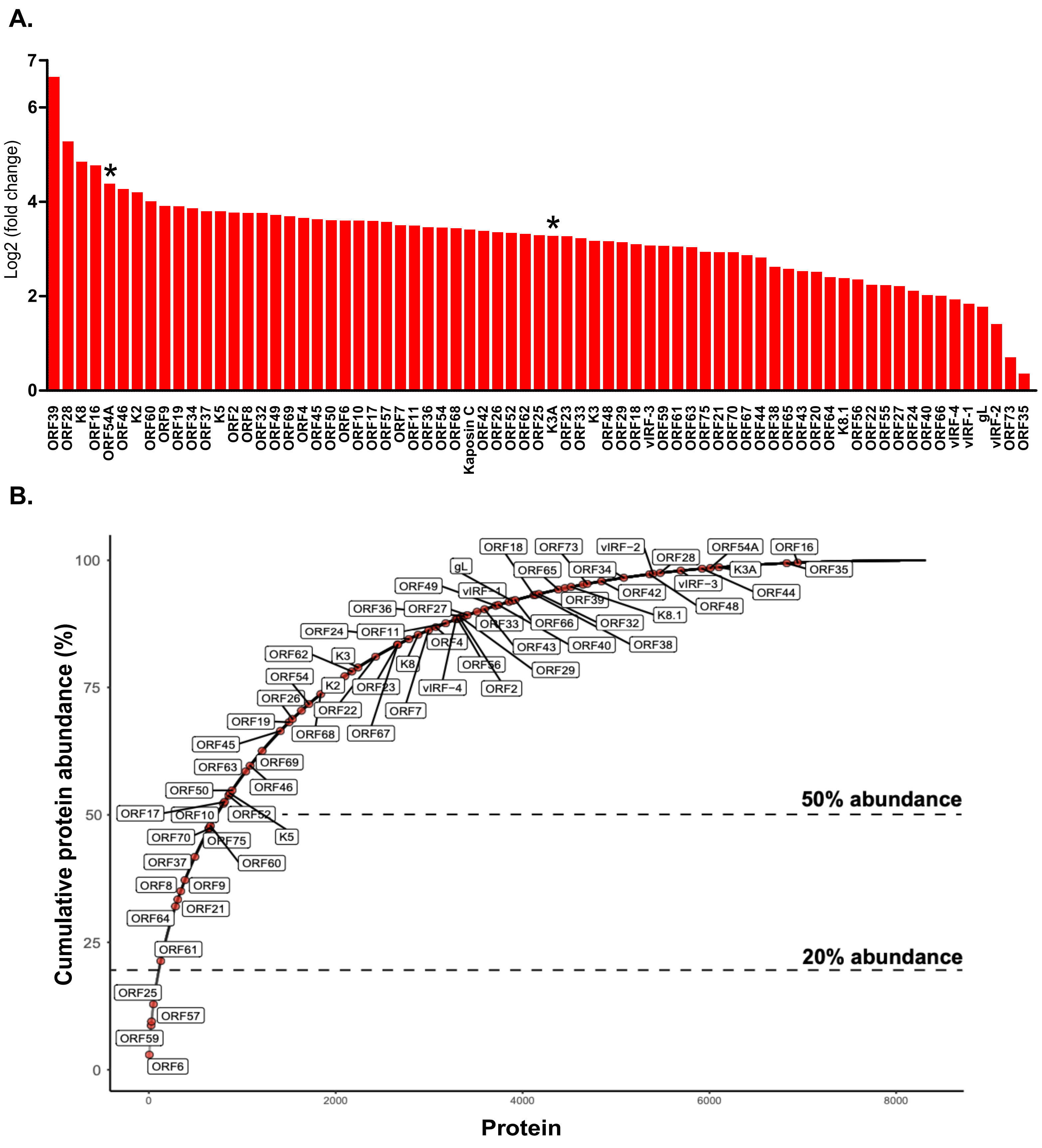

Figure S1. KSHV proteins identified in quantitative proteomics experiments. Related to Figure 1.
(A) The histogram shows fold change in abundance of the KSHV proteins, quantified in one of two proteomics experiments with two peptides and q < 0.05. An asterisk denotes alternative viral translational products reported in ribosome profiling study (Arias et al., 2014).
(B) A plot of cumulative sum of protein abundance versus proteins ranked by abundance (most abundant proteins are in the left bottom corner). Viral proteins are highlighted in red and annotated.

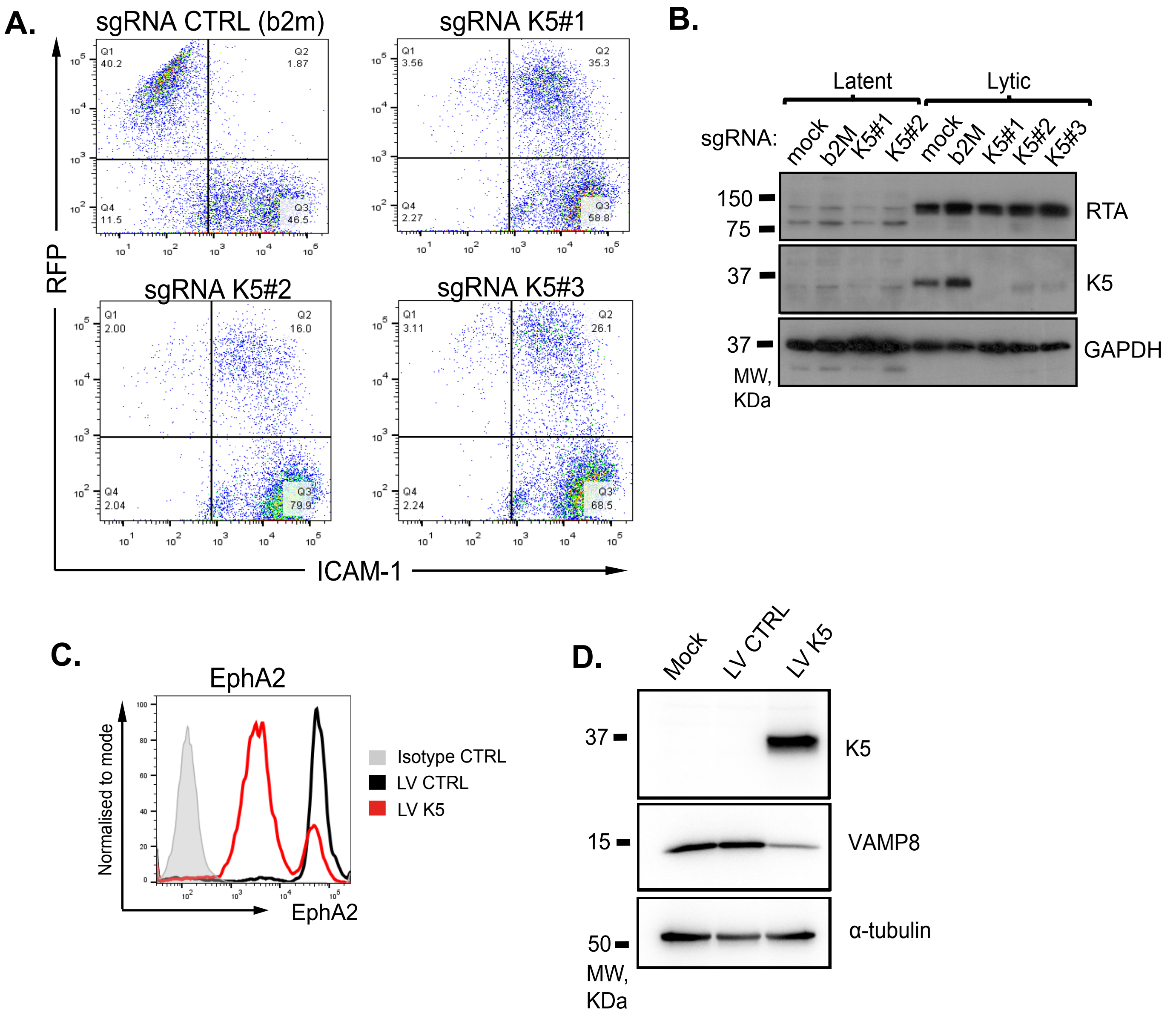

Figure S2. CRISPR Cas9-mediated depletion of KSHV K5 unravels novel K5 targets. Related to Figures 2 and 3.
(A) K5-specific sgRNAs rescue lytic KSHV-mediated downregulation of ICAM-1. HuAR2T.rKSHV.219 Cas9 cells were transduced and treated as indicated above, stained with ICAM-1-specific antibody and analysed by flow cytometry.
(B) KSHV K5 is depleted with CRISPR Cas9 and K5-specific sgRNAs. HuAR2T.rKSHV.219 Cas9 cells were transduced with lentivirus expressing individual KSHV K5-specific sgRNA or control (b2M) sgRNA, left untreated (latent) or treated with 'reactivation mix' to induce lytic KSHV cycle. Cells were harvested 65 h later and analysed by immunoblot with antibody specific for KSHV RTA and K5 proteins. GAPDH was used as the loading control.
(C) KSHV K5 downregulates EphA2 receptor. HuAR2T cells were transduced with control GFP (LV CTRL, black line) or K5 GFP (LV K5, red line) lentiviruses, harvested 72 hpt, stained with isotype control antibody (grey shading) or antibody specific for EphA2 and analysed by flow cytometry.
(D) KSHV K5 downregulates VAMP8. HuAR2T cells were transduced with control GFP (LV CTRL) or K5 GFP (LV K5) lentiviruses, harvested 72 h later, sorted on GFP+ and analysed by immunoblot with K5- and VAMP8-specific antibody. α- tubulin was used as the loading control.

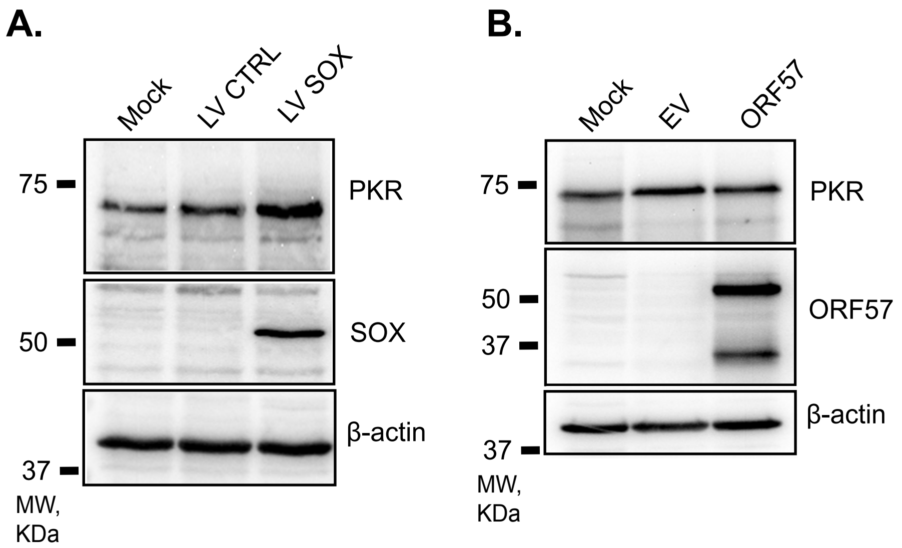

Figure S3. PKR is not downregulated by KSHV ORF37 (SOX) or ORF57. Related to Figure 4.
(A) HuAR2T cells were transduced with control lentivirus (LV CTRL) or lentivirus expressing KSHV ORF37 (LV SOX), harvested 60 h later and analysed with antibody specific for PKR and KSHV SOX protein. β-actin was used as the loading control.
(B) HEK293 cells were transfected with expression vectors encoding ORF57 or empty vector (EV), harvested 60 h later and analysed by immunoblot with antibody specific for PKR and KSHV ORF57.

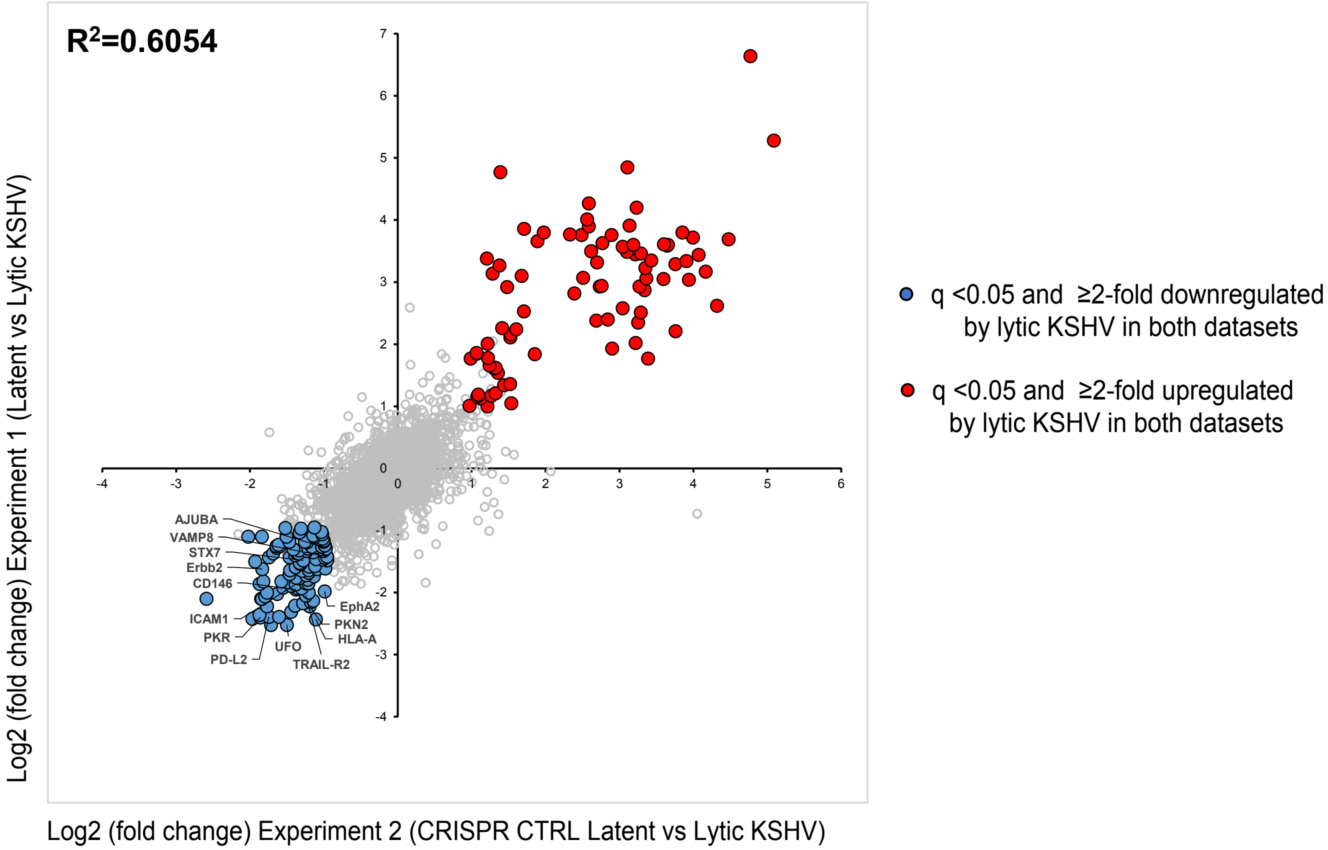

Figure S4. Scatterplot showing pairwise comparison from two independent proteomics experiments. Related to Figures 1 and 3. Each point represents a single protein, plotted by its log2 (fold change in abundance) in the experiment 1 (X axis, as shown in Figure 1D) vs experiment 2 (Y axis, as shown in Figure 3A). Annotated proteins were selected for validation by flow cytometry and/or immunoblot analysis (Figure 3F-H, Figure S3A,B, Figure 4B,D, Figure 5E,I,J).

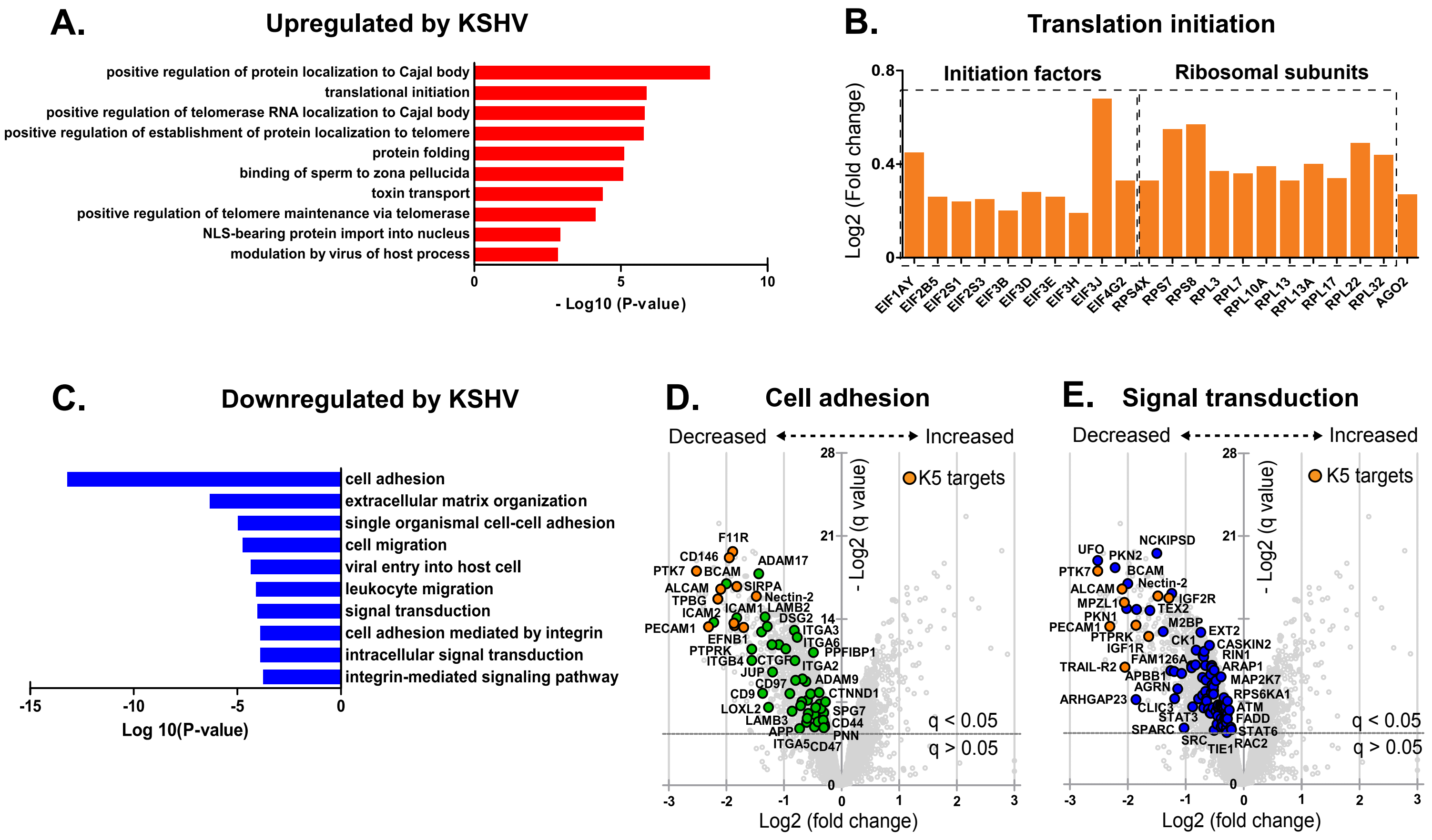

Figure S5. DAVID GO term analysis of cellular proteins from the category 'Biological process' dysregulated by lytic KSHV infection. Related to Figure 5. See also Table S4.

(A) Gene Ontology (GO) functional annotation terms enriched amongst upregulated proteins with $q < 0.05$ in cells with lytic vs latent KSHV infection from two proteomic experiments (Figures 1E and 3D). Ten most enriched GO terms (ranked by p-value) in the category 'Biological process' amongst upregulated proteins are shown.

(B) The histogram shows fold change in abundance of the proteins from the GO term 'Translation initiation'. Factors of translation initiation and ribosomal subunits are highlighted with dashed line squares.

(C) Ten most enriched GO terms (ranked by p-value) in the category 'Biological process' amongst downregulated proteins are shown.

(D, E) Downregulated proteins from the GO terms 'Cell adhesion' and 'Signal transduction' are highlighted, respectively as green and blue points on the scatterplots that display pairwise comparison between latent and lytic KSHV infection. Each point represents a single protein, plotted by its log2 (fold change in abundance) vs the statistical significance of that change. Orange points represent targets of KSHV K5 protein.

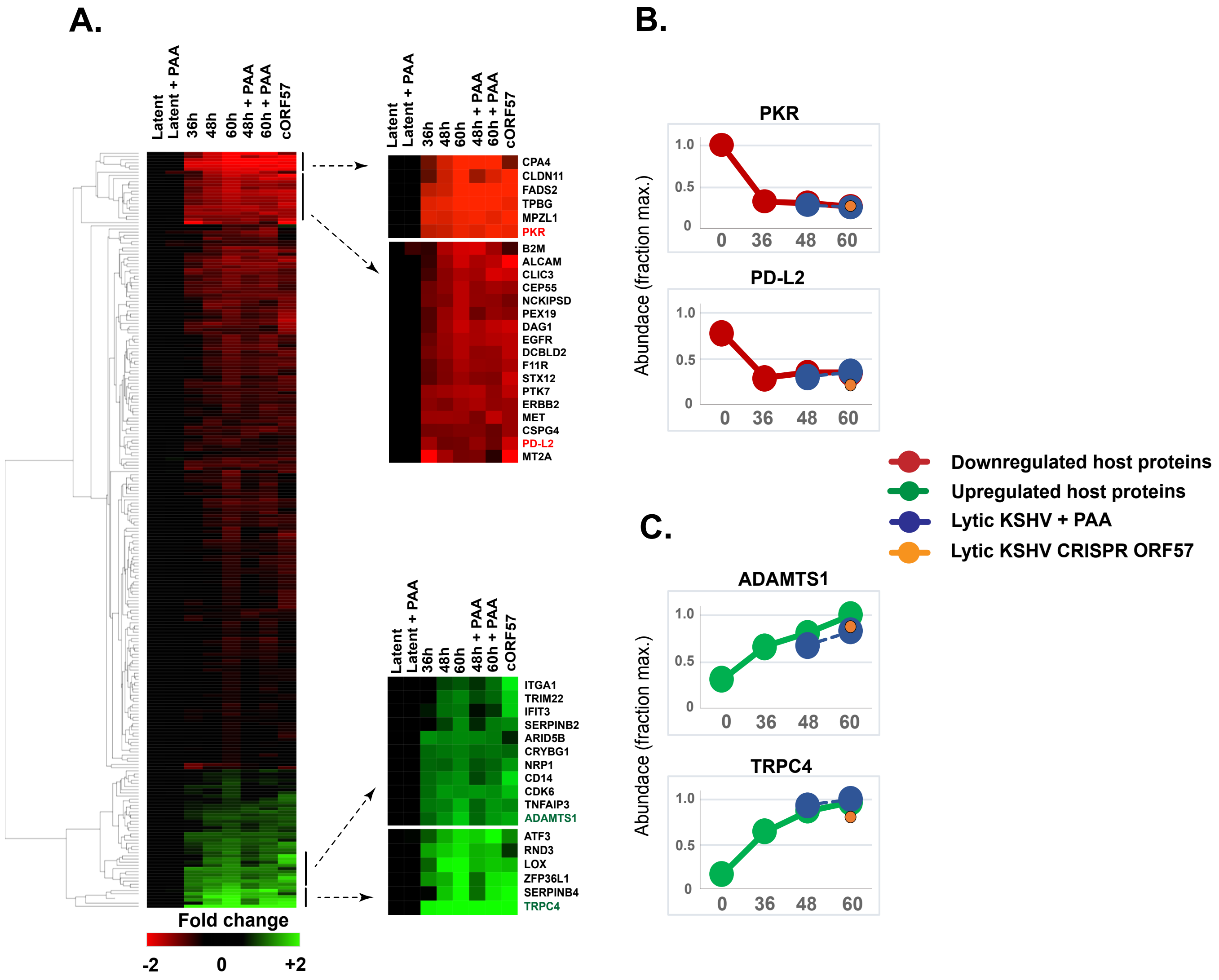

Figure S6. Kinetic profiling of cellular proteins in lytic KSHV infection. Related to Figure 6.
(A) Hierarchical cluster analysis of host cell proteins dysregulated by lytic KSHV infection. Heatmap diagram (left side) shows temporal kinetic (0,36,48,60 hours upon transduction), PAA-sensitivity and ORF57-dependency profiles for cellular proteins dysregulated by lytic KSHV. Rows and columns represent, respectively, individual cellular proteins and experimental samples. Cellular proteins 1.5-fold dysregulated by lytic KSHV infection in the proteomics experiment 3 (Figure 6A) and 1.5-fold dysregulated with $q < 0.05$ in the proteomics experiment 1 (Figure 1D) and experiment 2 (Figure 3A) were taken for cluster analysis. Enlarged segments of the heatmap (right side) show top two clusters of host proteins down- or upregulated by lytic KSHV infection.
(B, C) Kinetic profiles of host cell proteins dysregulated by lytic KSHV from the clusters shown in the Figure S6A. Protein abundance is calculated as a fraction of maximum TMT reporter ion intensity.

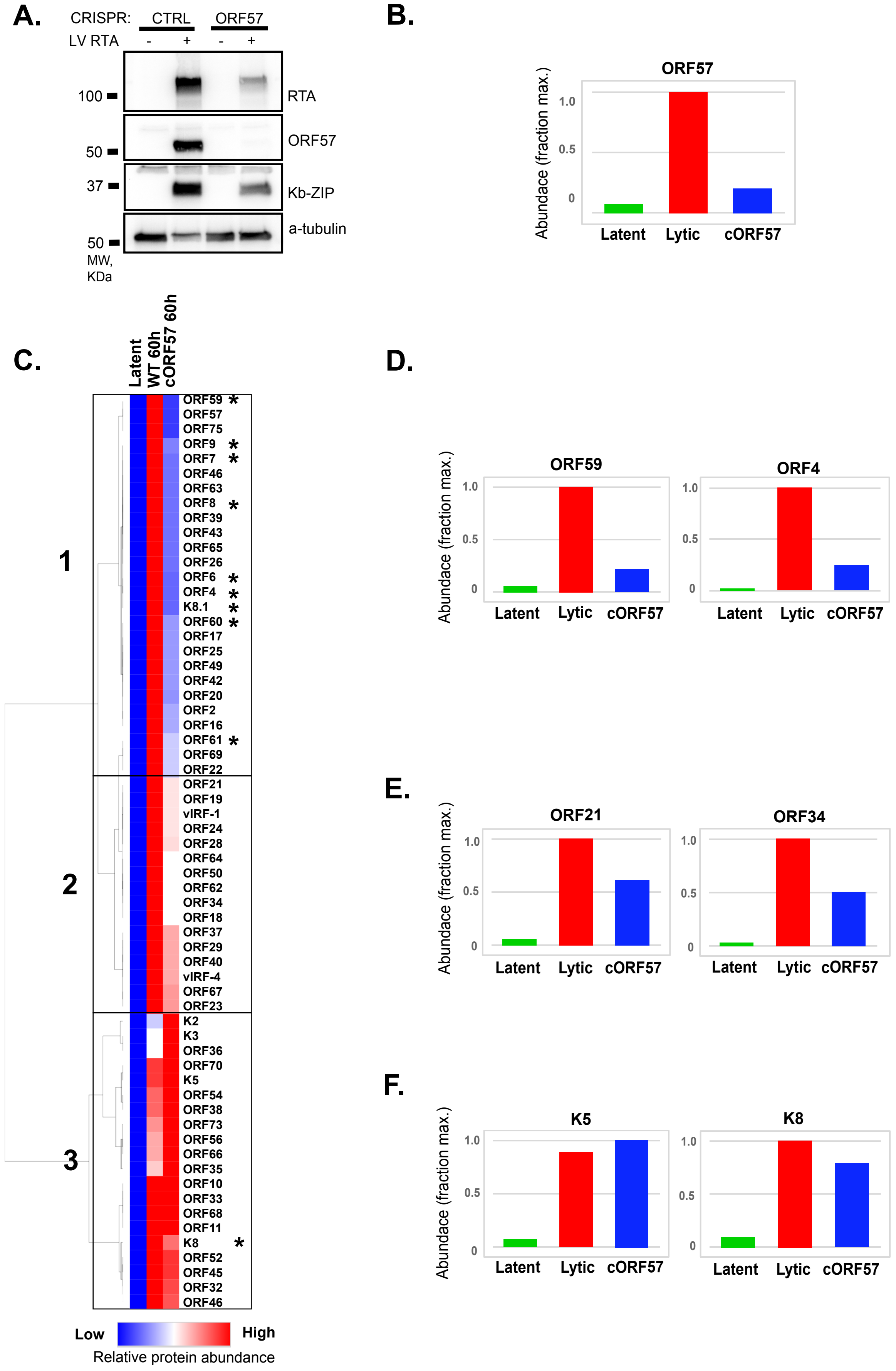

Figure S7. ORF57-dependent expression of viral ORFs. Related to Figure 6. (A) Immunoblot analysis of HuAR2T.rKSHV.219 Cas9 cells stably expressing control or ORF57-specific sgRNAs, transduced with LV RTA, harvested 60h later and probed with antibody against RTA, ORF57, Kb-ZIP proteins. (B) Relative abundance of ORF57 in the cells with KSHV latent (green), lytic control (blue) and lytic CRISPR ORF57 (red) infections. Protein abundance is calculated as a fraction of maximum TMT reporter ion intensity. (C) Hierarchical cluster analysis of all viral proteins quantified. Asterisks denote viral ORFs previously reported to be dependent on ORF57. Rows and columns represent, respectively, individual viral ORFs and experimental samples. Black squares show groups of viral proteins identified by hierarchical cluster analysis. (D-F) Relative abundances of the indicated viral proteins in the cells with KSHV latent (green), lytic control (blue) and lytic CRISPR ORF57 (red) infections.
