# Supplementary material for: Quantitative Proteomics Analysis of Lytic KSHV Infection in Human Endothelial Cells Reveals Targets of Viral Immune Modulation
Source: Cell Rep. 2020 Oct 13;33(2):108249. doi: 10.1016/j.celrep.2020.108249 (PMC7567700; doi:10.1016/j.celrep.2020.108249)
Supplement: Document S2. Article plus Supplemental Information [file mmc6.pdf]

Cell Reports

Resource

Quantitative Proteomics Analysis of Lytic KSHV Infection in Human Endothelial Cells Reveals Targets of Viral Immune Modulation

Graphical Abstract

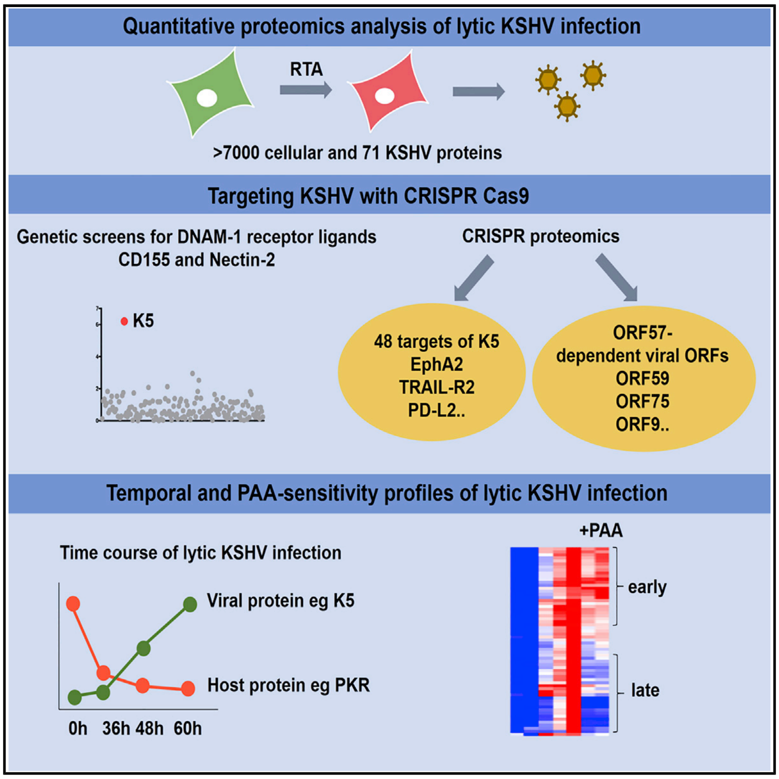

Highlights

- Quantitative proteomics identifies changes in host proteins induced by lytic KSHV
- Targeting KSHV with CRISPR identifies CD155 and Nectin-2 as KSHV K5 substrates
- Lytic KSHV downregulates protein kinase R in human endothelial cells
- Kinetic profiling of lytic KSHV unravels PAA-sensitive and ORF57-dependent proteins

Authors

Ildar Gabaev, James C. Williamson, Thomas W.M. Crozier, Thomas F. Schulz, Paul J. Lehner

Correspondence

ig329@cam.ac.uk (I.G.),
pjl30@cam.ac.uk (P.J.L.)

In Brief

Gabaev et al. describe how a human oncogenic herpesvirus, KSHV, changes the proteome of endothelial cells and modulates the host immune system. By targeting KSHV with CRISPR, they show that viral K5 protein downregulates ligands for the NK cell DNAM-1 receptor and antiviral protein PKR is depleted in an K5-independent manner.

Gabaev et al., 2020, Cell Reports *33*, 108249
October 13, 2020 © 2020 The Authors.
https://doi.org/10.1016/j.celrep.2020.108249

CellPress

Cell Reports

CellPress
OPEN ACCESS

Resource

Quantitative Proteomics Analysis of Lytic KSHV Infection in Human Endothelial Cells Reveals Targets of Viral Immune Modulation

Ildar Gabaev,[1,2,5,*] James C. Williamson,[1,2] Thomas W.M. Crozier,[1,2] Thomas F. Schulz,[3,4] and Paul J. Lehner[1,2,*]
[1]Department of Medicine, University of Cambridge, Hills Road, Cambridge CB2 0QQ, UK
[2]Cambridge Institute for Therapeutic Immunology and Infectious Disease (CITIID), University of Cambridge, Puddicombe Way, Cambridge CB2 0AW, UK
[3]Institute of Virology, Hannover Medical School, Carl-Neuberg-Straße 1, Hannover 30625, Germany
[4]German Center for Infection Research, Hannover-Braunschweig, Germany
[5]Lead Contact
*Correspondence: ig329@cam.ac.uk (I.G.), pjl30@cam.ac.uk (P.J.L.)
https://doi.org/10.1016/j.celrep.2020.108249

SUMMARY

Kaposi's sarcoma herpesvirus (KSHV) is an oncogenic human virus and the leading cause of mortality in HIV infection. KSHV reactivation from latent- to lytic-stage infection initiates a cascade of viral gene expression. Here we show how these changes remodel the host cell proteome to enable viral replication. By undertaking a systematic and unbiased analysis of changes to the endothelial cell proteome following KSHV reactivation, we quantify >7,000 cellular proteins and 71 viral proteins and provide a temporal profile of protein changes during the course of lytic KSHV infection. Lytic KSHV induces >2-fold downregulation of 291 cellular proteins, including PKR, the key cellular sensor of double-stranded RNA. Despite the multiple episomes per cell, CRISPR-Cas9 efficiently targets KSHV genomes. A complementary KSHV genome-wide CRISPR genetic screen identifies K5 as the viral gene responsible for the downregulation of two KSHV targets, Nectin-2 and CD155, ligands of the NK cell DNAM-1 receptor.

INTRODUCTION

Kaposi's sarcoma herpesvirus (KSHV) or HHV-8 (human herpesvirus 8) causes Kaposi's sarcoma (KS), a highly vascular tumor of lymphatic and blood vessels (Damania and Cesarman, 2013). KSHV is latent in healthy subjects but may reactivate in immunocompromised individuals with serious consequences. KS is one of the most common tumors in AIDS patients (Ganem, 2010) and is linked to two B cell malignancies: primary effusion lymphoma (PEL) and multicentric Castleman disease (Cesarman et al., 1995; Soulier et al., 1995). The seroprevalence of KSHV ranges from 50% in sub-Saharan Africa to 20%–30% in Mediterranean countries and less than 10% in most of North America, Europe, and Asia (Uldrick and Whitby, 2011).

Primary KSHV infection results in latent viral infection, the default state with only few viral genes and microRNAs (miRNAs) expressed (Ganem, 2010; Schulz, 2006). Upon stimulation, the latent virus undergoes lytic reactivation that *in vivo* is triggered by viral co-infections or immunosuppression (reviewed in Aneja and Yuan, 2017). In the laboratory, viral reactivation is typically induced by treatment of latently infected cells with chemical compounds such as phorbol esters and histone deacetylase (HDAC) inhibitors. During lytic-stage KSHV infection, the repertoire of viral gene products is expressed in a temporal cascade, resulting in viral replication and the release of new virions.

The main cell in KS tumors is the highly proliferative spindle cell, which expresses both lymphatic and vascular endothelial markers (Gramolelli and Schulz, 2015; Ojala and Schulz, 2014). These cells also share features with mesenchymal cells as a result of the endothelial-to-mesenchymal transition process (EndMT). Up to 90% of spindle cells in KS tumors harbor latent KSHV genomes, with a small proportion undergoing lytic-stage viral reactivation (Katano et al., 2000), and both stages of infection contribute to angiogenic phenotypes (Manners et al., 2018).

The KSHV-RTA (replication and transcription activator) viral protein is both essential and sufficient for viral reactivation (Lukac et al., 1998, 1999; Sun et al., 1998), and it plays a key role in the latent- to lytic-stage viral switch. To maintain the latent, repressive viral state requires silencing of lytic promoters, particularly the RTA promoter, because RTA is the first protein to be expressed in lytic-phase infection and initiates the transcriptional activation of multiple downstream viral genes. The RTA promoter is inhibited by the LANA latent viral protein (Lan et al., 2004, 2005; Lu et al., 2006), as well as host cell silencing complexes (Sun et al., 2014; Yada et al., 2006). The switch to lytic-phase infection is associated with chromatin remodeling (Lu et al., 2003; Hopcraft et al., 2018) and auto-activation of the RTA promoter (Deng et al., 2000), resulting in the transcriptional activation of multiple downstream lytic genes (Bu et al., 2008).

© 2020 The Authors.
This is an open access article under the CC BY license (http://creativecommons.org/licenses/by/4.0/).

During lytic KSHV infection, the host cell expresses more than 80 viral proteins, and KSHV, like other herpesviruses, has evolved multiple immunomodulatory strategies. The best-characterized KSHV-encoded immunoevasins are the K3 and K5 proteins, which downregulate multiple immunoreceptors, including major histocompatibility complex class I (MHC class I) molecules, and protect virus-infected cells from immune responses mediated by cytotoxic T cells and natural killer (NK) cells (Boname and Lehner, 2011; Coscoy and Ganem, 2000; Duncan et al., 2006; Ishido et al., 2000a, 2000b; Thomas et al., 2008a, 2008b). Lytic KSHV replication is also sensed by components of the host innate immune system, e.g., IFI16 (Kerur et al., 2011), MxB (Crameri et al., 2018), and IFIT proteins (Li and Swaminathan, 2019). KSHV in turn counteracts host cell restriction factors, e.g., IFI16 (Roy et al., 2016), and sensing pathways, e.g., cGAS-STING (Ma et al., 2015; Wu et al., 2015; Zhang et al., 2016).

Double-stranded RNA sensors such as RIG-I and MDA-5 also play an important role in lytic KSHV infection (Inn et al., 2011; West et al., 2014; Zhang et al., 2018; Zhao et al., 2018). Herpesviruses have double-stranded DNA (dsDNA) genomes and produce dsRNA as a by-product of their replication (Jacquemont and Roizman, 1975) as detected in cells infected by herpes simplex virus (HSV) 1 (Weber et al., 2006) and KSHV (West et al., 2014). The double-stranded RNA-dependent protein kinase R (PKR) is a critical host cell factor in the recognition of virus-derived dsRNA (reviewed in Glaunsinger, 2015). Upon dsRNA recognition, PKR auto-phosphorylates, dimerizes, and subsequently phosphorylates eukaryotic translation initiation factor 2α(eIF2α), leading to inhibition of protein synthesis in virus-infected cells. The importance of the antiviral function of PKR is emphasized by the finding that many RNA and DNA viruses, including multiple *Herpesviridae* family members, counteract PKR-mediated shutoff of protein synthesis. Depletion of PKR (e.g., through proteasome degradation) helps prevent host protein synthesis shutoff, as shown for several viruses, e.g., mouse adenovirus (Goodman et al., 2019), Toscana virus (Kalveram and Ikegami, 2013), and Rift Fever Valley virus (Mudhasani et al., 2016). α- and β-herpesviruses counteract PKR by (1) preventing its activation by direct binding of viral proteins (Budt et al., 2009; Hakki et al., 2006; Valchanova et al., 2006; Ziehr et al., 2016), (2) degrading or shielding dsRNA (Poppers et al., 2000; Sciortino et al., 2013), and (3) interfering with phosphorylation of eIF2α (Li et al., 2011). With γ-herpesviruses, two lytic Epstein-Barr virus (EBV) gene products, SM and BILF, prevent PKR activation (Beisser et al., 2005; Poppers et al., 2003), whereas two lytic KSHV proteins are reported to interfere with PKR function. Viral interferon regulatory factor-2 (vIRF-2) blocks eIF2α phosphorylation (Burýsek and Pitha, 2001), whereas open reading frame (ORF) 57 interacts with PKR and inhibits dsRNA binding and PKR autophosphorylation (Sharma et al., 2017).

The expression of KSHV gene products in lytic-phase infection has been characterized for both mRNA (Arias et al., 2014; Chandriani et al., 2010) and protein expression (Dresang et al., 2011). Although host cell transcriptional changes following lytic KSHV infection have been described (Di Bartolo and Cesarman, 2004; Chandriani and Ganem, 2007) there has been no analysis of changes to the host cell proteome upon lytic KSHV infection. Here, we undertake a systematic, unbiased, quantitative proteomic analysis, from two independent proteomics experiments, of lytic KSHV-induced changes in the cellular proteome of human endothelial cells, a natural target of KSHV infection. Using tandem mass tag (TMT)-based proteomics, we identify 71 viral proteins and >7,000 cellular proteins, of which 291 are >2-fold downregulated upon KSHV reactivation, including candidate host resistance/restriction factors. We provide a time course of viral and cellular protein expression following KSHV reactivation. Altogether, these readily searchable datasets provide a resource for viral and host cell protein changes during the course of latent KSHV reactivation. We also show that CRISPR-Cas9-based genetics efficiently depletes KSHV genes from the viral episomal genome and use this technology to identify viral genes responsible for the observed cellular phenotypes. We show that K5 is the dominant KSHV-encoded immunomodulatory viral protein, affecting expression of at least 48 proteins in endothelial cells. Among K5 targets, we identify ligands for the NK cell DNAM-1 receptor and show that PKR, a key host antiviral protein, is downregulated by KSHV in a K5-independent manner.

RESULTS

A Proteomics-Compatible System for KSHV Reactivation in Endothelial Cells

To investigate how lytic KSHV infection remodels the host cell proteome, we needed to establish a KSHV-reactivation system compatible with proteomic-based approaches. We chose to study KSHV-infected endothelial cells, which like B cells, represent physiologically relevant *in vivo* targets of KSHV infection (Alkharsah et al., 2011). We used HuAR2T-tert, a cell line derived from conditionally immortalized human umbilical vein endothelial cells (May et al., 2010) that harbors latent recombinant KSHV encoding GFP and RFP (HuAR2T.rKSHV.219) and is engineered to report the presence (GFP) and reactivation (RFP) of virus (Vieira and O'Hearn, 2004). KSHV is reactivated from these endothelial cells by delivery of exogenous KSHV-RTA to the latent population. Traditional methods of reactivation, using RTA-encoding baculovirus and HDAC inhibitors, rarely exceed >40% KSHV lytic infection, which is not compatible with reliable proteomic data that require analysis of cells that are more than 85% infected. To overcome this limitation, we transduced HuAR2T.rKSHV.219 cells with a lentiviral vector (LV) expressing KSHV-RTA (LV RTA) without HDAC inhibitors. Following RTA transduction (Figure 1A), immunoblot analysis detected early (Kb-ZIP) and late (K8.1) lytic KSHV proteins, indicating the switch from latent- to lytic-phase reactivation (Figure 1B). Despite some cytopathic effect at 48 h posttransduction (hpt), most (>90%) reactivated cells harvested at 65 hpt remained adherent. To enrich for reactivated cells, we performed fluorescence-activated cell sorting (FACS) of the distinct, RFP-bright population (Figure 1C, 48 and 72 hpt) of adherent (viable) cells to obtain a 100% homogeneous lytic KSHV population. A BFP-expressing, lentivirus-transduced KSHV latent cell population controlled for effects induced by transduction of lentivirus alone.

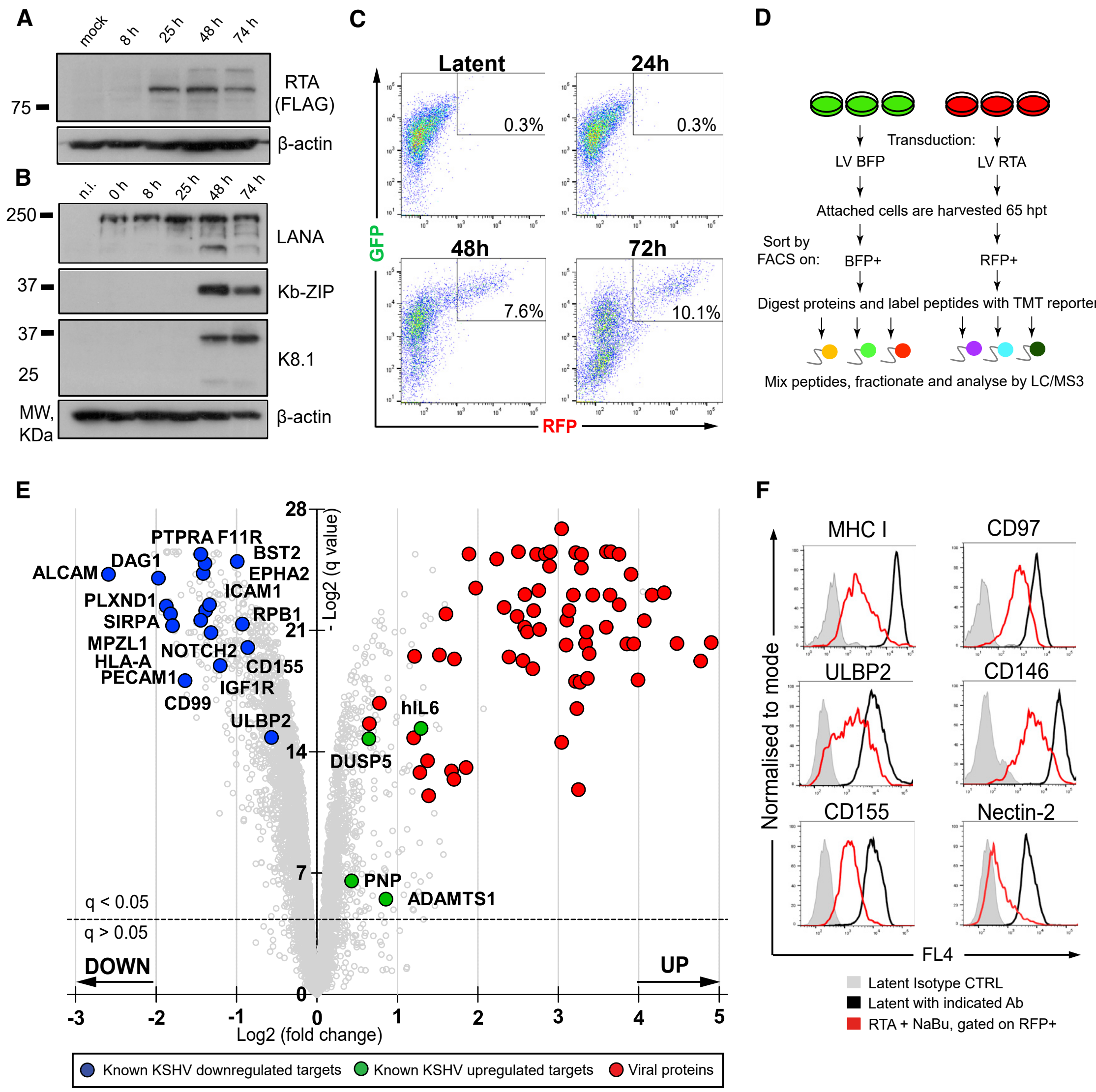

Figure 1. Lytic KSHV Induces Massive Changes in the Proteome of Endothelial Cells

(A and B) Immunoblot analysis of HuAR2T.rKSHV.219 cells transduced with LV RTA, harvested at indicated time points (8, 25, 48, and 74 h), and probed with the indicated antibody.

(C) Flow cytometry analysis of HuAR2T.rKSHV.219 cells mock-transduced (latent) or transduced with LV RTA and harvested at the indicated time points (24, 48, and 72 h).

(D) Schematic overview of the quantitative proteomics analysis of the cells with latent versus lytic KSHV infection. HuAR2T.rKSHV.219 cells were transduced with LV RTA or control LV BFP, sorted on BFP+ or RFP+, and analyzed by mass spectrometry (MS).

(E) Scatterplot displays pairwise comparison between latent and lytic KSHV infection. Each point represents a single protein, plotted by its log2 (fold change in abundance) versus the statistical significance (q value) of that change. Value was corrected for multiple hypothesis testing using the method of Benjamini-Hochberg. Dotted line: q = 0.05.

(F) Flow cytometry analysis of HuAR2T.rKSHV.219 Cas9 cells untreated or treated with reactivation mix and stained with the indicated antibody.

See also Figure S1 and Table S1.

Lytic KSHV Infection Induces Massive Changes in the Proteome of Endothelial Cells

Using this experimental setup, we obtained a homogeneous population of KSHV-reactivated cells and applied multiplex TMT-based proteomics to determine the cellular proteomic changes that occur following KSHV reactivation. Each sample was performed in triplicate (Figure 1D). We quantified >7,300 cellular and viral proteins. More than 1,100 cellular proteins (~15%) were significantly ($q < 0.05$) upregulated, including four host gene products known to be induced by the KSHV vGPCR protein: DUSP5, hIL6, ADAMTS1, and PNP (Glaunsinger and Ganem, 2004a), thus validating our approach. More than 3,000 host cell proteins (~41%) showed a significant decrease in expression, including proteins previously shown to be depleted in lytic

KSHV infection: MHC class I (Coscoy and Ganem, 2001; Ishido et al., 2000b; Stevenson et al., 2000), BST2/tetherin (Bartee et al., 2006), RNA polymerase II (RNA Pol II) subunit RPB1 (Chen et al., 2017), and NK cell receptor ligands ULBP2 and CD155 (Madrid and Ganem, 2012) (Figure 1E; Table S1). Furthermore, KSHV-downregulated proteins included several known KSHV-K5 targets (Timms et al., 2013). We were able to confirm lytic KSHV-mediated downregulation of MHC class I, ULBP2, and CD155 by flow cytometry, validating our proteomics approach (Figure 1F, left column). Downregulation of three targets of lytic KSHV—adhesion proteins CD146 and CD97, as well as Nectin-2, a ligand of NK cell DNAM-1 receptor (Figure 1F, right column)—was also confirmed.

Viral Proteins Identified by Quantitative Proteomics Analysis

In addition to host cellular proteins, we quantified 71 canonical viral proteins (>80% KSHV proteome) and two alternative translation products, KSHV ORF54A and K3A, which were previously detected among 63 non-canonical viral products in ribosome profiling analysis of lytic KSHV infection (Arias et al., 2014) (Figures 1E and S1A). We also measured cumulative protein abundance in the cells with lytic KSHV infection. The most abundant lytic proteins were ORF25, ORF57, ORF59, and ORF6 (Figure S1B). Together with 200 cellular proteins, these 4 viral proteins make up 20% of total protein abundance in the cell. The functions of ORF25, ORF57, and ORF59 are well defined: ORF25 is a major capsid protein that antagonizes p53-mediated apoptosis (Chudasama et al., 2015), ORF57 is a potent posttranscriptional regulator of lytic KSHV gene expression (Majerciak and Zheng, 2015; Vogt and Bohne, 2016), and ORF59 is a viral DNA polymerase processivity factor that transports viral DNA polymerase into the nucleus for efficient KSHV DNA synthesis (Chen et al., 2005). The role of ORF6 is less clear. Recent reports indicate that ORF6 is essential for lytic KSHV replication (Peng et al., 2014), binds to single-stranded DNA (ssDNA), and might be involved in viral DNA replication (Ozgur and Griffith, 2014).

Genetic Screen with a KSHV Genome-wide Library Identifies K5 as the Viral Gene Responsible for Downregulation of DNAM-1 Ligands

The cell-surface proteins Nectin-2 and CD155 are both ligands for the DNAM-1-activating NK cell receptor (Bottino et al., 2003) and were found to be downregulated by KSHV. Our proteomics experiment did not identify the viral gene or genes responsible for downregulation of these cellular proteins. An unbiased genetic approach using a CRISPR-Cas9 sgRNA library of KSHV-encoded genes provides a potentially powerful means to identify viral genes responsible for cellular phenotypes. However, because multiple copies of episomal KSHV are expressed in each cell (Dubich et al., 2019) we needed to determine whether CRISPR-Cas9 could successfully target viral genes. sgRNAs specific for ORF45 (immediate early), K5 (early), ORF34 (early late), and K8.1 (late) lytic KSHV genes were transduced into endothelial cells expressing CRISPR-Cas9, and expression of the respective viral gene products was analyzed during lytic-stage infection (Figure 2A). Protein expression of ORF45, K5, and K8.1 genes was abrogated by their respective sgRNAs (Figure 2A, lanes 6–8). ORF34 expression could not be analyzed because of the lack of a specific antibody. Because ORF34 is essential for late KSHV gene expression (Nishimura et al., 2017), abrogation of K8.1 expression (Figure 2A, lane 5) serves as a proxy for successful ORF34 depletion. Levels of the early Kb-ZIP protein were unaffected in all samples. Having confirmed that CRISPR-Cas9 specifically and effectively knocks out lytic genes from viral genomes, we designed a KSHV genome-wide sgRNA library targeting 143 KSHV genes and 12 miRNAs with up to 10 sgRNAs per gene for use in linking cellular phenotypes to the responsible viral gene or genes (Table S2). This library was used to screen for the KSHV-encoded gene or genes that target Nectin-2 and CD155 for degradation. Latent HuAR2T.rKSHV.219 Cas9 cells were transduced with the pooled sgRNA library to individually deplete viral ORFs and miRNAs (Figure 2B). Following lytic KSHV activation with RTA lentivirus, we used FACS to enrich the population of rare lytic cells that failed to downregulate Nectin-2 and CD155. Sequencing of the integrated sgRNAs from this enriched population identified K5 as the single viral ORF responsible for downregulation of Nectin-2 and CD155 (Figures 2C and 2D). Subsequent flow cytometry analysis confirmed that K5-specific sgRNAs completely (Nectin-2) or partially (CD155) rescued downregulation of these ligands by lytic KSHV (Figure 2E). Subsequent analysis showed that expression of K5 with lentivirus decreased cell-surface expression of both receptors (Figure 2F). We conclude that a CRISPR-Cas9 sgRNA library of KSHV-encoded genes is an effective way of identifying viral genes responsible for cellular phenotypes and that K5 is both necessary and sufficient for downregulation of the DNAM-1 ligands Nectin-2 and CD155.

Proteomic Analysis of CRISPR-Cas9-Modified Viral Genome Identifies Targets of KSHV K5 in Endothelial Cells

K5 is a potent KSHV-encoded E3 ligase with broad target specificity (Ashizawa et al., 2012; Boname and Lehner, 2011). The identification of K5 as the viral gene product responsible for downregulation of Nectin-2 and CD155 was unanticipated, because two previous high-throughput proteomic-based studies of HeLa and KBM7 cells (Bartee et al., 2006; Timms et al., 2013) did not identify these K5 targets. We reasoned that there might be additional K5 substrates in endothelial cells and set out to analyze how K5 affects the host cell proteome in the context of lytic KSHV infection. We wanted to compare endothelial cells infected with wild-type (WT) and K5-deficient KSHV, in which K5 was knocked out using CRISPR-Cas9. Three K5-specific sgRNAs were identified from the CRISPR KSHV library, which efficiently rescued downregulation of ICAM-1, a well-characterized K5 target (Figure S2A) (Coscoy and Ganem, 2001). K5 antibody-specific immunoblot analysis of the HuAR2T.rKSHV.219 Cas9 cells confirmed depletion of K5 in the samples harboring K5-specific sgRNAs, but not control (β2-microglobulin [β2m]) sgRNAs (Figure S2B). This efficient depletion of K5 allowed us to perform a differential proteomics analysis, comparing WT KSHV-infected cells with CRISPR K5 knockout KSHV-infected cells. Using the experimental setup (Figure 3A), we performed a 9-plex TMT proteomics in

CellPress
OPEN ACCESS

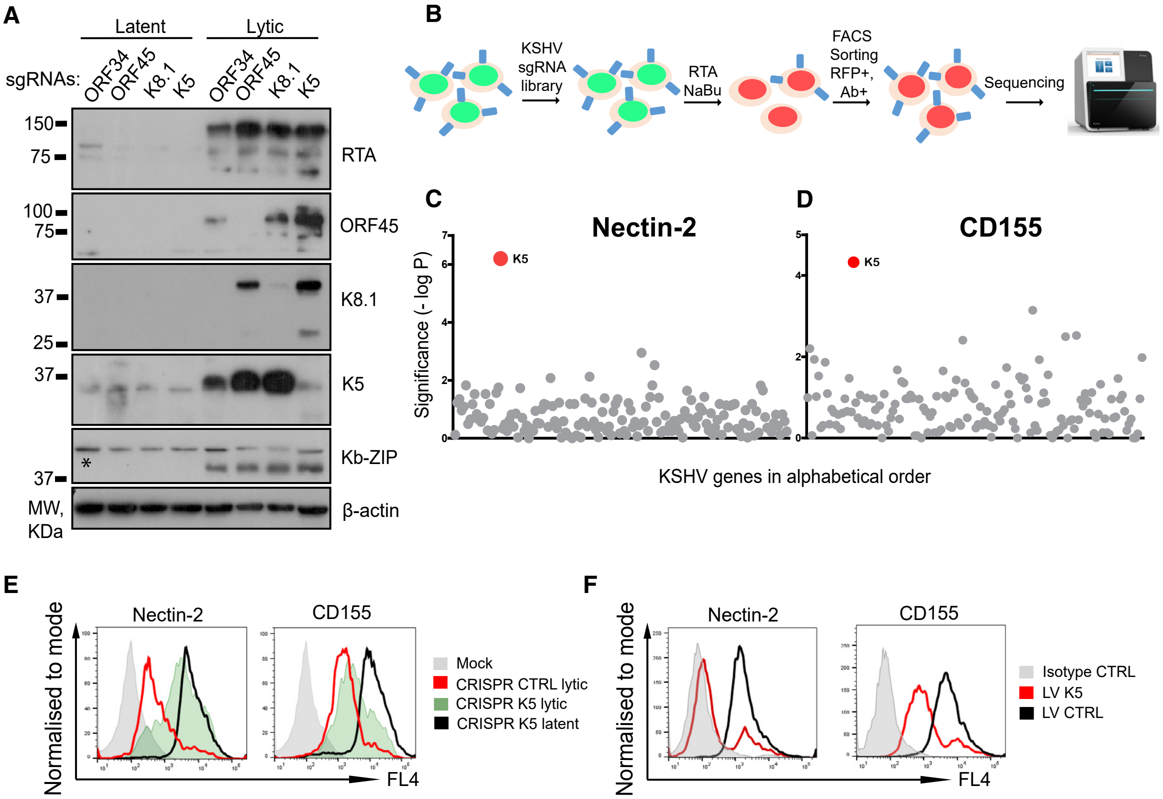

Figure 2. KSHV CRISPR Library Screen Identifies K5 as ORF Responsible for Downregulation of the NK Cell Receptor Ligands Nectin-2 and CD155

(A) Immunoblot analysis of HuAR2T.rKSHV.219 Cas9 cells harboring sgRNAs specific for indicated viral ORFs untreated or treated with reactivation mix and probed with the indicated antibodies. A non-specific band is labeled with an asterisk.

(B) Schematic flowchart of the genetic screen with the KSHV CRISPR library. HuAR2T.rKSHV.219 Cas9 cells were transduced with the sgRNA library followed by lytic KSHV cycle induction and stained with CD155- or Nectin-2-specific antibody. Lytic (RFP+) and CD155- or Nectin-2-high cells were selected by FACS and used for DNA extraction and sequencing.

(C and D) Genetic screens with the KSHV CRISPR library identify K5 as the ORF responsible for downregulation of Nectin-2 and CD155. Each dot represents a single KSHV ORF or miRNA plotted by the statistical significance (-log p value) of the sgRNA enrichment.

(E) CRISPR-mediated K5 knockout leads to full (Nectin-2) or partial (CD155) rescue of lytic KSHV-induced downregulation of the proteins from the cell surface. Flow cytometry analysis of HuAR2T.rKSHV.219 Cas9 cells harboring control or K5-specific sgRNAs untreated or treated with reactivation mix and stained with the indicated antibody.

(F) Flow cytometry analysis of HuAR2T cells transduced with LV K5 or control LV and probed with the indicated antibody.

HuAR2T.rKSHV.219 Cas9 cells and compared the following: (1) KSHV latent cells, (2) KSHV lytic cells transduced with control sgRNAs, and (3) KSHV lytic cells transduced with a combination of two K5-specific sgRNAs. Each batch was performed in triplicate. We identified 8,238 host cell proteins in the three groups, and K5 was the only viral protein depleted in the CRISPR K5 population, confirming the specificity of KSHV genome targeting and validating our approach (Figure 3C). Forty-eight host proteins were significantly ($q < 0.1$) downregulated by control lytic KSHV infection when compared with CRISPR K5 virus (as represented by blue dots) (Figures 3C–3E; Table S3). Forty-one of the 48 proteins were among 291 cellular substrates that showed >2-fold downregulation by control lytic KSHV infection. Thirty of the 48 proteins were previously reported as K5 substrates, including well-characterized targets such as MHC class I (Coscoy and Ganem, 2000; Ishido et al., 2000b), ICAM-1 (Coscoy and Ganem, 2001), PECAM (Mansouri et al., 2006), ALCAM, BST2/tetherin (Bartee et al., 2006), HFE (Rhodes et al., 2010), and K5 targets from a recent proteomic study (Timms et al., 2013), e.g., members of the Ephrin and Plexin receptor families. Eighteen of the K5 substrates were not previously reported. In addition to Nectin-2 and CD155, plasma membrane proteins included receptor tyrosine kinases (DDR2, ROR2, and Erbb2), receptor-type tyrosine-protein phosphatases (PTPRJ and PTPRE), receptors for cytokines and growth factors (IL10RB, TRAIL-R2, and IGF2R), zinc transporter SLC39A3, adhesion molecule CD146, surface hyaluronidase TMEM2, and PD-L2, a ligand for the PD-1 receptor. Using flow cytometry, we confirmed the K5-dependent downregulation of 4 of these cell-surface targets (Erbb2, CD146, PD-L2, and TRAIL-R2) (Figure 3F). EphA2 is particularly relevant, because it is a cell-surface entry receptor for KSHV (Hahn et al., 2012) and its downregulation by K5 was confirmed by both immunoblot (Figure 3G) and flow cytometry (Figure S2C). Other K5 substrates included three members of the SNARE

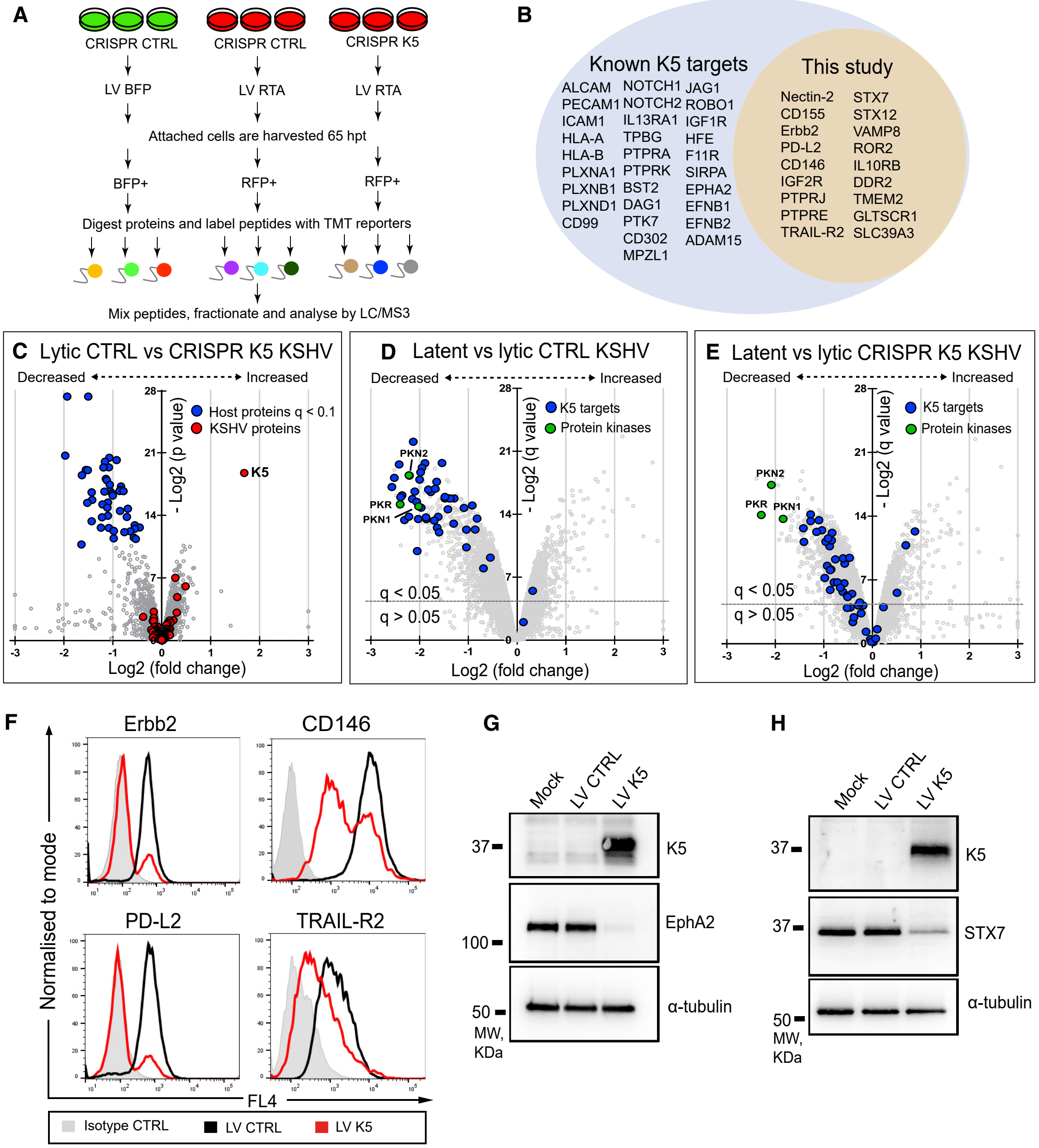

Figure 3. CRISPR Proteomics Identifies 48 KSHV K5 Targets in Endothelial Cells

(A) Schematic overview of the quantitative proteomic analysis of cells with lytic versus latent KSHV infection. HuAR2T.rKSHV.219 Cas9 cells harboring control or K5-specific sgRNAs were transduced with LV RTA or control LV BFP, sorted on BFP+ or RFP+, and analyzed by MS.

(B) KSHV K5 protein significantly ($q < 0.1$) downregulates 48 proteins in endothelial cells, of which 30 are known K5 targets (left side of blue circle) and 18 proteins represent K5 targets identified in this study (yellow circle).

(C–E) Scatterplots display pairwise comparison between lytic control and CRISPR K5 (C), latent and lytic control (D), and latent and lytic CRISPR K5 (E) KSHV infections. Each point represents a single protein, plotted by its log2 (fold change in abundance) versus the statistical significance (p and q value) of that change. Values were corrected for multiple hypothesis testing using the method of Benjamini-Hochberg. Dotted lines: $q = 0.05$.

(F) Flow cytometry analysis of HuAR2T cells transduced with LV K5 or control LV and stained with isotype control antibody or antibody specific for the indicated proteins.

(G and H) Immunoblot analysis of HuAR2T cells transduced with LV K5 or control LV, sorted on GFP+, and probed with the indicated antibody.

See also Figure S2 and Table S3.

CellPress
OPEN ACCESS

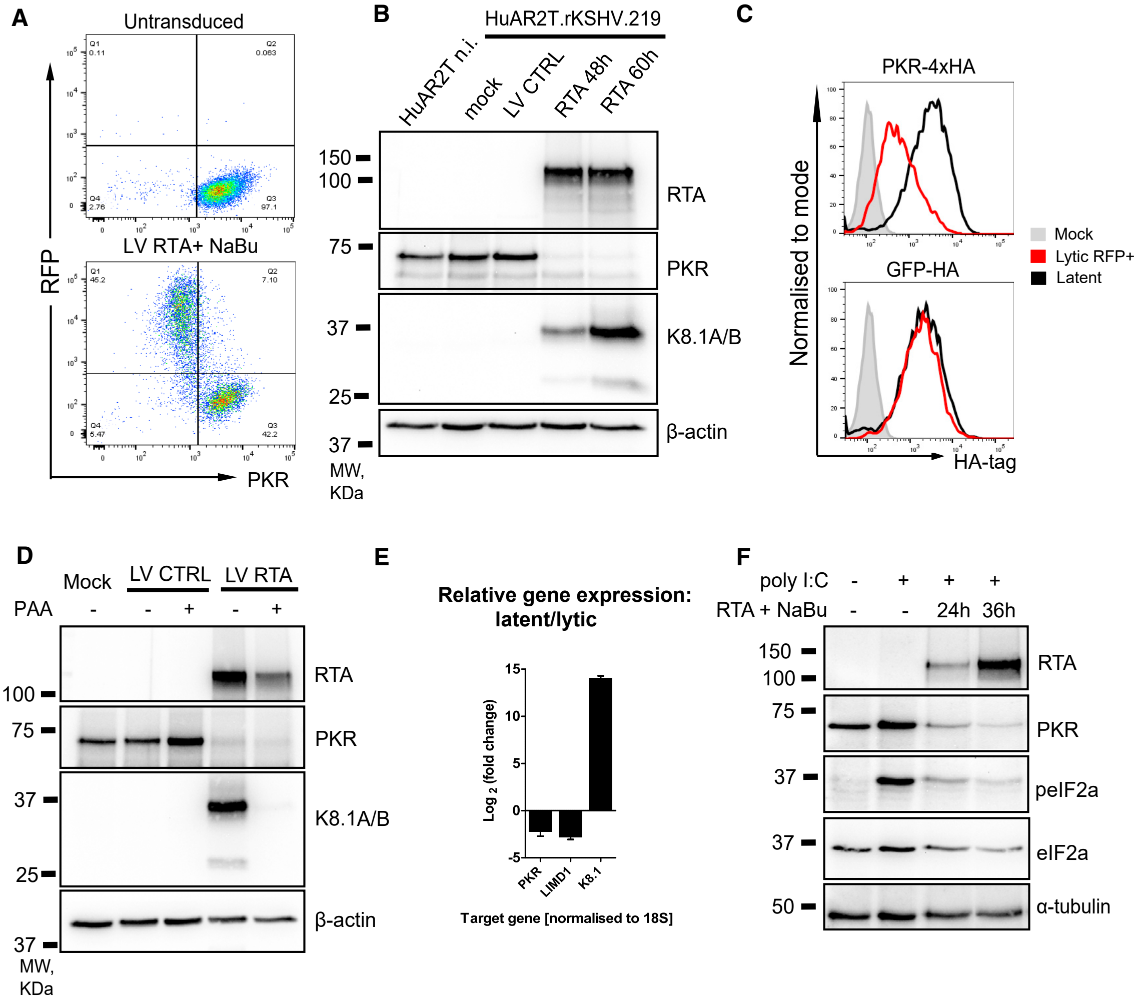

Figure 4. Protein Kinase R Is Downregulated by Lytic KSHV Infection

(A) Flow cytometry analysis of HuAR2T.rKSHV.219 cells with latent (top panel) and lytic (bottom panel) KSHV infection.
(B) Immunoblot analysis of uninfected HuAR2T cells (lane 1) and HuAR2T.rKSHV.219 cells (lanes 2–5) in latent and lytic stages of KSHV infection.
(C) Flow cytometry analysis of HuAR2T.rKSHV.219 cells with stable expression of PKR-4xHA and GFP-HA untreated or treated with reactivation mix and stained with anti-HA tag antibody. The signal from the HA tag is compared between the RFP+ population (red line) and the latent cells (black line).
(D) Immunoblot analysis of HuAR2T.rKSHV.219 cells transduced with LV RTA or control LV, sorted on RFP+, and probed with the indicated antibody.
(E) qRT-PCR analysis of the host and viral gene expression in cells with the lytic versus latent stage of infection. Data are represented as mean ± SEM.
(F) Immunoblot analysis of HuAR2T.rKSHV.219 Cas9 cells treated with RTA reactivation mix and poly(I:C) and probed with the indicated antibody.

protein family (STX7, STX12, and VAMP8), of which two were validated in HuAR2T cells transduced with K5 lentivirus (Figures 3H and S2D). These results demonstrate the utility of the CRISPR-Cas9 system in efficiently generating viral gene knockouts despite the multiple episomal copies of KSHV genomes in each infected cell. They also highlight the critical role of K5 in remodeling the host cell proteome during lytic KSHV infection.

An Early Lytic KSHV Factor Depletes PKR

A prominent lytic KSHV target was PKR; along with protein kinase C (PKC)-related serine/threonine-protein kinases PKN1 and PKN2, PKR was downregulated in a K5-independent manner (Figures 3D and 3E). PKR is a critical host antiviral protein (García et al., 2007), and its downregulation by lytic KSHV likely represents a crucial step for virus reactivation. Flow cytometry validated the decrease in PKR levels in the RFP-positive (KSHV lytic) cell population compared with RFP-negative (latent) HuAR2T.rKSHV.219 cells (Figure 4A). Next, we assessed PKR levels in sorted populations of HuAR2T.rKSHV.219 cells transduced with LV RTA or control LV BFP by immunoblot analysis (Figure 4B). Our data confirmed the marked depletion of PKR from the KSHV-activated RFP+ populations at 48 or 60 h post-RTA transduction compared with non-infected or latently infected cells. Furthermore, ectopic hemagglutinin (HA)-tagged PKR, but not a GFP control, was downregulated by lytic KSHV (Figure 4C), indicating that the effect is both specific and promoter independent. Phosphonoacetic acid (PAA) is a viral DNA polymerase inhibitor that blocks late gene expression (Sun

et al., 1999). PAA treatment of lytic (RFP+) cells did not affect depletion of PKR but did block expression of the late K8.1A/B proteins (Figure 4D), indicating that downregulation of PKR is an early lytic event. qRT-PCR analysis comparing mRNA from RFP+-sorted (lytic) and control (latent) cells showed that lytic KSHV reduces PKR gene expression by 4.7-fold (2.2-fold on the log2 scale) (Figure 4E). We also observed downregulation of LIMD1 (7.1-fold, or 2.8-fold on the log2 scale), a target of SOX endonuclease (Gaglia et al., 2015). Collectively, these results show that KSHV infection leads to a transcriptional downregulation of PKR, which is caused by an early lytic viral factor, in a PKR promoter-independent manner. Although we cannot exclude the possibility that PKR is affected by gene expression shutoff in lytic infection (Glaunsinger and Ganem, 2004b), we found that when expressed alone, the viral SOX protein did not affect PKR protein levels (Figure S3A). In addition to SOX, we tested several early lytic viral ORFs for PKR depletion, including ORF57, which is reported to interfere with PKR function (Sharma et al., 2017) (data not shown; Figure S3B). Despite extensive efforts, we were unable to identify the viral gene or genes responsible for PKR depletion, but we found that poly(I:C)-induced phosphorylation of eIF2α, a measure of downstream PKR signaling, was impaired upon KSHV reactivation (Figure 4F).

Proteins and Pathways Dysregulated by Lytic KSHV Reactivation in Endothelial Cells

Pairwise comparison of two independent proteomics experiments (from Figures 1D and 3A) showed that changes in protein abundancies correlate well between two datasets (Figure S4). To gain further insight into KSHV-induced changes in the host cell proteome, we performed a Gene Ontology (GO) term analysis of our proteomics datasets using the DAVID platform (Huang et al., 2009a, 2009b) (Table S4). Among upregulated proteins, marked enrichment of GO terms related to host cell machinery required for mRNA processing, translation, folding, and trafficking of proteins was seen (Figures 5A, S5A, and S5B). The most significantly enriched category was related to protein folding, mainly members of two families: (1) heat shock protein 70 (HSP70) and (2) all eight subunits of the chaperonin tailless complex polypeptide 1 (TCP1) ring complex (TRiC) (Figure 5B). Both families represent therapeutic targets for cancer treatment (Bassiouni et al., 2016; Boudesco et al., 2018; Carr et al., 2017). Poly(A) RNA binding was the most abundant category (74 proteins) of upregulated proteins (Figure 5C), and many members of this group are reported to associate with KSHV ORF57, the key player in viral mRNA processing (Majerciak and Zheng, 2015; Vogt and Bohne, 2016) (Table 1). The GO terms in the proteins downregulated by lytic KSHV were particularly enriched in surface receptors, protein kinases, and related biological processes, such as cell adhesion and signal transduction (Figures 5D and S5C–S5E). Although many of these proteins represent K5 targets (Figures 3C–3E), a substantial number of changes appear to be K5 independent, and lytic-phase KSHV infection readily validated the downregulation of three of the surface proteins (EphA2, ITGA6, and UFO kinase) by flow cytometry (Figures 5E and 5F). In addition to PKR (Figures 4A and 4B), in the category "protein kinase activity" (Figure 5G), we validated depletion of PKN2 kinase (Figure 5H). Proteins associated with the term "chromatin binding" were also enriched (Figure 5I). Several proteins from this group regulate lytic viral gene expression, e.g., HDACs and Rad21 (Gwack et al., 2001; Hu et al., 2019; Lu et al., 2003; Toth et al., 2017), as well as the nuclear factor κB (NF-κB) signaling pathway, which plays an important role in the switch from latent- to lytic-stage KSHV infection (de Oliveira et al., 2010), e.g., RelB and Ajuba proteins (Feng and Longmore, 2005). Lytic KSHV-mediated depletion of Ajuba was confirmed by immunoblot analysis of sorted lytic (RFP+) compared with latent control (BFP+) cells (Figure 5J). Collectively, these results emphasize the range of host proteins downregulated by lytic KSHV infection. Although the host cell machinery used by the virus is upregulated, the expression of cell-surface proteins, kinases, and chromatin binding proteins is predominantly downregulated.

Kinetic Profiling of the KSHV ORFs Reveals Viral Proteins Whose Expression Depends the Activity of Viral DNA Polymerase

To profile the temporal changes in viral and host protein abundance upon KSHV reactivation, we performed a proteomics analysis at three time points (36, 48, and 60 h) following reactivation of HuAR2T.rKSHV.219 Cas9 cells from latent to lytic infection (Figure 6A). Reliable proteomic data analysis for each time point requires FACS to enrich a homogeneous population of >85% lytic infected (RFP+) cells. The acquisition of a homogeneous population of lytic RFP+ cells earlier than 36 h postreactivation would have been desirable but was not technically feasible. To distinguish changes in the host cell proteome mediated by early and late viral proteins, we included samples in which KSHV reactivation was performed in the presence of PAA (at the 48 and 60 h time points), an inhibitor of viral DNA replication. We first sought to determine the expression kinetics of viral ORFs and performed a hierarchical cluster analysis of all KSHV proteins quantified in proteomics experiment 3. We generated temporal and PAA-sensitivity profiles for 62 KSHV ORFs (Figure 6B) and identified 5 clusters of viral proteins. Clusters 1 and 2 contain a total of 30 proteins, which increase in abundance from 36 to 60 h upon reactivation and are either insensitive (Figure 6C) or only mildly sensitive (Figure 6D) to PAA. Proteins from these clusters were previously reported to exhibit immediate-early expression kinetics, e.g., ORF45 or K8 (Zhu et al., 1999), or early expression kinetics, e.g., ORF61, K5, or ORF66 (Sun et al., 1999; Wang et al., 2010; Watanabe et al., 2020). The 15 proteins in cluster 3 also increase in abundance from 36 to 60 h but were more sensitive to PAA than those in cluster 2 (Figure 6E). Cluster 4 has 4 proteins that are strongly induced at 36 h and show moderate sensitivity to PAA treatment (Figure 6F). Intriguingly, two lytic viral gene products from this cluster, ORF37 and ORF49, were previously reported to show early kinetics (Glaunsinger and Ganem, 2004a; González et al., 2006; Lu et al., 2004). Cluster 5 contains 12 proteins whose expression increases in later infection (48–72 h) and that were markedly PAA sensitive (Figure 6G). Expression of many proteins from clusters 3 and 5 was previously reported to depend on viral DNA polymerase activity and exhibit late expression kinetics (Lu et al., 2004). These proteins are predominantly structural components of the KSHV virions, e.g., capsid proteins ORF17, ORF25,

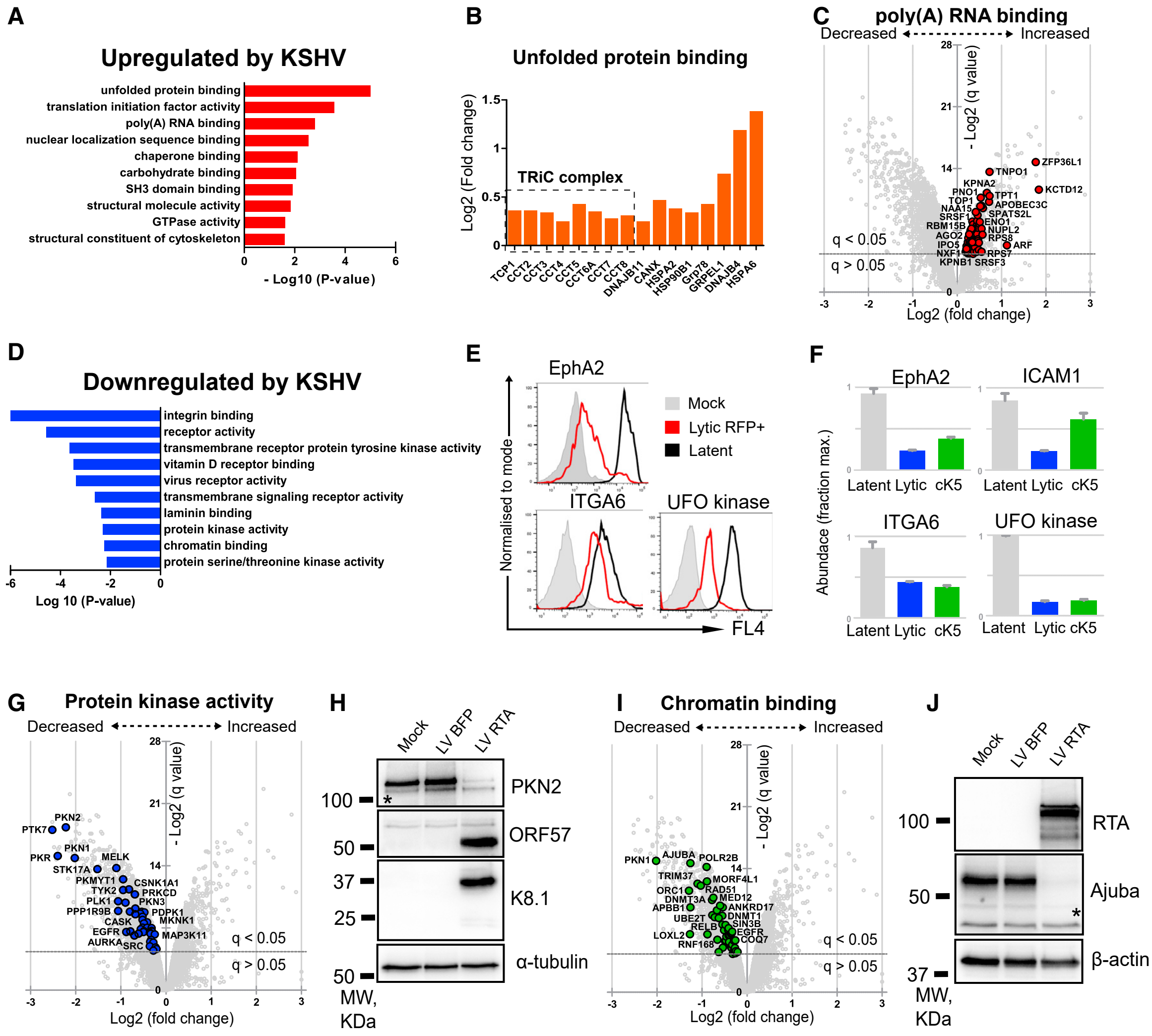

Figure 5. DAVID GO Term Analysis of Proteins Dysregulated by Lytic KSHV Infection

(A) Ten most enriched GO terms ranked by statistical significance (p value) in the category "molecular function" among proteins upregulated by lytic KSHV.

(B) Histogram shows the fold change in the abundance of the proteins from the GO term "unfolded protein binding."

(C) Upregulated proteins (red points) from the GO term "poly(A) RNA binding" are highlighted on the scatterplot that displays pairwise comparison between latent and lytic KSHV infections. Each point represents a single protein, plotted by its log2 (fold change in abundance) versus statistical significance (q value) of that change. Value was corrected for multiple hypothesis testing using the method of Benjamini-Hochberg. Dotted line: q = 0.05

(D) Ten most enriched GO terms ranked by statistical significance (p value) in the category "molecular function" among proteins downregulated by lytic KSHV.

(E) Validation of lytic KSHV-mediated downregulation of the surface proteins from the GO terms "integrin binding" and "transmembrane receptor protein tyrosine kinase activity."

(F) Relative abundance of the indicated host proteins in the cells with latent (gray), lytic CRISPR control (blue) and lytic CRISPR K5 (green) KSHV infections. Protein abundance is calculated as a fraction of the maximum TMT reporter ion intensity. Data are represented as mean ± SEM.

(G–J) Downregulated proteins from the GO terms "protein kinase activity" (G) and "chromatin binding" (I) are highlighted on the scatterplots that display pairwise comparison between latent and lytic KSHV infection. Each point represents a single protein, plotted by its log2 (fold change in abundance) versus statistical significance (q value) of that change. Values were corrected for multiple hypothesis testing using the method of Benjamini-Hochberg. Dotted line: q = 0.05. (H and J) Immunoblot analysis of HuAR2T.rKSHV.219 cells transduced with LV RTA or control LV BFP, sorted on BFP+ or RFP+, and probed with the indicated antibody. Non-specific bands are labeled with an asterisk.

See also Figure S5 and Table S4.

ORF26, ORF62, and ORF65; envelope glycoproteins gB (ORF8), gH (ORF22), ORF39 (gM), K8.1, and ORF28; and known or predicted tegument proteins ORF75, ORF64, ORF52, and ORF42 (Butnaru and Gaglia, 2019; Li et al., 2016; Zhu et al., 2005). In addition to structural proteins, highly PAA-sensitive KSHV gene products in cluster 5 include the putative portal protein ORF43 (Deng et al., 2007) and ORF20, a lytic protein which may promote KSHV infection through interaction with oligoadenylate synthetase-like protein (OASL) (Bussey et al., 2018). The expression of both ORFs has been previously shown to be sensitive to

Table 1. Upregulated Proteins Functionally Associated with KSHV ORF57

Protein	Alternative Name	Fold Change	Reference
SRSF1	SF2/ASF	1.24	Majerciak et al., 2008
SRSF3	SRp20	1.15	Majerciak et al., 2014
RBM15B	OTT3	1.27	Majerciak et al., 2011
NXF1	TAP	1.16	Malik et al., 2004
40S ribosomal subunits	–	1.25–148	Boyne et al., 2010
Ago2	EIF2C2	1.20	Sharma et al., 2019

inhibition of viral DNA polymerase activity (Dünn-Kittenplon et al., 2019; Lu et al., 2004); our results thus confirm these findings. A hierarchical cluster analysis of all host cell proteins quantified in the proteomics experiment, with temporal and PAA-dependency profiles for more than 8,000 host cell proteins, is shown (Figure S6A) and includes cellular proteins downregulated, e.g., PKR and PD-L2 (Figure S6B), or upregulated, e.g., ADAMTS1 and TPRC4, upon viral reactivation (Figure S6C). We therefore provide a unique time course of both KSHV and host cell protein expression following reactivation from latent- to lytic-phase infection and identify viral ORFs whose expression depends on viral DNA polymerase activity.

Proteomics Analysis of Cells with a Lytic CRISPR ORF57 Knockdown Mutant Reveals a Subset of KSHV ORFs that Requires ORF57 for Efficient Expression

ORF57 is a multifunctional KSHV protein that is known to stabilize viral transcripts, promote nuclear export, and enhance translation of several lytic viral gene products (Majerciak and Zheng, 2015). Although several studies previously focused on identification of KSHV transcripts and proteins whose expression depends on ORF57 (Majerciak et al., 2007; Verma et al., 2015; Vogt and Bohne, 2016), the complete set of KSHV proteins that require ORF57 for efficient expression remains unknown. To generate a KSHV CRISPR ORF57 mutant, we transduced HuAR2T.rKSHV.219 Cas9 cells with control or ORF57-specific sgRNAs and analyzed the ORF57 knockdown upon KSHV reactivation by immunoblot. The immunoblot analysis with ORF57-specific antibody shows efficient CRISPR-Cas9-mediated depletion of ORF57 in endothelial cells with lytic KSHV infection (Figure S7A). To compare changes induced by lytic CRISPR ORF57 and WT KSHV infection, we included sample of cells with lytic KSHV CRISPR ORF57 mutant infection, along with cells with control lytic KSHV infection, in proteomics experiment 3. The analysis of relative abundance of ORF57 in proteomics experiment 3 confirms efficient CRISPR-Cas9-mediated depletion of ORF57 (Figure S7B). To identify viral ORFs that require ORF57 for efficient expression, we performed hierarchical cluster analysis of relative protein abundancies of viral ORFs in lytic control versus CRISPR ORF57 infection (Figure S7C). We observe three groups of viral proteins in which (1) expression of KSHV ORFs is strongly inhibited by ORF57 knockdown, (2) expression is moderately inhibited by ORF57 knockdown, and (3) expression is independent of or enhanced by ORF57 knockdown. Thus, our data on KSHV gene products that require ORF57 for efficient expression confirm the results of previous studies (Majerciak et al., 2007; Verma et al., 2015; Vogt and Bohne, 2016) and extend the list of ORF57-dependent viral gene products.

DISCUSSION

In this study, we established a proteomics-compatible system to study KSHV reactivation in latently infected human endothelial cells. This allowed us to ascertain how lytic-stage KSHV infection alters both the viral and the host cell proteome. We provide a comprehensive description of changes in >7,000 viral and cellular proteins during lytic-phase KSHV infection, with a readily searchable interactive datasheet (Table S1). Our data show that KSHV uses both transcriptional and posttranscriptional mechanisms to remodel its host cell, with upregulation of cellular proteins related to mRNA biogenesis and protein production and downregulation of cell-surface receptors, adhesion molecules, protein kinases, and chromatin binding proteins. Despite the multiple KSHV episomes per cell, we show that CRISPR-Cas9 is an efficient mechanism for generating viral knockouts of lytic KSHV genes. By generating a CRISPR library of 1,281 KSHV-encoded sgRNAs, we performed KSHV genome-wide genetic screens that identified K5 as the viral gene responsible for the downregulation of cellular KSHV targets, two ligands of the DNAM-1-activating NK cell receptor. Although K5 emerges as the dominant KSHV-encoded viral gene for downregulating host cell proteins, the antiviral protein PKR was one of the top hits downregulated in a K5-independent manner. PKR was depleted by an early lytic viral factor with kinetics similar to inhibition of eIF2α phosphorylation, suggesting that PKR depletion may contribute to inhibition of translational shutoff mediated by lytic KSHV infection.

Previously, lytic KSHV-induced changes in host cells were analyzed at the mRNA level and revealed global inhibition of host gene expression, attributed to the viral SOX protein (ORF37) (Chandriani and Ganem, 2007; Glaunsinger and Ganem, 2004a, 2004b). Most (~95%) host transcripts were downregulated, with only a small subset (~2%) upregulated in lytic infection. How this gene expression shutoff correlates with changes in the proteome was unclear. Here we report the first comprehensive study of lytic KSHV-induced changes to the proteome of endothelial cells. Our results show that lytic KSHV significantly ($q < 0.05$) downregulates ~41% of host cell proteins. Somewhat surprisingly, ~15% of host cell proteins are significantly upregulated by lytic infection. This discrepancy may reflect the poor correlation between transcriptomics and proteomics data (reviewed in Haider and Pal, 2013) because of the involvement of additional factors such as efficiency of mRNA translation and protein stability.

Our analysis of proteins induced by KSHV reactivation identified enrichment for host cell components involved in mRNA processing, translation, and protein folding. This is not unexpected, because in the absence of encoding its own genes for these

CellPress
OPEN ACCESS

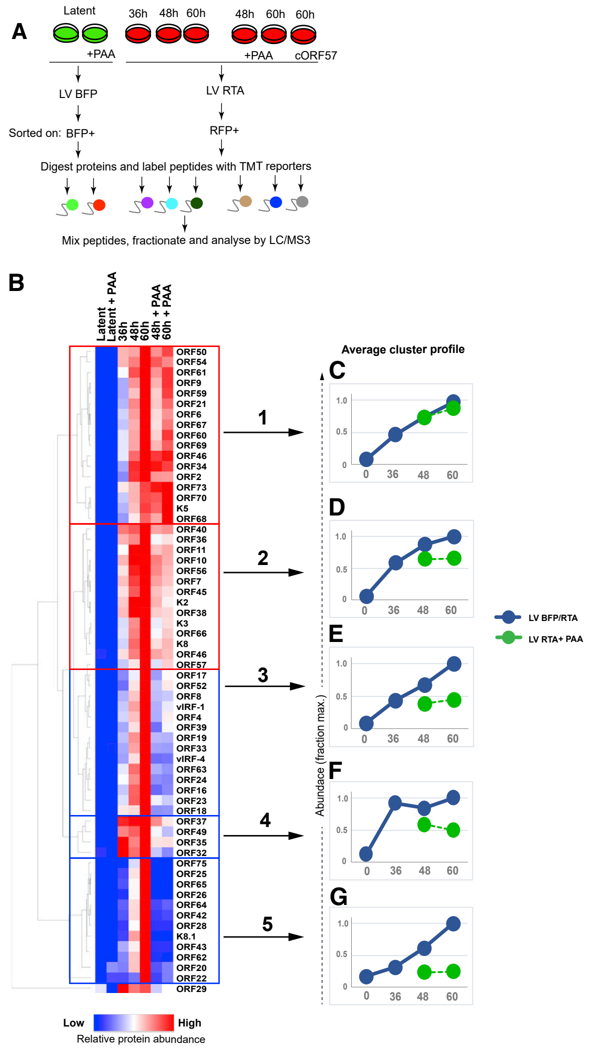

Figure 6. Kinetic Profiling of KSHV Proteins

(A) Schematic overview of the proteomic analysis of cells with latent versus lytic infection at 36, 48, and 60 h upon KSHV reactivation.

(B) Hierarchical cluster analysis of all viral proteins quantified. The heatmap diagram shows temporal and PAA-sensitivity profiles for 62 KSHV ORFs. Rows and columns represent individual viral ORFs and experimental samples, respectively.

(C–G) Average temporal and PAA-sensitivity profiles of viral proteins in clusters 1–5. Protein abundance is calculated as a fraction of maximum TMT reporter ion intensity.

See also Figures S6 and S7.

processes, KSHV must appropriate host cell machinery to enable successful virus replication. Among the upregulated mRNA binding proteins were several known KSHV ORF57 interaction partners, consistent with previous reports demonstrating a central role for this protein in the processing of KSHV RNAs (Boyne et al., 2010; Majerciak et al., 2011, 2014; Sharma et al., 2019). Upregulation of initiation factors supports previous findings on modulation of translation machinery by lytic KSHV (Arias et al., 2009; Kuang et al., 2011). The finding that KSHV induces components of Hsp70 and the TRiC complex families is important, because these may represent therapeutic targets (Bassiouni et al., 2016; Boudesco et al., 2018; Carr et al., 2017). Although their role in lytic KSHV infection is unknown, two Hsp70 isoforms are essential for lytic KSHV infection (Baquero-Pérez and Whitehouse, 2015).

CRISPR-Cas9 proved an effective means of depleting individual viral gene products from KSHV genomes in endothelial cells. CRISPR has recently been successfully used in other human herpesvirus infections: HSV-1 (Suenaga et al., 2014), HCMV (King and Munger, 2019), and EBV (van Diemen et al., 2016). During preparation of this manuscript, the CRISPR-mediated modification of KSHV ORF57 from PEL cell lines was also reported (BeltCappellino et al., 2019). The main challenge of using CRISPR in KSHV infection is that multiple viral genomes need to be targeted within the same cell, reducing the likelihood of generating a complete viral gene knockout. Here we initially found that CRISPR provided an effective means of targeting the KSHV genome for the four viral genes tested. The success of this technology paved the way for generating a sgRNA CRISPR library of KSHV-encoded genes to screen for viral genes responsible for the cellular phenotype of interest, in this case, the KSHV-mediated downregulation of CD155 and Nectin-2. Using this approach, we identified K5 as the viral gene responsible for the downregulation of both receptors, proving the utility of this screen in assigning cellular phenotypes to viral genes. Although our genetic screen with the KSHV CRISPR library identified K5 as a single clear hit, there are limitations to the use of this technology. In particular, the screen will not be successful if the phenotype of interest is mediated by a viral gene product essential for KSHV reactivation or if there is redundancy, i.e., if multiple viral gene products are responsible for the cellular phenotype.

A combination of CRISPR-based genetics and subsequent proteomics allowed us to identify endothelial cell proteins regulated by the KSHV K5 gene product. Using TMT-based proteomics to compare WT KSHV with CRISPR-mediated K5-deficient KSHV, we identified endothelial cell targets of K5. Two previous studies reported the cellular targets downregulated following ectopic K5 expression in both HeLa and KBM7 cells (Bartee et al., 2006; Timms et al., 2013). Our current approach identified 48 K5 targets in physiologically relevant endothelial cells and has the added advantage of being performed in the context of KSHV infection, as opposed to ectopic K5 viral gene expression. An interesting finding was the K5-dependent downregulation of Nectin-2 and CD155, ligands for the DNAM-1-activating NK cell receptor (de Andrade et al., 2014). Although CD155 is known to be downregulated by virus reactivation (Madrid and Ganem, 2012), this is the first report of Nectin-2's depletion by lytic KSHV. K5 protects virus-infected cells from NK cell lysis through depletion of ICAM-1 (Ishido et al., 2000a), NKG2D ligands MICA and MICB, and NKp80 ligand AICL (Thomas et al., 2008a). A role for DNAM-1-mediated control of herpesvirus infections by NK cells was previously demonstrated for the HCMV-encoded UL141 protein (Hsu et al., 2015; Prod'homme et al., 2010; Tomasec et al., 2005), MCMV m20.1 (Lenac Rovis et al., 2016), and HSV-2-encoded gD protein (Grauwet et al., 2014; De Pelsmaeker et al., 2018). Another endothelial cell K5 target involved in the NK cell response is TRAIL-R2, a receptor for the TNFSF10/TRAIL ligand (Walczak et al., 1997). TRAIL is

secreted by many immune cells, including NK cells, resulting in target cell killing (Cummins and Badley, 2009). Interestingly, TRAIL-R2, along with Nectin-2 and CD155, is downregulated by the HCMV UL141 protein for protection of HCMV-infected cells from TRAIL-mediated NK cell lysis (Smith et al., 2013). Altogether, these findings extend the repertoire of immune receptors targeted by KSHV and emphasize the central role of K5 in KSHV evasion from host cell immune response. The depletion of EphA2 by K5 was particularly significant, because it is an entry receptor for KSHV (Chakraborty et al., 2012; Hahn et al., 2012). Several viruses downregulate their entry receptor to prevent dual infection and enable viral release. This strategy may be particularly important for KSHV, because its gL/gH glycoprotein envelope complex has a high affinity for the EphA2 receptor (Großkopf et al., 2018). The loss of EphA2 would enable efficient virion release from the Golgi-derived vesicles during KSHV egress in a manner similar to that reported for tetherin, another K5 target (Mansouri et al., 2009; Pardieu et al., 2010).

In addition to immune receptors, K5 targets proteins whose downregulation has less clear functional consequences. A previous report identified K5-mediated depletion of STX4, a member of the SNARE family (Bartee et al., 2006), and we now identify additional SNARE members STX7, STX12, and VAMP8 as K5 targets. Although the reason for their depletion by K5 is unclear, many members of the SNARE family are involved in cytokine (Collins et al., 2015; Naegelen et al., 2015; Pagan et al., 2003) and tumor necrosis factor alpha (TNF-α) secretion (STX7) (Murray et al., 2005). KSHV K5-mediated STX7 downregulation may suppress cytokine release, because TNF-α inhibits KSHV gene transcription in endothelial cells (Milligan et al., 2004).

An intriguing finding was that lytic KSHV infection depleted host endothelial cells of PKR. Although all *Herpesviridae* family members interfere with PKR function, none have been shown to deplete cells of PKR. KSHV ORF57 is reported to interact with PKR and inhibit dsRNA binding and PKR auto-phosphorylation, leading to inhibition of eIF2α phosphorylation (Sharma et al., 2017). Our results imply that depletion of PKR must also contribute to the lytic KSHV-mediated block of translational shutoff. An outstanding question is, which viral gene or genes downregulate PKR? Its depletion was insensitive to PAA, suggesting involvement of an early viral function. Our attempts to screen using individual early viral ORF cDNAs or a KSHV CRISPR library screen for PKR depletion failed to identify significant hits (data not shown), suggesting PKR is likely to be targeted by more than one viral factor. Our results from two independent proteomics experiments show that at the protein level, lytic KSHV reactivation results in 3.5-fold and 5.3-fold downregulation of PKR. This is comparable to the qPCR analysis, which shows that lytic KSHV infection downregulates PKR mRNA 4.7-fold, but it remains to be determined whether decreased PKR transcription is the sole contributor to the PKR depletion observed at the protein level. Transcriptional downregulation of PKR, along with the observation that its depletion is promoter independent, suggests that PKR may be targeted by KSHV-encoded miRNAs. However, expression of most KSHV mRNAs is not enhanced in lytic-phase KSHV infection (Cai et al., 2005) and the activity of Drosha is inhibited in lytic-stage KSHV infection (Lin and Sullivan, 2011), making this less likely. ORF37 (SOX)-mediated shutoff of host gene expression (Glaunsinger and Ganem, 2004b) is likely to contribute to PKR suppression, but not to be the sole contributing factor. Isolated expression of SOX did not affect PKR protein level, suggesting additional viral genes are involved.

Our proteomic analysis identified 71 canonical viral proteins (>80% of KSHV proteome) but only two alternative translation products (KSHV ORF54A and K3A) compared with the 63 alternative translation viral products reported from ribosome profiling in lytic KSHV infection (Arias et al., 2014). Most non-canonical products are derived from alternate start codons as opposed to representing novel ORFs, making their differentiation from canonical ORFs challenging and dependent on the detection of a small number of unique peptides at the translational start site.

Our time course of protein expression upon viral reactivation identified 62 KSHV proteins, including those viral proteins whose expression depends on the viral DNA polymerase and ORF57 protein, together with more than 8,000 endothelial host cell proteins. A transcriptional kinetics analysis of lytic KSHV infection was previously reported in B cells (Lu et al., 2004; Paulose-Murphy et al., 2001) and endothelial cells (Yoo et al., 2005), but this is the first proteomics kinetics analysis of lytic KSHV infection. Furthermore, PAA treatment allowed us to distinguish KSHV proteins whose expression depends on viral DNA polymerase activity. Expression of 12/62 proteins (~19%) was highly PAA sensitive, 33 proteins (~53%) show intermediate PAA sensitivity, and 17 proteins (~27%) were PAA insensitive. These results correlate with microarray-based analysis of KSHV ORF expression with cidofovir (Lu et al., 2004). Lytic gene products reported to show early mRNA kinetics (ORF37 and ORF49) (Lu et al., 2004; Yoo et al., 2005) were PAA sensitive at the protein level. This discrepancy might be explained by distinct posttranscriptional regulation, i.e., mediated by machinery that is sensitive to PAA-mediated block of viral DNA polymerase.

In summary, we show how lytic-stage KSHV infection remodels the host cell proteome. Our results highlight the key host cell processes modulated by the virus during reactivation and extend our insight into how KSHV counteracts the host innate immune response. Our data also provide two important resources: (1) information on how host cell proteins are affected by lytic KSHV in endothelial cells and (2) sgRNA library sequences specific for the entire set of KSHV-encoded ORFs and miRNAs to generate CRISPR knockout KSHV mutants. Further work will be required to identify viral factors responsible for dysregulation of the host proteins identified in this study and to elucidate their role in KSHV pathogenesis.

STAR★METHODS

Detailed methods are provided in the online version of this paper and include the following:

- KEY RESOURCES TABLE
- RESOURCE AVAILABILITY
  - Lead Contact
  - Materials availability
  - Data and code availability
- EXPERIMENTAL MODEL AND SUBJECT DETAILS

- METHOD DETAILS
  - Plasmids construction
  - Lentivirus production and transduction
  - KSHV reactivation for proteomic analysis
  - Digestion and TMT Labeling
  - High pH Reverse Phase Fractionation
  - Mass spectrometry
  - CRISPR Cas9-mediated disruption of KSHV ORFs
  - Validation of cellular targets of KSHV K5 protein
  - Flow cytometry analysis
  - Immunoblot analysis
  - qRT-PCR analysis
  - KSHV CRISPR library design and cloning
  - Genetic screens with KSHV CRISPR sgRNA library
- QUANTIFICATION AND STATISTICAL ANALYSIS
  - Proteomic data processing and analysis
  - Gene Ontology analysis

SUPPLEMENTAL INFORMATION

Supplemental Information can be found online at https://doi.org/10.1016/j.celrep.2020.108249.

ACKNOWLEDGMENTS

The authors thank Dr. Reiner Schulte and the CIMR Flow Cytometry Core Facility team, David Lukac, Klaus Frueh, Britt Glaunsinger, Jens Bohne, and Su-Fang Lin for reagents and members of the Lehner and Matheson laboratories for critical discussions. This work was supported by the Medical Research Council (grant MR/R001405/1) and the NIHR Cambridge BRC. I.G. was supported by a Marie Skłodowska-Curie Individual Fellowship from the European Union's Framework Programme for Research and Innovation Horizon 2020 (grant 656511). T.F.S. was funded by the Deutsche Forschungsgemeinschaft (DFG, German Research Foundation) (SFB900/3-158989968), project C1.

AUTHOR CONTRIBUTIONS

I.G., T.F.S., and P.J.L. conceived the study. I.G. performed experiments and analyzed the data. J.C.W. performed experiments and analyzed the data. T.W.M.C. and P.J.L. analyzed the data. T.F.S. contributed essential reagents. I.G. and P.J.L. wrote the manuscript.

DECLARATION OF INTERESTS

The authors declare no competing interests.

Received: February 3, 2020
Revised: July 13, 2020
Accepted: September 17, 2020
Published: October 13, 2020

REFERENCES

Alkharsah, K.R., Singh, V.V., Bosco, R., Santag, S., Grundhoff, A., Konrad, A., Stürzl, M., Wirth, D., Dittrich-Breiholz, O., Kracht, M., and Schulz, T.F. (2011). Deletion of Kaposi's sarcoma-associated herpesvirus FLICE inhibitory protein, vFLIP, from the viral genome compromises the activation of STAT1-responsive cellular genes and spindle cell formation in endothelial cells. J. Virol. *85*, 10375–10388.

Aneja, K.K., and Yuan, Y. (2017). Reactivation and Lytic Replication of Kaposi's Sarcoma-Associated Herpesvirus: An Update. Front. Microbiol. *8*, 613.

Arias, C., Walsh, D., Harbell, J., Wilson, A.C., and Mohr, I. (2009). Activation of host translational control pathways by a viral developmental switch. PLoS Pathog. *5*, e1000334.

Arias, C., Weisburd, B., Stern-Ginossar, N., Mercier, A., Madrid, A.S., Bellare, P., Holdorf, M., Weissman, J.S., and Ganem, D. (2014). KSHV 2.0: a comprehensive annotation of the Kaposi's sarcoma-associated herpesvirus genome using next-generation sequencing reveals novel genomic and functional features. PLoS Pathog. *10*, e1003847.

Ashizawa, A., Higashi, C., Masuda, K., Ohga, R., Taira, T., and Fujimuro, M. (2012). The Ubiquitin System and Kaposi's Sarcoma-Associated Herpesvirus. Front. Microbiol. *3*, 66.

Baquero-Pérez, B., and Whitehouse, A. (2015). Hsp70 Isoforms Are Essential for the Formation of Kaposi's Sarcoma-Associated Herpesvirus Replication and Transcription Compartments. PLoS Pathog. *11*, e1005274.

Bartee, E., McCormack, A., and Früh, K. (2006). Quantitative membrane proteomics reveals new cellular targets of viral immune modulators. PLoS Pathog. *2*, e107.

Bassiouni, R., Nemec, K.N., Iketani, A., Flores, O., Showalter, A., Khaled, A.S., Vishnubhotla, P., Sprung, R.W., Jr., Kaittanis, C., Perez, J.M., and Khaled, A.R. (2016). Chaperonin Containing TCP-1 Protein Level in Breast Cancer Cells Predicts Therapeutic Application of a Cytotoxic Peptide. Clin. Cancer Res. *22*, 4366–4379.

Beisser, P.S., Verzijl, D., Gruijthuijsen, Y.K., Beuken, E., Smit, M.J., Leurs, R., Bruggeman, C.A., and Vink, C. (2005). The Epstein-Barr virus BILF1 gene encodes a G protein-coupled receptor that inhibits phosphorylation of RNA-dependent protein kinase. J. Virol. *79*, 441–449.

BeltCappellino, A., Majerciak, V., Lobanov, A., Lack, J., Cam, M., and Zheng, Z.-M. (2019). CRISPR/Cas9-Mediated Knockout and *In Situ* Inversion of the ORF57 Gene from All Copies of the Kaposi's Sarcoma-Associated Herpesvirus Genome in BCBL-1 Cells. J. Virol. *93*, e00628-19.

Boname, J.M., and Lehner, P.J. (2011). What has the study of the K3 and K5 viral ubiquitin E3 ligases taught us about ubiquitin-mediated receptor regulation? Viruses *3*, 118–131.

Bottino, C., Castriconi, R., Pende, D., Rivera, P., Nanni, M., Carnemolla, B., Cantoni, C., Grassi, J., Marcenaro, S., Reymond, N., et al. (2003). Identification of PVR (CD155) and Nectin-2 (CD112) as cell surface ligands for the human DNAM-1 (CD226) activating molecule. J. Exp. Med. *198*, 557–567.

Boudesco, C., Cause, S., Jego, G., and Garrido, C. (2018). Hsp70: A cancer target inside and outside the cell. Methods Mol. Biol. *1709*, 371–396.

Boyne, J.R., Jackson, B.R., Taylor, A., Macnab, S.A., and Whitehouse, A. (2010). Kaposi's sarcoma-associated herpesvirus ORF57 protein interacts with PYM to enhance translation of viral intronless mRNAs. EMBO J. *29*, 1851–1864.

Bu, W., Palmeri, D., Krishnan, R., Marin, R., Aris, V.M., Soteropoulos, P., and Lukac, D.M. (2008). Identification of direct transcriptional targets of the Kaposi's sarcoma-associated herpesvirus Rta lytic switch protein by conditional nuclear localization. J. Virol. *82*, 10709–10723.

Budt, M., Niederstadt, L., Valchanova, R.S., Jonjić, S., and Brune, W. (2009). Specific inhibition of the PKR-mediated antiviral response by the murine cytomegalovirus proteins m142 and m143. J. Virol. *83*, 1260–1270.

Burr, M.L., Sparbier, C.E., Chan, Y.C., Williamson, J.C., Woods, K., Beavis, P.A., Lam, E.Y.N., Henderson, M.A., Bell, C.C., Stolzenburg, S., et al. (2017). CMTM6 maintains the expression of PD-L1 and regulates anti-tumour immunity. Nature *549*, 101–105.

Burýsek, L., and Pitha, P.M. (2001). Latently expressed human herpesvirus 8-encoded interferon regulatory factor 2 inhibits double-stranded RNA-activated protein kinase. J. Virol. *75*, 2345–2352.

Bussey, K.A., Lau, U., Schumann, S., Gallo, A., Osbelt, L., Stempel, M., Arnold, C., Wissing, J., Gad, H.H., Hartmann, R., et al. (2018). The interferon-stimulated gene product oligoadenylate synthetase-like protein enhances replication of Kaposi's sarcoma-associated herpesvirus (KSHV) and interacts with the KSHV ORF20 protein. PLoS Pathog. *14*, e1006937.

Butnaru, M., and Gaglia, M.M. (2019). The Kaposi's Sarcoma-Associated Herpesvirus Protein ORF42 Is Required for Efficient Virion Production and Expression of Viral Proteins. Viruses *11*, 711.

Cai, X., Lu, S., Zhang, Z., Gonzalez, C.M., Damania, B., and Cullen, B.R. (2005). Kaposi's sarcoma-associated herpesvirus expresses an array of viral microRNAs in latently infected cells. Proc. Natl. Acad. Sci. USA *102*, 5570–5575.

Carr, A.C., Khaled, A.S., Bassiouni, R., Flores, O., Nierenberg, D., Bhatti, H., Vishnubhotla, P., Manuel, J.P., Santra, S., and Khaled, A.R. (2017). Targeting chaperonin containing TCP1 (CCT) as a molecular therapeutic for small cell lung cancer. Oncotarget *8*, 110273–110288.

Cesarman, E., Moore, P.S., Rao, P.H., Inghirami, G., Knowles, D.M., and Chang, Y. (1995). *In vitro* establishment and characterization of two acquired immunodeficiency syndrome-related lymphoma cell lines (BC-1 and BC-2) containing Kaposi's sarcoma-associated herpesvirus-like (KSHV) DNA sequences. Blood *86*, 2708–2714.

Chakraborty, S., Veettil, M.V., Bottero, V., and Chandran, B. (2012). Kaposi's sarcoma-associated herpesvirus interacts with EphrinA2 receptor to amplify signaling essential for productive infection. Proc. Natl. Acad. Sci. USA *109*, E1163–E1172.

Chandriani, S., and Ganem, D. (2007). Host transcript accumulation during lytic KSHV infection reveals several classes of host responses. PLoS ONE *2*, e811.

Chandriani, S., Xu, Y., and Ganem, D. (2010). The lytic transcriptome of Kaposi's sarcoma-associated herpesvirus reveals extensive transcription of noncoding regions, including regions antisense to important genes. J. Virol. *84*, 7934–7942.

Chen, Y., Ciustea, M., and Ricciardi, R.P. (2005). Processivity factor of KSHV contains a nuclear localization signal and binding domains for transporting viral DNA polymerase into the nucleus. Virology *340*, 183–191.

Chen, C.P., Lyu, Y., Chuang, F., Nakano, K., Izumiya, C., Jin, D., Campbell, M., and Izumiya, Y. (2017). Kaposi's Sarcoma-Associated Herpesvirus Hijacks RNA Polymerase II To Create a Viral Transcriptional Factory. J. Virol. *91*, e02491-16.

Cho, S.W., Kim, S., Kim, Y., Kweon, J., Kim, H.S., Bae, S., and Kim, J.S. (2014). Analysis of off-target effects of CRISPR/Cas-derived RNA-guided endonucleases and nickases. Genome Res. *24*, 132–141.

Chudasama, P., Konrad, A., Jochmann, R., Lausen, B., Holz, P., Naschberger, E., Neipel, F., Britzen-Laurent, N., and Stürzl, M. (2015). Structural proteins of Kaposi's sarcoma-associated herpesvirus antagonize p53-mediated apoptosis. Oncogene *34*, 639–649.

Collins, L.E., DeCourcey, J., Rochfort, K.D., Kristek, M., and Loscher, C.E. (2015). A role for syntaxin 3 in the secretion of IL-6 from dendritic cells following activation of toll-like receptors. Front. Immunol. *5*, 691.

Coscoy, L., and Ganem, D. (2000). Kaposi's sarcoma-associated herpesvirus encodes two proteins that block cell surface display of MHC class I chains by enhancing their endocytosis. Proc. Natl. Acad. Sci. USA *97*, 8051–8056.

Coscoy, L., and Ganem, D. (2001). A viral protein that selectively downregulates ICAM-1 and B7-2 and modulates T cell costimulation. J. Clin. Invest. *107*, 1599–1606.

Crameri, M., Bauer, M., Caduff, N., Walker, R., Steiner, F., Franzoso, F.D., Gujer, C., Boucke, K., Kucera, T., Zbinden, A., et al. (2018). MxB is an interferon-induced restriction factor of human herpesviruses. Nat. Commun. *9*, 1980.

Cummins, N., and Badley, A. (2009). The TRAIL to viral pathogenesis: the good, the bad and the ugly. Curr. Mol. Med. *9*, 495–505.

Damania, B., and Cesarman, E. (2013). Kaposi's Sarcoma-Associated Herpesvirus. In Fields Virology, D.M. Knipe and P. Howley, eds. (Lippincott Williams & Wilkins), pp. 2080–2128.

de Andrade, L.F., Smyth, M.J., and Martinet, L. (2014). DNAM-1 control of natural killer cells functions through nectin and nectin-like proteins. Immunol. Cell Biol. *92*, 237–244.

de Oliveira, D.E., Ballon, G., and Cesarman, E. (2010). NF-kappaB signaling modulation by EBV and KSHV. Trends Microbiol. *18*, 248–257.

De Pelsmaeker, S., Romero, N., Vitale, M., and Favoreel, H.W. (2018). Herpesvirus Evasion of Natural Killer Cells. J. Virol. *92*, e02105-17.

Deng, H., Young, A., and Sun, R. (2000). Auto-activation of the rta gene of human herpesvirus-8/Kaposi's sarcoma-associated herpesvirus. J. Gen. Virol. *81*, 3043–3048.

Deng, B., O'Connor, C.M., Kedes, D.H., and Zhou, Z.H. (2007). Direct visualization of the putative portal in the Kaposi's sarcoma-associated herpesvirus capsid by cryoelectron tomography. J. Virol. *81*, 3640–3644.

Di Bartolo, D., and Cesarman, E. (2004). Uncovering the complexities of Kaposi's sarcoma through genome-wide expression analysis. Genome Biol. *5*, 247.

Doench, J.G., Fusi, N., Sullender, M., Hegde, M., Vaimberg, E.W., Donovan, K.F., Smith, I., Tothova, Z., Wilen, C., Orchard, R., et al. (2016). Optimized sgRNA design to maximize activity and minimize off-target effects of CRISPR-Cas9. Nat. Biotechnol. *34*, 184–191.

Dresang, L.R., Teuton, J.R., Feng, H., Jacobs, J.M., Camp, D.G., 2nd, Purvine, S.O., Gritsenko, M.A., Li, Z., Smith, R.D., Sugden, B., et al. (2011). Coupled transcriptome and proteome analysis of human lymphotropic tumor viruses: insights on the detection and discovery of viral genes. BMC Genomics *12*, 625.

Dubich, T., Lieske, A., Santag, S., Beauclair, G., Rückert, J., Herrmann, J., Gorges, J., Büsche, G., Kazmaier, U., Hauser, H., et al. (2019). An endothelial cell line infected by Kaposi's sarcoma-associated herpes virus (KSHV) allows the investigation of Kaposi's sarcoma and the validation of novel viral inhibitors *in vitro* and *in vivo*. J. Mol. Med. (Berl.) *97*, 311–324.

Duncan, L.M., Piper, S., Dodd, R.B., Saville, M.K., Sanderson, C.M., Luzio, J.P., and Lehner, P.J. (2006). Lysine-63-linked ubiquitination is required for endolysosomal degradation of class I molecules. EMBO J. *25*, 1635–1645.

Dünn-Kittenplon, D.D., Kalt, I., Lellouche, J.-P.M., and Sarid, R. (2019). The KSHV portal protein ORF43 is essential for the production of infectious viral particles. Virology *529*, 205–215.

Feng, Y., and Longmore, G.D. (2005). The LIM protein Ajuba influences interleukin-1-induced NF-kappaB activation by affecting the assembly and activity of the protein kinase Czeta/p62/TRAF6 signaling complex. Mol. Cell. Biol. *25*, 4010–4022.

Gaglia, M.M., Rycroft, C.H., and Glaunsinger, B.A. (2015). Transcriptome-Wide Cleavage Site Mapping on Cellular mRNAs Reveals Features Underlying Sequence-Specific Cleavage by the Viral Ribonuclease SOX. PLoS Pathog. *11*, e1005305.

Ganem, D. (2010). KSHV and the pathogenesis of Kaposi sarcoma: listening to human biology and medicine. J. Clin. Invest. *120*, 939–949.

García, M.A., Meurs, E.F., and Esteban, M. (2007). The dsRNA protein kinase PKR: virus and cell control. Biochimie *89*, 799–811.

Glaunsinger, B.A. (2015). Modulation of the Translational Landscape During Herpesvirus Infection. Annu. Rev. Virol. *2*, 311–333.

Glaunsinger, B., and Ganem, D. (2004a). Highly selective escape from KSHV-mediated host mRNA shutoff and its implications for viral pathogenesis. J. Exp. Med. *200*, 391–398.

Glaunsinger, B., and Ganem, D. (2004b). Lytic KSHV infection inhibits host gene expression by accelerating global mRNA turnover. Mol. Cell *13*, 713–723.

González, C.M., Wong, E.L., Bowser, B.S., Hong, G.K., Kenney, S., and Damania, B. (2006). Identification and characterization of the Orf49 protein of Kaposi's sarcoma-associated herpesvirus. J. Virol. *80*, 3062–3070.

Goodman, D.E., Pretto, C.D., Krepostman, T.A., Carnahan, K.E., and Spindler, K.R. (2019). Enhanced Replication of Mouse Adenovirus Type 1 following Virus-Induced Degradation of Protein Kinase R (PKR). MBio *10*, e00668-19.

Gottwein, E. (2012). Kaposi's sarcoma-associated herpesvirus microRNAs. Front. Microbiol. *3*, 165.

Gramolelli, S., and Schulz, T.F. (2015). The role of Kaposi sarcoma-associated herpesvirus in the pathogenesis of Kaposi sarcoma. J. Pathol. *235*, 368–380.

Gramolelli, S., Elbasani, E., Tuohinto, K., Nurminen, V., Günther, T., Kallinen, R.E., Kaijalainen, S.P., Diaz, R., Grundhoff, A., Haglund, C., et al. (2020). Oncogenic Herpesvirus Engages Endothelial Transcription Factors SOX18 and PROX1 to Increase Viral Genome Copies and Virus Production. Cancer Res. *80*, 3116–3129.

Grauwet, K., Cantoni, C., Parodi, M., De Maria, A., Devriendt, B., Pende, D., Moretta, L., Vitale, M., and Favoreel, H.W. (2014). Modulation of CD112 by the alphaherpesvirus gD protein suppresses DNAM-1-dependent NK cell-mediated lysis of infected cells. Proc. Natl. Acad. Sci. USA *111*, 16118–16123.

Großkopf, A.K., Ensser, A., Neipel, F., Jungnickl, D., Schlagowski, S., Desrosiers, R.C., and Hahn, A.S. (2018). A conserved Eph family receptor-binding motif on the gH/gL complex of Kaposi's sarcoma-associated herpesvirus and rhesus monkey rhadinovirus. PLoS Pathog. *14*, e1006912.

Gwack, Y., Byun, H., Hwang, S., Lim, C., and Choe, J. (2001). CREB-binding protein and histone deacetylase regulate the transcriptional activity of Kaposi's sarcoma-associated herpesvirus open reading frame 50. J. Virol. *75*, 1909–1917.

Hahn, A.S., Kaufmann, J.K., Wies, E., Naschberger, E., Panteleev-Ivlev, J., Schmidt, K., Holzer, A., Schmidt, M., Chen, J., König, S., et al. (2012). The ephrin receptor tyrosine kinase A2 is a cellular receptor for Kaposi's sarcoma-associated herpesvirus. Nat. Med. *18*, 961–966.

Haider, S., and Pal, R. (2013). Integrated analysis of transcriptomic and proteomic data. Curr. Genomics *14*, 91–110.

Hakki, M., Marshall, E.E., De Niro, K.L., and Geballe, A.P. (2006). Binding and nuclear relocalization of protein kinase R by human cytomegalovirus TRS1. J. Virol. *80*, 11817–11826.

Hopcraft, S.E., Pattenden, S.G., James, L.I., Frye, S., Dittmer, D.P., and Damania, B. (2018). Chromatin remodeling controls Kaposi's sarcoma-associated herpesvirus reactivation from latency. PLoS Pathog. *14*, e1007267.

Hartenian, E., Gilbertson, S., Federspiel, J.D., Cristea, I.M., and Glaunsinger, B.A. (2020). RNA decay during gammaherpesvirus infection reduces RNA polymerase II occupancy of host promoters but spares viral promoters. PLoS Pathog, e1008269.

Hsu, P.D., Scott, D.A., Weinstein, J.A., Ran, F.A., Konermann, S., Agarwala, V., Li, Y., Fine, E.J., Wu, X., Shalem, O., et al. (2013). DNA targeting specificity of RNA-guided Cas9 nucleases. Nat. Biotechnol. *31*, 827–832.

Hsu, J.-L., van den Boomen, D.J.H., Tomasec, P., Weekes, M.P., Antrobus, R., Stanton, R.J., Ruckova, E., Sugrue, D., Wilkie, G.S., Davison, A.J., et al. (2015). Plasma membrane profiling defines an expanded class of cell surface proteins selectively targeted for degradation by HCMV US2 in cooperation with UL141. PLoS Pathog. *11*, e1004811.

Hu, M., Armstrong, N., Seto, E., Li, W., Zhu, F., Wang, P.C., and Tang, Q. (2019). Sirtuin 6 Attenuates Kaposi's Sarcoma-Associated Herpesvirus Reactivation by Suppressing Ori-Lyt Activity and Expression of RTA. J. Virol. *93*, 2200–2218.

Huang, W., Sherman, B.T., and Lempicki, R.A. (2009a). Bioinformatics enrichment tools: paths toward the comprehensive functional analysis of large gene lists. Nucleic Acids Res. *37*, 1–13.

Huang, D.W., Sherman, B.T., and Lempicki, R.A. (2009b). Systematic and integrative analysis of large gene lists using DAVID Bioinformatics Resources. Nat. Protoc. *4*, 44–57.

Huber, W., Carey, V.J., Gentleman, R., Anders, S., Carlson, M., Carvalho, B.S., Bravo, H.C., Davis, S., Gatto, L., Girke, T., et al. (2015). Orchestrating high-throughput genomic analysis with Bioconductor. Nat. Methods *12*, 115–121.

Inn, K.-S., Lee, S.-H., Rathbun, J.Y., Wong, L.-Y., Toth, Z., Machida, K., Ou, J.-H.J., and Jung, J.U. (2011). Inhibition of RIG-I-mediated signaling by Kaposi's sarcoma-associated herpesvirus-encoded deubiquitinase ORF64. J. Virol. *85*, 10899–10904.

Ishido, S., Choi, J.K., Lee, B.S., Wang, C., DeMaria, M., Johnson, R.P., Cohen, G.B., and Jung, J.U. (2000a). Inhibition of natural killer cell-mediated cytotoxicity by Kaposi's sarcoma-associated herpesvirus K5 protein. Immunity *13*, 365–374.

Ishido, S., Wang, C., Lee, B.S., Cohen, G.B., and Jung, J.U. (2000b). Downregulation of major histocompatibility complex class I molecules by Kaposi's sarcoma-associated herpesvirus K3 and K5 proteins. J. Virol. *74*, 5300–5309.

Jacquemont, B., and Roizman, B. (1975). RNA synthesis in cells infected with herpes simplex virus. X. Properties of viral symmetric transcripts and of double-stranded RNA prepared from them. J. Virol. *15*, 707–713.

Kalveram, B., and Ikegami, T. (2013). Toscana virus NSs protein promotes degradation of double-stranded RNA-dependent protein kinase. J. Virol. *87*, 3710–3718.

Katano, H., Sato, Y., Kurata, T., Mori, S., and Sata, T. (2000). Expression and localization of human herpesvirus 8-encoded proteins in primary effusion lymphoma, Kaposi's sarcoma, and multicentric Castleman's disease. Virology *269*, 335–344.

Kerur, N., Veettil, M.V., Sharma-Walia, N., Bottero, V., Sadagopan, S., Otageri, P., and Chandran, B. (2011). IFI16 acts as a nuclear pathogen sensor to induce the inflammasome in response to Kaposi Sarcoma-associated herpesvirus infection. Cell Host Microbe *9*, 363–375.

King, M.W., and Munger, J. (2019). Editing the human cytomegalovirus genome with the CRISPR/Cas9 system. Virology *529*, 186–194.

Ko, Y.C., Tsai, W.H., Wang, P.W., Wu, I.L., Lin, S.Y., Chen, Y.L., Chen, J.Y., and Lin, S.F. (2012). Suppressive regulation of KSHV RTA with O-GlcNAcylation. J. Biomed. Sci. *19*, 12.

König, R., Chiang, C.Y., Tu, B.P., Yan, S.F., DeJesus, P.D., Romero, A., Bergauer, T., Orth, A., Krueger, U., Zhou, Y., and Chanda, S.K. (2007). A probability-based approach for the analysis of large-scale RNAi screens. Nat. Methods *4*, 847–849.

Kuang, E., Fu, B., Liang, Q., Myoung, J., and Zhu, F. (2011). Phosphorylation of eukaryotic translation initiation factor 4B (EIF4B) by open reading frame 45/p90 ribosomal S6 kinase (ORF45/RSK) signaling axis facilitates protein translation during Kaposi sarcoma-associated herpesvirus (KSHV) lytic replication. J. Biol. Chem. *286*, 41171–41182.

Lan, K., Kuppers, D.A., Verma, S.C., and Robertson, E.S. (2004). Kaposi's sarcoma-associated herpesvirus-encoded latency-associated nuclear antigen inhibits lytic replication by targeting Rta: a potential mechanism for virus-mediated control of latency. J. Virol. *78*, 6585–6594.

Lan, K., Kuppers, D.A., and Robertson, E.S. (2005). Kaposi's sarcoma-associated herpesvirus reactivation is regulated by interaction of latency-associated nuclear antigen with recombination signal sequence-binding protein Jkappa, the major downstream effector of the Notch signaling pathway. J. Virol. *79*, 3468–3478.

Lenac Rovis, T., Kucan Brlic, P., Kaynan, N., Juranic Lisnic, V., Brizic, I., Jordan, S., Tomic, A., Kvestak, D., Babic, M., Tsukerman, P., et al. (2016). Inflammatory monocytes and NK cells play a crucial role in DNAM-1-dependent control of cytomegalovirus infection. J. Exp. Med. *213*, 1835–1850.

Li, D., and Swaminathan, S. (2019). Human IFIT proteins inhibit lytic replication of KSHV: A new feed-forward loop in the innate immune system. PLoS Pathog. *15*, e1007609.

Li, Y., Zhang, C., Chen, X., Yu, J., Wang, Y., Yang, Y., Du, M., Jin, H., Ma, Y., He, B., and Cao, Y. (2011). ICP34.5 protein of herpes simplex virus facilitates the initiation of protein translation by bridging eukaryotic initiation factor 2alpha (eIF2alpha) and protein phosphatase 1. J. Biol. Chem. *286*, 24785–24792.

Li, W., Avey, D., Fu, B., Wu, J.J., Ma, S., Liu, X., and Zhu, F. (2016). Kaposi's Sarcoma-Associated Herpesvirus Inhibitor of cGAS (KicGAS), Encoded by ORF52, Is an Abundant Tegument Protein and Is Required for Production of Infectious Progeny Viruses. J. Virol. *90*, 5329–5342.

Lin, Y.-T., and Sullivan, C.S. (2011). Expanding the role of Drosha to the regulation of viral gene expression. Proc. Natl. Acad. Sci. USA *108*, 11229–11234.

Lu, F., Zhou, J., Wiedmer, A., Madden, K., Yuan, Y., and Lieberman, P.M. (2003). Chromatin remodeling of the Kaposi's sarcoma-associated herpesvirus ORF50 promoter correlates with reactivation from latency. J. Virol. *77*, 11425–11435.

Lu, M., Suen, J., Frias, C., Pfeiffer, R., Tsai, M.-H., Chuang, E., and Zeichner, S.L. (2004). Dissection of the Kaposi's sarcoma-associated herpesvirus gene expression program by using the viral DNA replication inhibitor cidofovir. J. Virol. *78*, 13637–13652.

Lu, F., Day, L., Gao, S.-J., and Lieberman, P.M. (2006). Acetylation of the latency-associated nuclear antigen regulates repression of Kaposi's sarcoma-associated herpesvirus lytic transcription. J. Virol. *80*, 5273–5282.

Lukac, D.M., Renne, R., Kirshner, J.R., and Ganem, D. (1998). Reactivation of Kaposi's sarcoma-associated herpesvirus infection from latency by expression of the ORF 50 transactivator, a homolog of the EBV R protein. Virology *252*, 304–312.

Lukac, D.M., Kirshner, J.R., and Ganem, D. (1999). Transcriptional activation by the product of open reading frame 50 of Kaposi's sarcoma-associated herpesvirus is required for lytic viral reactivation in B cells. J. Virol. *73*, 9348–9361.

Ly, T., Whigham, A., Clarke, R., Brenes-Murillo, A.J., Estes, B., Madhessian, D., Lundberg, E., Wadsworth, P., and Lamond, A.I. (2017). Proteomic analysis of cell cycle progression in asynchronous cultures, including mitotic subphases, using PRIMMUS. eLife *6*, e27574.

Ma, Z., Jacobs, S.R., West, J.A., Stopford, C., Zhang, Z., Davis, Z., Barber, G.N., Glaunsinger, B.A., Dittmer, D.P., and Damania, B. (2015). Modulation of the cGAS-STING DNA sensing pathway by gammaherpesviruses. Proc. Natl. Acad. Sci. USA *112*, E4306–E4315.

Madrid, A.S., and Ganem, D. (2012). Kaposi's sarcoma-associated herpesvirus ORF54/dUTPase downregulates a ligand for the NK activating receptor NKp44. J. Virol. *86*, 8693–8704.

Majerciak, V., and Zheng, Z.M. (2015). KSHV ORF57, a protein of many faces. Viruses *7*, 604–633.

Majerciak, V., Pripuzova, N., McCoy, J.P., Gao, S.-J., and Zheng, Z.-M. (2007). Targeted disruption of Kaposi's sarcoma-associated herpesvirus ORF57 in the viral genome is detrimental for the expression of ORF59, K8α, and K8.1 and the production of infectious virus. J. Virol. *81*, 1062–1071.

Majerciak, V., Yamanegi, K., Allemand, E., Kruhlak, M., Krainer, A.R., and Zheng, Z.-M. (2008). Kaposi's sarcoma-associated herpesvirus ORF57 functions as a viral splicing factor and promotes expression of intron-containing viral lytic genes in spliceosome-mediated RNA splicing. J. Virol. *82*, 2792–2801.

Majerciak, V., Uranishi, H., Kruhlak, M., Pilkington, G.R., Massimelli, M.J., Bear, J., Pavlakis, G.N., Felber, B.K., and Zheng, Z.-M. (2011). Kaposi's sarcoma-associated herpesvirus ORF57 interacts with cellular RNA export cofactors RBM15 and OTT3 to promote expression of viral ORF59. J. Virol. *85*, 1528–1540.

Majerciak, V., Lu, M., Li, X., and Zheng, Z.M. (2014). Attenuation of the suppressive activity of cellular splicing factor SRSF3 by Kaposi sarcoma-associated herpesvirus ORF57 protein is required for RNA splicing. RNA *20*, 1747–1758.

Malik, P., Blackbourn, D.J., and Clements, J.B. (2004). The evolutionarily conserved Kaposi's sarcoma-associated herpesvirus ORF57 protein interacts with REF protein and acts as an RNA export factor. J. Biol. Chem. *279*, 33001–33011.

Manners, O., Murphy, J.C., Coleman, A., Hughes, D.J., and Whitehouse, A. (2018). Contribution of the KSHV and EBV lytic cycles to tumourigenesis. Curr. Opin. Virol. *32*, 60–70.

Mansouri, M., Douglas, J., Rose, P.P., Gouveia, K., Thomas, G., Means, R.E., Moses, A.V., and Früh, K. (2006). Kaposi sarcoma herpesvirus K5 removes CD31/PECAM from endothelial cells. Blood *108*, 1932–1940.

Mansouri, M., Viswanathan, K., Douglas, J.L., Hines, J., Gustin, J., Moses, A.V., and Früh, K. (2009). Molecular mechanism of BST2/tetherin downregulation by K5/MIR2 of Kaposi's sarcoma-associated herpesvirus. J. Virol. *83*, 9672–9681.

May, T., Butueva, M., Bantner, S., Markusic, D., Seppen, J., MacLeod, R.A.F., Weich, H., Hauser, H., and Wirth, D. (2010). Synthetic gene regulation circuits for control of cell expansion. Tissue Eng. Part A *16*, 441–452.

Milligan, S., Robinson, M., O'Donnell, E., and Blackbourn, D.J. (2004). Inflammatory cytokines inhibit Kaposi's sarcoma-associated herpesvirus lytic gene transcription in *in vitro*-infected endothelial cells. J. Virol. *78*, 2591–2596.

Mudhasani, R., Tran, J.P., Retterer, C., Kota, K.P., Whitehouse, C.A., and Bavari, S. (2016). Protein Kinase R Degradation Is Essential for Rift Valley Fever Virus Infection and Is Regulated by SKP1-CUL1-F-box (SCF)FBXW11-NSs E3 Ligase. PLoS Pathog. *12*, e1005437.

Murray, R.Z., Kay, J.G., Sangermani, D.G., and Stow, J.L. (2005). A role for the phagosome in cytokine secretion. Science *310*, 1492–1495.

Naegelen, I., Plançon, S., Nicot, N., Kaoma, T., Muller, A., Vallar, L., Tschirhart, E.J., and Bréchard, S. (2015). An essential role of syntaxin 3 protein for granule exocytosis and secretion of IL-1α, IL-1β, IL-12b, and CCL4 from differentiated HL-60 cells. J. Leukoc. Biol. *97*, 557–571.

Nishimura, M., Watanabe, T., Yagi, S., Yamanaka, T., and Fujimuro, M. (2017). Kaposi's sarcoma-associated herpesvirus ORF34 is essential for late gene expression and virus production. Sci. Rep. *7*, 329.

Ojala, P.M., and Schulz, T.F. (2014). Manipulation of endothelial cells by KSHV: implications for angiogenesis and aberrant vascular differentiation. Semin. Cancer Biol. *26*, 69–77.

Ozgur, S., and Griffith, J. (2014). Interaction of Kaposi's sarcoma-associated herpesvirus ORF6 protein with single-stranded DNA. J. Virol. *88*, 8687–8695.

Pagan, J.K., Wylie, F.G., Joseph, S., Widberg, C., Bryant, N.J., James, D.E., and Stow, J.L. (2003). The t-SNARE syntaxin 4 is regulated during macrophage activation to function in membrane traffic and cytokine secretion. Curr. Biol. *13*, 156–160.

Pardieu, C., Vigan, R., Wilson, S.J., Calvi, A., Zang, T., Bieniasz, P., Kellam, P., Towers, G.J., and Neil, S.J.D. (2010). The RING-CH ligase K5 antagonizes restriction of KSHV and HIV-1 particle release by mediating ubiquitin-dependent endosomal degradation of tetherin. PLoS Pathog. *6*, e1000843.

Paulose-Murphy, M., Ha, N.-K., Xiang, C., Chen, Y., Gillim, L., Yarchoan, R., Meltzer, P., Bittner, M., Trent, J., and Zeichner, S. (2001). Transcription program of human herpesvirus 8 (Kaposi's sarcoma-associated herpesvirus). J. Virol. *75*, 4843–4853.

Peng, C., Chen, J., Tang, W., Liu, C., and Chen, X. (2014). Kaposi's sarcoma-associated herpesvirus ORF6 gene is essential in viral lytic replication. PLoS ONE *9*, e99542.

Perez-Riverol, Y., Csordas, A., Bai, J., Bernal-Llinares, M., Hewapathirana, S., Kundu, D.J., Inuganti, A., Griss, J., Mayer, G., Eisenacher, M., et al. (2019). The PRIDE database and related tools and resources in 2019: improving support for quantification data. Nucleic Acids Res. *47* (*D1*), D442–D450.

Poppers, J., Mulvey, M., Khoo, D., and Mohr, I. (2000). Inhibition of PKR activation by the proline-rich RNA binding domain of the herpes simplex virus type 1 Us11 protein. J. Virol. *74*, 11215–11221.

Poppers, J., Mulvey, M., Perez, C., Khoo, D., and Mohr, I. (2003). Identification of a lytic-cycle Epstein-Barr virus gene product that can regulate PKR activation. J. Virol. *77*, 228–236.

Prod'homme, V., Sugrue, D.M., Stanton, R.J., Nomoto, A., Davies, J., Rickards, C.R., Cochrane, D., Moore, M., Wilkinson, G.W.G., and Tomasec, P. (2010). Human cytomegalovirus UL141 promotes efficient downregulation of the natural killer cell activating ligand CD112. J. Gen. Virol. *91*, 2034–2039.

Rhodes, D.A., Boyle, L.H., Boname, J.M., Lehner, P.J., and Trowsdale, J. (2010). Ubiquitination of lysine-331 by Kaposi's sarcoma-associated herpesvirus protein K5 targets HFE for lysosomal degradation. Proc. Natl. Acad. Sci. USA *107*, 16240–16245.

Roy, A., Dutta, D., Iqbal, J., Pisano, G., Gjyshi, O., Ansari, M.A., Kumar, B., and Chandran, B. (2016). Nuclear Innate Immune DNA Sensor IFI16 Is Degraded during Lytic Reactivation of Kaposi's Sarcoma-Associated Herpesvirus (KSHV): Role of IFI16 in Maintenance of KSHV Latency. J. Virol. *90*, 8822–8841.

Schulz, T.F. (2006). The pleiotropic effects of Kaposi's sarcoma herpesvirus. J. Pathol. *208*, 187–198.

Schwämmle, V., León, I.R., and Jensen, O.N. (2013). Assessment and improvement of statistical tools for comparative proteomics analysis of sparse data sets with few experimental replicates. J. Proteome Res. *12*, 3874–3883.

Sciortino, M.T., Parisi, T., Siracusano, G., Mastino, A., Taddeo, B., and Roizman, B. (2013). The virion host shutoff RNase plays a key role in blocking the activation of protein kinase R in cells infected with herpes simplex virus 1. J. Virol. *87*, 3271–3276.

Sharma, N.R., Majerciak, V., Kruhlak, M.J., and Zheng, Z.-M. (2017). KSHV inhibits stress granule formation by viral ORF57 blocking PKR activation. PLoS Pathog. *13*, e1006677.

Sharma, N.R., Majerciak, V., Kruhlak, M.J., Yu, L., Kang, J.G., Yang, A., Gu, S., Fritzler, M.J., and Zheng, Z.-M. (2019). KSHV RNA-binding protein ORF57 inhibits P-body formation to promote viral multiplication by interaction with Ago2 and GW182. Nucleic Acids Res. *47*, 9368–9385.

Smith, W., Tomasec, P., Aicheler, R., Loewendorf, A., Nemčovičová, I., Wang, E.C.Y., Stanton, R.J., Macauley, M., Norris, P., Willen, L., et al. (2013). Human cytomegalovirus glycoprotein UL141 targets the TRAIL death receptors to thwart host innate antiviral defenses. Cell Host Microbe *13*, 324–335.

Soulier, J., Grollet, L., Oksenhendler, E., Cacoub, P., Cazals-Hatem, D., Babinet, P., d'Agay, M.F., Clauvel, J.P., Raphael, M., Degos, L., et al. (1995). Kaposi's sarcoma-associated herpesvirus-like DNA sequences in multicentric Castleman's disease. Blood *86*, 1276–1280.

Stevenson, P.G., Efstathiou, S., Doherty, P.C., and Lehner, P.J. (2000). Inhibition of MHC class I-restricted antigen presentation by γ 2-herpesviruses. Proc. Natl. Acad. Sci. USA *97*, 8455–8460.

Suenaga, T., Kohyama, M., Hirayasu, K., and Arase, H. (2014). Engineering large viral DNA genomes using the CRISPR-Cas9 system. Microbiol. Immunol. *58*, 513–522.

Sun, R., Lin, S.F., Gradoville, L., Yuan, Y., Zhu, F., and Miller, G. (1998). A viral gene that activates lytic cycle expression of Kaposi's sarcoma-associated herpesvirus. Proc. Natl. Acad. Sci. USA *95*, 10866–10871.

Sun, R., Lin, S.-F., Staskus, K., Gradoville, L., Grogan, E., Haase, A., and Miller, G. (1999). Kinetics of Kaposi's sarcoma-associated herpesvirus gene expression. J. Virol. *73*, 2232–2242.

Sun, R., Liang, D., Gao, Y., and Lan, K. (2014). Kaposi's sarcoma-associated herpesvirus-encoded LANA interacts with host KAP1 to facilitate establishment of viral latency. J. Virol. *88*, 7331–7344.

Thomas, M., Boname, J.M., Field, S., Nejentsev, S., Salio, M., Cerundolo, V., Wills, M., and Lehner, P.J. (2008a). Down-regulation of NKG2D and NKp80 ligands by Kaposi's sarcoma-associated herpesvirus K5 protects against NK cell cytotoxicity. Proc. Natl. Acad. Sci. USA *105*, 1656–1661.

Thomas, M., Wills, M., and Lehner, P.J. (2008b). Natural killer cell evasion by an E3 ubiquitin ligase from Kaposi's sarcoma-associated herpesvirus. Biochem. Soc. Trans. *36*, 459–463.

Timms, R.T., Duncan, L.M., Tchasovnikarova, I.A., Antrobus, R., Smith, D.L., Dougan, G., Weekes, M.P., and Lehner, P.J. (2013). Haploid genetic screens identify an essential role for PLP2 in the downregulation of novel plasma membrane targets by viral E3 ubiquitin ligases. PLoS Pathog. *9*, e1003772.

Tomasec, P., Wang, E.C.Y., Davison, A.J., Vojtesek, B., Armstrong, M., Griffin, C., McSharry, B.P., Morris, R.J., Llewellyn-Lacey, S., Rickards, C., et al. (2005). Downregulation of natural killer cell-activating ligand CD155 by human cytomegalovirus UL141. Nat. Immunol. *6*, 181–188.

Toth, Z., Smindak, R.J., and Papp, B. (2017). Inhibition of the lytic cycle of Kaposi's sarcoma-associated herpesvirus by cohesin factors following *de novo* infection. Virology *512*, 25–33.

Uldrick, T.S., and Whitby, D. (2011). Update on KSHV epidemiology, Kaposi Sarcoma pathogenesis, and treatment of Kaposi Sarcoma. Cancer Lett. *305*, 150–162.

Valchanova, R.S., Picard-Maureau, M., Budt, M., and Brune, W. (2006). Murine cytomegalovirus m142 and m143 are both required to block protein kinase R-mediated shutdown of protein synthesis. J. Virol. *80*, 10181–10190.

van Diemen, F.R., Kruse, E.M., Hooykaas, M.J.G., Bruggeling, C.E., Schürch, A.C., van Ham, P.M., Imhof, S.M., Nijhuis, M., Wiertz, E.J.H.J., and Lebbink, R.J. (2016). CRISPR/Cas9-Mediated Genome Editing of Herpesviruses Limits Productive and Latent Infections. PLoS Pathog. *12*, e1005701.

Verma, D., Li, D.-J., Krueger, B., Renne, R., and Swaminathan, S. (2015). Identification of the physiological gene targets of the essential lytic replicative Kaposi's sarcoma-associated herpesvirus ORF57 protein. J. Virol. *89*, 1688–1702.

Vieira, J., and O'Hearn, P.M. (2004). Use of the red fluorescent protein as a marker of Kaposi's sarcoma-associated herpesvirus lytic gene expression. Virology *325*, 225–240.

Vogt, C., and Bohne, J. (2016). The KSHV RNA regulator ORF57: target specificity and its role in the viral life cycle. Wiley Interdiscip. Rev. RNA *7*, 173–185.

Vogt, C., Hackmann, C., Rabner, A., Koste, L., Santag, S., Kati, S., Mandel-Gutfreund, Y., Schulz, T.F., and Bohne, J. (2015). ORF57 overcomes the detrimental sequence bias of Kaposi's sarcoma-associated herpesvirus lytic genes. J. Virol. *89*, 5097–5109.

Walczak, H., Degli-Esposti, M.A., Johnson, R.S., Smolak, P.J., Waugh, J.Y., Boiani, N., Timour, M.S., Gerhart, M.J., Schooley, K.A., Smith, C.A., et al. (1997). TRAIL-R2: a novel apoptosis-mediating receptor for TRAIL. EMBO J. *16*, 5386–5397.

Wang, S.S., Chen, L.W., Chen, L.Y., Tsai, H.H., Shih, Y.C., Yang, C.T., and Chang, P.J. (2010). Transcriptional regulation of the ORF61 and ORF60 genes of Kaposi's sarcoma-associated herpesvirus. Virology *397*, 311–321.

Wang, T., Birsoy, K., Hughes, N.W., Krupczak, K.M., Post, Y., Wei, J.J., Lander, E.S., and Sabatini, D.M. (2015). Identification and characterization of essential genes in the human genome. Science *350*, 1096–1101.

Watanabe, T., Nishimura, M., Izumi, T., Kuriyama, K., Iwaisako, Y., Hosokawa, K., Takaori-Kondo, A., and Fujimuro, M. (2020). Kaposi's Sarcoma-Associated Herpesvirus ORF66 Is Essential for Late Gene Expression and Virus Production via Interaction with ORF34. J. Virol. *94*, e01300-19.

Weber, F., Wagner, V., Rasmussen, S.B., Hartmann, R., and Paludan, S.R. (2006). Double-stranded RNA is produced by positive-strand RNA viruses and DNA viruses but not in detectable amounts by negative-strand RNA viruses. J. Virol. *80*, 5059–5064.

West, J.A., Wicks, M., Gregory, S.M., Chugh, P., Jacobs, S.R., Zhang, Z., Host, K.M., Dittmer, D.P., and Damania, B. (2014). An important role for mitochondrial antiviral signaling protein in the Kaposi's sarcoma-associated herpesvirus life cycle. J. Virol. *88*, 5778–5787.

Wu, J.J., Li, W., Shao, Y., Avey, D., Fu, B., Gillen, J., Hand, T., Ma, S., Liu, X., Miley, W., et al. (2015). Inhibition of cGAS DNA Sensing by a Herpesvirus Virion Protein. Cell Host Microbe *18*, 333–344.

Xu, H., Xiao, T., Chen, C.-H., Li, W., Meyer, C.A., Wu, Q., Wu, D., Cong, L., Zhang, F., Liu, J.S., et al. (2015). Sequence determinants of improved CRISPR sgRNA design. Genome Res. *25*, 1147–1157.

Yada, K., Do, E., Sakakibara, S., Ohsaki, E., Ito, E., Watanabe, S., and Ueda, K. (2006). KSHV RTA induces a transcriptional repressor, HEY1 that represses rta promoter. Biochem. Biophys. Res. Commun. *345*, 410–418.

Yoo, S.M., Zhou, F.C., Ye, F.C., Pan, H.Y., and Gao, S.J. (2005). Early and sustained expression of latent and host modulating genes in coordinated transcriptional program of KSHV productive primary infection of human primary endothelial cells. Virology *343*, 47–64.

Zhang, G., Chan, B., Samarina, N., Abere, B., Weidner-Glunde, M., Buch, A., Pich, A., Brinkmann, M.M., and Schulz, T.F. (2016). Cytoplasmic isoforms of Kaposi sarcoma herpesvirus LANA recruit and antagonize the innate immune DNA sensor cGAS. Proc. Natl. Acad. Sci. USA *113*, E1034–E1043.

Zhang, Y., Dittmer, D.P., Mieczkowski, P.A., Host, K.M., Fusco, W.G., Duncan, J.A., and Damania, B. (2018). RIG-I detects Kaposi's sarcoma-associated herpesvirus transcripts in a RNA polymerase III-independent manner. MBio *9*, e00823-18.

Zhao, Y., Ye, X., Dunker, W., Song, Y., and Karijolich, J. (2018). RIG-I like receptor sensing of host RNAs facilitates the cell-intrinsic immune response to KSHV infection. Nat. Commun. *9*, 4841.

Zhu, F.X., Cusano, T., and Yuan, Y. (1999). Identification of the immediate-early transcripts of Kaposi's sarcoma-associated herpesvirus. J. Virol. *73*, 5556–5567.

Zhu, F.X., Chong, J.M., Wu, L., and Yuan, Y. (2005). Virion proteins of Kaposi's sarcoma-associated herpesvirus. J. Virol. *79*, 800–811.

Ziehr, B., Vincent, H.A., and Moorman, N.J. (2016). Human Cytomegalovirus pTRS1 and pIRS1 Antagonize Protein Kinase R To Facilitate Virus Replication. J. Virol. *90*, 3839–3848.

STAR★METHODS

KEY RESOURCES TABLE

REAGENT or RESOURCE	SOURCE	IDENTIFIER
Antibodies		
Mouse-anti-LANA	Leica Biosystems	NCL-L-HHV8-LNA
Mouse-anti-ORF57	Santa Cruz Biotechnology	Cat# sc-135746; RRID: AB_2011972
Mouse-anti-ORF45	Santa Cruz Biotechnology	Cat# sc-53883; RRID: AB_783766
Mouse-anti-K-bZIP	Santa Cruz Biotechnology	Cat# sc-69797; RRID: AB_1124280
Mouse-anti-K8.1 A/B	Santa Cruz Biotechnology	Cat# sc-65446; RRID: AB_831825
Rabbit-anti-RTA	David Lukac, Rutgers University	N/A
Mouse-anti-K5	Klaus Früh (Vaccine and Gene Therapy Institute, Oregon Health & Science University)	N/A
Rabbit-anti-ORF37(SOX) antibody	Britt Glaunsinger, University of California, Berkeley	N/A
Polyclonal sheep-anti-STX7 antibody	R and D Systems	Cat# AF5478; RRID: AB_2239977
Polyclonal goat-anti-VAMP8 antibody	R and D Systems	Cat# AF5354; RRID: AB_2304196
Rabbit-anti-PKN2 antibody	Thermo Fisher Scientific	Cat# A302-444A-T; RRID: AB_2780539
Rabbit-anti-AJUBA antibody	Thermo Fisher Scientific	Cat# A304-867A-T; RRID: AB_2621062
Rabbit-anti-PKR antibody	Cell Signaling Technology	Cat# 12297; RRID: AB_2665515
Rabbit-anti-PKR antibody	Abcam	Cat# ab184257
Polyclonal rabbit anti-eIF2alpha /EIF2S1 antibody	Thermo Fisher Scientific	Cat# A300-721A-M; RRID: AB_2779417
Polyclonal Rabbit-Phospho-eIF2alpha (Ser51) antibody	Cell Signaling Technology	Cat# 9721; RRID: AB_330951
Mouse Anti-beta-Actin Monoclonal antibody	Sigma-Aldrich	Cat# A5316; RRID: AB_476743
Monoclonal Rabbit Anti-alpha-Tubulin Monoclonal antibody	Cell Signaling Technology	Cat# 2125; RRID: AB_2619646
Rabbit Anti-GAPDH Monoclonal antibody	Cell Signaling Technology	Cat# 2118; RRID: AB_561053
Mouse anti MHC class I (W6/32) antibody	Hybridoma/Lehner lab stock	N/A
Mouse anti- human ICAM-1 antibody	Lehner lab stock	N/A
APC anti-human CD146 monoclonal antibody	BioLegend	Cat# 361015; RRID: AB_2564359
APC anti-human CD155 (PVR) antibody	BioLegend	Cat# 337618; RRID: AB_2565815
APC anti-human CD112 (Nectin-2) monoclonal antibody	BioLegend	Cat# 337412; RRID: AB_2565730
Human ULBP-2 monoclonal antibody	R and D Systems	Cat# MAB1298; RRID: AB_2214692
Purified anti-human EphA2 antibody	BioLegend	Cat# 356801; RRID: AB_2561804
anti-human EphA2 antibody	Santa Cruz Biotechnology	Cat# 398832
APC anti-human CD340 (erbB2/HER-2) monoclonal antibody	BioLegend	Cat# 324407; RRID: AB_756123
APC anti-human CD273 (B7-DC, PD-L2) monoclonal antibody	BioLegend	Cat# 345507; RRID: AB_2162177
CD97 monoclonal antibody	Thermo Fisher Scientific	Cat# 17-6979-42; RRID: AB_10548515
CD104 (Integrin beta 4) monoclonal antibody	Thermo Fisher Scientific	Cat# 14-1049-80; RRID: AB_1210461
Mouse Anti-Human Axl monoclonal antibody	R and D Systems	Cat# MAB154; RRID: AB_2062558

(*Continued on next page*)

Continued

REAGENT or RESOURCE	SOURCE	IDENTIFIER
APC anti-human CD262 (TRAIL-R2) antibody	BioLegend	Cat# 307407; RRID: AB_2204813
Goat anti-Rabbit IgG Secondary Antibody, Alexa Fluor 647	Thermo Fisher Scientific	Cat# A-21245; RRID: AB_2535813
Rabbit anti-Mouse IgG Secondary Antibody, Alexa Fluor 647	Thermo Fisher Scientific	Cat# A-21239; RRID: AB_2535808
Purified Mouse IgG1, κ Isotype control antibody	BioLegend	Cat# 401401; RRID: AB_2801452
Mouse IgG2a isotype control antibody	Immunotools	Cat #21335021
Rabbit IgG Isotype Control Monoclonal antibody	Cell Signaling Technology	Cat# 3900; RRID: AB_1550038
HA tag monoclonal antibody [16B12] (DyLight® 650)	Abcam	ab117515; RRID: AB_10999718
Anti-mouse IgG, HRP-linked antibody	Cell Signaling Technology	Cat# 7076; RRID: AB_330924
Anti-rabbit IgG, HRP-linked antibody	Cell Signaling Technology	Cat# 7074; RRID: AB_2099233
Anti-FLAG-tag M2 antibody	Sigma-Aldrich	Cat# F3165; RRID: AB_259529
Bacterial and Virus Strains		
NEB® 5-alpha Competent *E. coli*	NEB	Cat# C2987
Stbl4 ElectroMax electrocompetent cells	Thermo Fisher Scientific	Cat#11635018
Chemicals, Peptides, and Recombinant Proteins		
TMT10plex Isobaric Label Reagent	Thermo Fisher Scientific	Cat#90110
Trypsin, Mass Spectrometry Grade	Thermo Fisher Scientific	Cat#90057
Phosphonoacetic acid (PAA)	Sigma-Aldrich	Cat# 284270
Polyinosinic–polycytidylic acid sodium salt (Poly I:C)	Sigma-Aldrich	Cat# P0913
Doxycycline hyclate	Sigma-Aldrich	Cat#D9891
Hygromycin B	Invitrogen	Cat#10687010
Puromycin	Cayman Chemicals	Cat#13884
PhosSTOP phosphatase inhibitor	Roche	Cat#4906845001
Critical Commercial Assays		
PreOmics-IST NHS Sample preparation kit	PreOmics	Cat#P.O.00030
TransIT-293 transfection reagent	Mirus Bio	Cat# MIR 2704
SYBR Green PCR Master Mix	Thermo Fisher Scientific	Cat#4309155
RNeasy FFPE kit	QIAGEN	Cat# 73504
QIAamp DNA FFPE Tissue Kit	QIAGEN	Cat# 56404
Deposited Data		
Raw proteomics data	This paper	PRIDE http://proteomecentral.proteomexchange.org/PXD021387 and 10.6019/PXD021387
Sequencing data from CRISPR/Cas9 screens	This paper	Sequence Read Archive (https://www.ncbi.nlm.nih.gov/sra)/ SRP280153
Experimental Models: Cell Lines		
HEK293T	Lehner Lab stock	N/A
HuAR2T-tert	(May et al., 2010)	N/A
HuAR2T.rKSHV.219	(Vieira and O'Hearn, 2004; Alkharsah et al., 2011).	N/A
Oligonucleotides		
qPCR K8.1_for 5′-AAAGCGTCCAGGCCACCACAGA-3′	(Gramolelli et al., 2020)	N/A

(*Continued on next page*)

Continued

REAGENT or RESOURCE	SOURCE	IDENTIFIER
qPCR K8.1_rev 5′-GGCAGAAAATGGCACACGGTTAC-3′	(Gramolelli et al., 2020)	N/A
qPCR PKR_for 5′-TACGTGTGAGTCCCAAAGCA-3′	Merck	N/A
qPCR PKR_rev 5′-GGTCAAATCTGGGTGCCAAA-3′	Merck	N/A
qPCR BST2_for 5′-ACACTGTGATGGCCCTAATG-3′	Merck	N/A
qPCR BST2_rev 5′-CGTCCTGAAGCTTATGGTTTAATG-3′	Merck	N/A
qPCR LIMD1_for 5′-TGGGGAACCTCTACCATGAC-3′	Merck	N/A
qPCR LIMD1_rev 5′-CACAAAACACTTTGCCGTTG-3′	Merck	N/A
qPCR 18S_for 5′-GTAACCCGTTGAACCCCATT-3′	(Hartenian et al., 2020)	N/A
qPCR 18S_rev 5′-CCATCCAATCGGTAGTAGCG-3′	(Hartenian et al., 2020)	N/A
Recombinant DNA		
pLVX RTA	This paper	N/A but based on the RTA sequence pLenti4-Flag-K-RTA (Ko et al., 2012)
pLVX BFP	This paper	N/A
pCDNA3.1 ORF57_FLAG	(Vogt et al., 2015)	N/A
pHRSIN-pSFFV-ORF37(SOX)-IRES-mCherry	This paper	N/A but based on ORF37 sequence pCDEF-strep-SOX vector (provided by Prof. Britt Glaunsinger, University of California, Berkeley)
pHRSIN-pSFFV- PKR-4xHA-PGK-Hygro	This paper	N/A but based on the PKR sequence Addgene cat #20030
pHRSIN GFP-HA	Lehner Lab	N/A
pHRSIN GFP	Lehner Lab	N/A
pHRSIN K5 GFP	(Thomas et al., 2008a)	N/A
pHRSIN-pSFFV-Cas9-pPGK-Blasticidin	(Burr et al., 2017)	N/A
pKLV-U6gRNA(BsmBI)-PGKhygro2ABFP	Lehner Lab	N/A but based on Addgene #50946
pKLV-U6gRNA KSHV CRISPR Library	This paper	N/A
pKLV-U6gRNA(BbsI)-PGKhygro2ABFP	This paper	Based on Addgene #50946
pKLV K5 sgRNA#1 (GTGGACGACATCCAGCTCTC)	This paper	N/A
pKLV K5 sgRNA#2 (GGCGTAGTCGCCTTAACCTG)	This paper	N/A
pKLV K5 sgRNA#3 (ATACGCGGCAAATAACACCC)	This paper	N/A
pKLV K8.1 sgRNA#1 (CATGGCACGCCACCAGACAA)	This paper	N/A
pKLV K8.1 sgRNA#2 (GGCATCGGTCAGTTCTGTGG)	This paper	N/A
pKLV ORF34 sgRNA#1 (GTCGGCCCGACAAAAAGAGG)	This paper	N/A
pKLV ORF34 sgRNA#2 (CCTCGGGCAGGGTTTCGGGG)	This paper	N/A
pKLV ORF45 sgRNA#1 (GTATGGGCCCGTCTGGCCAG)	This paper	N/A

(*Continued on next page*)

Continued

REAGENT or RESOURCE	SOURCE	IDENTIFIER
pKLV ORF45 sgRNA#2 (TGGGAATATGAACTTCACGG)	This paper	N/A
pKLV CTRL sgRNA#1 (GTGGTAGCCACCTGGTGCGC)	This paper	N/A but based on control sgRNA sequence from pooled sgRNA library (Addgene# 1000000067)
pKLV CTRL sgRNA#2 (GCCATCTAGGCCTGTGTTGC)	This paper	N/A but based on control sgRNA sequence from pooled sgRNA library (Addgene # 1000000067)
pKLV ORF57 sgRNA#1 (ATTATGAAGGGCATCCTAGA)	This paper	N/A
pKLV ORF57 sgRNA#2 (CGATTCGTCAAACTCAGAGG)	This paper	N/A
Software and Algorithms		
Prism v.5.02	GraphPad	https://www.graphpad.com/scientific-software/prism/
FlowJo v10.5.2	FlowJo, LLC	https://flowjo.com
R version 3.4.4	R Core Team	https://www.r-project.org/
RStudio v1.0.44	R Studio	https://rstudio.com/
Bioconductor packages (Limma)	(Huber et al., 2015)	https://bioconductor.org/packages/release/bioc/html/limma.html
Mascot v2.3	Matrix Science	https://www.matrixscience.com/
Proteome Discoverer v2.2	Thermo Fisher Scientific	Cat# OPTON-30808
Morpheus	Broad Institute	https://software.broadinstitute.org/morpheus
Oligonucleotide web design tool	Genscript	https://genscript.com/tools/real-time-pcr-taqman-primer-design-tool
DAVID Bioinformatics Resources	DAVID	https://david.ncifcrf.gov/

RESOURCE AVAILABILITY

Lead Contact

Further information and requests for resources and reagents should be directed to and will be fulfilled by the lead contact, Ildar Gabaev (ig329@cam.ac.uk).

Materials availability

All unique reagents generated in this study are available upon request.

Data and code availability

The mass spectrometry proteomics data generated during this study have been deposited to the ProteomeXchange Consortium (http://proteomecentral.proteomexchange.org) via the PRIDE partner repository (Perez-Riverol et al., 2019) with the

dataset identifier PXD021387 and 10.6019/PXD021387. Sequencing data from KSHV CRISPR/Cas9 screens presented in this study have been deposited at the Sequence Read Archive (SRA)/SRP280153.

EXPERIMENTAL MODEL AND SUBJECT DETAILS

HEK293T cells (obtained from ATCC) were grown in Iscove's Modified Dulbecco's Medium (Sigma) supplemented with 10% fetal calf serum (GIBCO). An endothelial cell line, HuAR2T-tert, conditionally immortalized with doxycycline-dependent human telomerase reverse transcriptase (hTERT) and simian virus 40 (SV40) T antigen (May et al., 2010) was a kind gift of Dr. Dagmar Wirth (HZI Braunschweig, Germany). HuAR2T.rKSHV.219 cells is a HuAR2T-tert cell line that harbors latent KHSV genomes (Alkharsah et al., 2011; Vieira and O'Hearn, 2004). Both cell lines were propagated in EGM-2 Endothelial Medium Bullet Kit (Cat# CC-3156 and CC-4176, Lonza), with 5% FCS and doxycycline (1ug/ml). All media were supplemented with penicillin (100 units/ml), streptomycin sulfate (100ug/ml), and 1xGlutamax (GIBCO).

METHOD DETAILS

Plasmids construction

For pLVX FLAG_RTA vector construction, RTA sequence was amplified from pLenti4-Flag-K-RTA (Ko et al., 2012) (a kind gift of Dr. Su-Fang Lin, National Institute of Cancer Research, Taiwan) and cloned into pLVX TRE3G backbone (Clontech). For pHRSIN-pSFFV-PKR_4xHA vector construction, the PKR cDNA was amplified from pSB819-PKR-hum vector (Addgene #20030, kindly deposited by Harmit Malik) and cloned into pHRSIN-pSFFV-GFP-PGK-Hygro vector. To generate pKLV-U6gRNA(BbsI)-PGKhygro2ABFP vector, the puromycin resistance cassette in pKLV-U6gRNA(BbsI)-PGKpuro2ABFP (Addgene #50946, kindly deposited by K. Yusa) was replaced with hygromycin. For pHRSIN-pSFFV-ORF37(SOX)-IRES-mCherry vector construction, ORF37 sequence was amplified from pCDEF-strep-SOX vector (a kind gift of Britt Glaunsinger, University of California, Berkeley) and cloned into pHRSIN-pSFFV-MCS-IRES-mCherry vector. All the cloning procedures were performed using NEBuilder HiFi DNA Assembly master Mix (Cat# E2611L).

Lentivirus production and transduction

293T cells were transfected with a lentivirus expression vector and the packaging vectors pCMVΔR8.91 and pMD.G using TransIT-293 transfection reagent (Mirus) according to manufacturer's recommendations. Supernatants were harvested 48 h post transfection and passed through 0.45 um filter units. Typically, cells were transduced at an MOI < 1 in 6 well plates by centrifugation at 800 g for 1h. The following drug concentrations were used for selection of transduced cells: puromycin (4 ug/ml), blasticidin (3 ug/mL) or hygromycin (150 ug/ml).

KSHV reactivation for proteomic analysis

HuAR2T.rKSHV.219 cells (Experiment 1) or HuAR2T.rKSHV.219 Cas9 cells (Experiments 2 and 3) were transduced with LV RTA or LV BFP control, harvested 65 h post-transduction (Experiments 1 and 2) or at 36, 48 and 60h upon transduction (experiment 3), fixed with 2% formaldehyde in PBS for 30 min, washed three times with PBS and sorted on RFP+ or BFP+ markers. In the proteomics experiment 3, where indicated, 500 μM phosphonoacetic acid (PAA) was added 1 hour upon transduction. The resulting samples contained 2,5x10*5 (Experiment 1, Figure 1D), 4x10*5 (Experiment 2, Figure 3A) and 3,5 x10*5 (Experiment 3, Figure 6A) cells each. Sorted cells were lysed for protein isolation followed by trypsin digest and TMT reporter labeling. The resulting peptides were mixed, fractionated and analyzed by mass spectrometry.

Digestion and TMT Labeling

Samples were prepared using an IST-NHS digestion kit (Preomics) according to the manufacturer's instructions with minor modification. Briefly, samples were lysed by heating at 95 degrees in lysis buffer for 45 minutes. This is longer than the 10min indicated by the protocol to effect decrosslinking (Ly et al., 2017). Nucleic acids were sheared by sonication in a Bioruptor sonicator (30 s on/off, for 10 minutes) (Diagenode). Samples were quantified by BCA assay against a BSA standard curve dissolved in lysis buffer. 25μg of each sample was reduced, alkylated and digested according to the manufacturer's protocol. TMT labeling was not conducted prior to the clean-up step as suggested in the protocol. Instead, samples were cleaned up according to the protocol, dried under vacuum, resuspended in 21uL 100mM Triethylammonium bicarbonate buffer to which were added 0.2mg of TMT label dissolved in 9μL anhydrous acetonitrile (ACN). The reaction was allowed to proceed for 2h at room temperature before pooling of samples. Sample pools were partially dried under vacuum to remove excess ACN and subsequently diluted to ~1mL with 0.1% Trifluoracetic acid (TFA). Additional formic acid was added to bring the pH < 2. Samples were subjected to SPE clean-up using 50mg tC-18 cartridges (Waters). Cartridges were wetted with 1mL methanol, followed by 1mL ACN and equilibrated with 3mL 0.1% TFA. They were then loaded onto cartridges, washed with 1mL 0.1% TFA and eluted sequentially with 250uL 40% ACN, 60% ACN and 80% ACN. Eluates were dried under vacuum.

High pH Reverse Phase Fractionation

Samples were subjected to HpHRP fractionation on an Ultimate 3000 UHPLC system (Thermo Scientific) equipped with a 2.1 mm × 15 cm, 1.7μ Kinetex Evo C18 column (Phenomenex, UK). Solvent A was 3% ACN, Solvent B was 100% ACN, solvent C was 200 mM ammonium formate (pH 10). Throughout the analysis, solvent C was kept at a constant 10%. The flow rate was 400 μL/min and UV was monitored at 280 nm. Samples were loaded in 90% A for 10 min before a gradient elution of 0%–10% B over 10 min (curve 3), 10%–34% B over 21 min (curve 5), 34%–50% B over 5 mins (curve 5) followed by a 10 min wash with 90% B. 15 s (100 μL) fractions were collected throughout the run. Peptide containing fractions were recombined into 24 fractions (i.e., fractions 1, 25, 49, 73, 97 combined) and dried in a vacuum centrifuge. Fractions were stored at −80°C prior to analysis.

Mass spectrometry

Data were acquired on an Orbitrap Fusion mass spectrometer (Thermo Scientific) coupled to an Ultimate 3000 RSLC nano UHPLC (Thermo Scientific). HpRP fractions were resuspended in 20 μl 5% DMSO 0.5% TFA and 10μL injected. Fractions were loaded at 10 μl/min for 5 min on to an Acclaim PepMap C18 cartridge trap column (300 um × 5 mm, 5 um particle size) in 0.1% TFA. After loading a linear gradient of 3%–32% solvent B over 3 hr was used for sample separation over a column of the same stationary phase (75 μm × 50 cm, 2 μm particle size) before washing at 90% B and re-equilibration. Solvents were A: 0.1% FA and B:ACN/0.1% FA. An

SPS/MS3 acquisition was used for all samples and was run as follows. MS1: Quadrupole isolation, 120’000 resolution, 5e5 AGC target, 50 ms maximum injection time, ions injected for all parallelisable time. MS2: Quadrupole isolation at an isolation width of m/z 0.7, CID fragmentation (NCE 35) with the ion trap scanning out in rapid mode from m/z 120, 8e3 AGC target, 70 ms maximum injection time, ions accumulated for all parallelisable time. In synchronous precursor selection mode the top 10 MS2 ions were selected for HCD fragmentation (65NCE) and scanned out in the orbitrap at 50’000 resolution with an AGC target of 2e4 and a maximum accumulation time of 120 ms, ions were not accumulated for all parallelisable time. The entire MS/MS/MS cycle had a target time of 3 s. Dynamic exclusion was set to ± 10 ppm for 90 s, MS2 fragmentation was trigged on precursor ions 5e3 counts and above.

CRISPR Cas9-mediated disruption of KSHV ORFs

Sense and antisense oligonucleotides (Merck) were phosphorylated using T4 PNK (NEB) at 37°C for 30 min, annealed at 95°C for 5 min and cooled to room temperature. The resulting annealed fragments were treated with BpiI endonuclease (Thermo Fischer Scientific) and cloned into BpiI-treated pKLV-U6gRNA(BbsI)-PGKhygro2ABFP vector using T7 ligase (NEB). The resulting reactions were transformed in chemically competent *E.coli* DH10B cells and selected on agar plates with ampicillin. Plasmid DNA was isolated using QIAprep Spin Miniprep Kit (QIAGEN) and validated by sequence analysis. For CRISPR Cas9-mediated gene disruption, HuAR2T.rKSHV.219 cells were first transduced with pHRSIN-pSFFV-Cas9-pPGK-Blasticidin lentivirus vector (Burr et al., 2017) followed by blasticidin selection. The cells were transduced with lentiviruses expressing sgRNAs targeting KSHV ORFs: K5 (sgRNA#1 5′-GTGGACGACATCCAGCTCTC-3′ and sgRNA#2 5′-GGCGTAGTCGCCTTAACCTG-3′), ORF34 (sgRNA#1 5′-GTCGGCCCGA-CAAAAAGAGG-3′ and sgRNA#2 5′-CCTCGGGCAGGGTTTCGGGG-3′), ORF45 (sgRNA#1 5′-GTATGGGCCCGTCTGGCCAG-3′ and sgRNA#2 5′-TGGGAATATGAACTTCACGG-3′), K8.1 (sgRNA#1 5′-CATGGCACGCCACCAGACAA-3′ and sgRNA#2 5′-GGCATCGGTCAGTTCTGTGG-3′) or control sgRNAs (sgRNA#1 5′-GTGGTAGCCACCTGGTGCGC-3′ and sgRNA#2 5′-GCCATC-TAGGCCTGTGTTGC-3′). The cells were selected with hygromycin B for seven days and analyzed by flow cytometry or immunoblot. For analysis of CRISPR-mediated rescue of ICAM-1 depletion by K5, HuAR2T.rKSHV.219 Cas9 cells were transduced with lentiviruses encoding K5-specific sgRNA#1, sgRNA#2 (sequences are provided above), sgRNA#3 (5′- ATACGCGGCAAATAACACCC-3′) or $\beta-2$-microglobulin (b2M)-targeting sgRNA (5′-GGCCGAGATGTCTCGCTCCG-3′). To generate CRISPR K5 virus for proteomic analysis HuAR2T.rKSHV.219 Cas9 cells were transduced with lentiviruses encoding K5-specific sgRNA#1 and sgRNA#2. To generate CRISPR ORF57 virus HuAR2T.rKSHV.219 Cas9 cells were transduced with lentiviruses encoding ORF57-specific sgRNA#1 (5′-ATTATGAAGGGCATCCTAGA-3′) and sgRNA#2 (5′-CGATTCGTCAAACTCAGAGG-3′).

Validation of cellular targets of KSHV K5 protein

For validation of K5 targets by flow cytometry HuAR2T cells were transduced with pHRSIN K5 GFP or control pHRSIN GFP lentiviruses, harvested 72 hours later and stained with isotype control antibody or antibody specific for Erbb2, EphA2, TRAIL-R2, PD-L2, CD146 (Biolegend). For validation of targets of K5 by immunoblot, HuAR2T cells were transduced as indicated above and 72 hours upon transduction GFP-high cell population was selected by FACS, expanded for additional 7 days, lysed and analyzed with antibody specific for EphA2 (Santa Cruz Biotechnology), STX7, VAMP8 (R and D Systems) and K5 (kind gift of Klaus Frueh, Vaccine and Gene Therapy Institute, Oregon Health & Science University).

Flow cytometry analysis

For validation of KSHV-mediated downregulation of cellular proteins by flow cytometry, lytic KSHV cycle was induced by transduction of HuAR2T.rKSHV.219 cells with LV RTA and treatment with 1.5 mM sodium butyrate (‘RTA reactivation mix’). For cell surface staining, cells were washed with blocking buffer (4% FCS in PBS) and incubated with the primary antibody specific for CD155, Nectin-2 (Biolegend), ULBP-2, hAxl (R and D Systems), CD97, CD104 (Thermo Fisher Scientific), MHC class I and ICAM-1. The following antibody were used as isotype controls: mouse IgG1 kappa (Biolegend), mouse IgG2a (Immunotools) and rabbit IgG (Cell signaling) isotype control antibody. After 1 hour incubation the cells were washed three times with blocking buffer and subsequently incubated with anti-rabbit or anti-mouse Alexa Fluor 647-conjugated secondary antibody for another hour. All procedures were performed at 4°C. For intracellular staining, cells were fixed with 2% formaldehyde for 20 min, washed twice with PBS, permeabilised with 0.2% Triton x-100 in PBS for 15 min and nonspecific binding sites were blocked with intracellular blocking buffer (1% BSA, 0.1% gelatine, PBS) for 30 min. The antibodies were diluted in blocking buffer following the recommendations of the manufacturer. Cells were either incubated with anti-HA tag DyLight 650 antibody (Abcam) or with antibody specific for PKR (Cell signaling) for one hour, washed three times with PBS and subsequently incubated with a dye-conjugated secondary antibody for additional hour. Measurements were performed on LSR II Fortessa (Becton Dickinson) flow cytometer and analyzed using FlowJo (TreeStar) software. Ten thousand events were counted for each sample. Sorts were performed on an Influx cell sorter (Becton Dickinson).

Immunoblot analysis

For validation of the hits identified by proteomics analysis, HuaR2T.rKSHV.219 cells were transduced with BFP control or RTA lentiviruses, harvested at 48h or 60h upon transduction, washed with PBS twice and treated with fixing solution (2% formaldehyde in PBS) for 20 minutes. Where indicated, 500 μM phosphonoacetic acid (PAA) was added to the cells 1 hour upon transduction. BFP+ or RFP+ cell populations were sorted by FACS, lysed in lysis buffer (4% SDS, 200 mM NaCl, 0.01M NaP04 pH 6.0) and treated with

ultrasound (10 cycles, 15 s each). Lysates of fixed cells were additionally subjected to protein de-cross-linking procedure by incubating at 95 degrees for 30 min. Total protein amount in the samples was measured by BCA assay (Thermo Fischer Scientific) and the amount of cell lysate equivalent to 20 ug of protein per lane was resolved on SDS-page gel. The immunoblot analysis was performed with antibody specific for FLAG-tag (Sigma-Aldrich), KSHV LANA (Leica Biosystems), RTA (kind gift of David Lukac, Rutgers University), ORF57, ORF45, K-bZIP, K8.1 A/B (Santa Cruz Biotechnology), PKR (Cell signaling), PKN2, Ajuba (Thermo Fischer Scientific) dissolved in PBS with 0.1% Tween-20. For induction of eIF2α phosphorylation, 5x10*5 HuaR2T.rKSHV.219 Cas9 cells were mock-transduced or transduced with LV RTA and treated with 1.5 mM sodium butyrate for 22h and 34h followed by transfection with 2 ug of poly I:C (Sigma-Aldrich) using Lipofectamine 2000 (Invitrogen). The cells were harvested 2 hours later, lysed in the presence of PhosSTOP phosphatase inhibitor (Roche) and analyzed by immunoblot with antibody specific for PKR (Abcam), eIF2alpha (Thermo Fischer Scientific), Phospho-eIF2alpha (Cell Signaling). Membranes were washed three times in TBS with 0.1% Tween-20 and probed with anti-mouse or anti-rabbit HRP-linked antibody (Cell signaling). Antibodies specific for beta-actin (Sigma-Aldrich) and alpha-tubulin (Cell signaling) were used as loading controls. Signals were visualized by chemiluminescence using ECL and ECL Dura western blotting detection reagents and iBright imaging system (Thermo Fischer Scientific).

qRT-PCR analysis

HuAR2T.rKSHV.219 cells were transduced with control BFP (latent) and RTA (lytic) lentiviruses, harvested 60h upon transduction, fixed with 2% formaldehyde and sorted on BFP+ and RFP+. Total mRNA from the cells was isolated using RNeasy FFPE kit (QIAGEN) according to manufacturer specifications. 1 ug of purified RNA was taken for cDNA synthesis using SuperScript III Reverse Transcriptase (Thermo Fisher Scientific) in 50 ul reaction. Host gene-specific primers were designed using Genscript web design tool (https://www.genscript.com/tools/real-time-pcr-taqman-primer-design-tool), sequences of the primers specific for lytic K8.1 gene were reported previously (Gramolelli et al., 2020). Transcript levels were determined by quantitative real-time reverse transcriptase PCR (qPCR) using SYBR green dye (Thermo Fisher Scientific) incorporation and 20 ng of cDNA per reaction. The comparative threshold cycle method was used to determine the change in gene expression between the samples, using 18S for normalization.

KSHV CRISPR library design and cloning

List containing the coding sequences of canonical KSHV ORFs (GenBank: GQ994935.1), alternative KSHV ORFs (Arias et al., 2014) and miRNA precursors (Gottwein, 2012) was used as a source file for generation of 19179 sgRNA sequences and calculation their efficiency score (Xu et al., 2015). The following sgRNAs were excluded from the analysis: (1) containing BsmBI restriction sites, stretches of four or more thymines or five guanines; (2) those that fully match or align with up to three mismatches with the coding regions of the human genome; (3) those that align with up to four mismatches with KSHV genome. As more than four mismatches prevent induction of DSB by Cas9 (Cho et al., 2014; Hsu et al., 2013), the selected sgRNAs will not hit off- targets in the viral genome and are unlikely to target the host genome. Duplicated sgRNA sequences were merged and ranked on the aforementioned efficiency score. Typically, ten sgRNAs with the highest score were selected for each KSHV ORF. Due to their small size, several ORFs were targeted by less than ten sgRNAs, in such cases all the sgRNAs for these ORFs were included in the library. Of note, sgRNAs specific for KSHV ORF50 (RTA) and vIL6.3 were excluded from the library, because RTA is essential for reactivation and vIL6.3-specific sgRNAs did not pass the quality filter described above. The resulting library contained 1281 KSHV-specific and 50 non-targeting sgRNAs from the Activity-Optimized Human CRISPR Pooled Library (Addgene #1000000067) (Wang et al., 2015) (sequences are presented in Table S2). sgRNA sequences flanked by BsmBI restriction sites and unique adapters (Doench et al., 2016) were synthesized as a pool of single-stranded oligonucleotides by Custom Array (Bothell, USA). The pool of KSHV-specific and control sgRNAs was amplified with subpool-specific primers, cloned into BsmBI-treated pKLV-U6gRNA(BsmBI)-PGKhygro2ABFP vector using T7 DNA Ligase (NEB), electroporated into Stbl4 Electromax competent cells and selected on ten 15 cm dishes with ampicillin. Plasmid DNA containing sgRNA library was extracted from ~68000 clones (50-fold coverage) and sequenced on Illumina MiniSeq platform.

Genetic screens with KSHV CRISPR sgRNA library

To obtain ~1000-fold coverage of each sgRNA in the target cell population, ~5x10*6 HuAR2T.rKSHV.219 Cas9 cells were transduced with lentivirus-encoded KSHV sgRNA library at an MOI of 0.3, selected on hygromycin B and expanded for 10 days. 1x10*7 cells stably expressing KSHV sgRNA library were treated with the 'RTA reactivation mix' for lytic cycle induction, harvested 65h later, stained with APC-conjugated CD155- or Nectin-2-specific antibody and fixed with 2% formaldehyde in PBS. The RFP+(lytic) and APC-high (rescued CD155 and Nectin-2 downregulation) cell population was selected by FACS and used for genomic DNA extraction using QIAamp DNA FFPE Tissue Kit (QIAGEN). Individual integrated sgRNAs were sequenced using the Illumina MiniSeq platform. Statistical analysis of the enriched sgRNAs in the sorted versus unsorted cell populations was performed using RSA algorithm (König et al., 2007).

QUANTIFICATION AND STATISTICAL ANALYSIS

Proteomic data processing and analysis

Spectra were searched by Mascot within Proteome Discoverer 2.1 in two rounds of searching. The first search was against the UniProt Human reference proteome (26/09/17), the KSHV proteome, a database of non-canonical KSHV sequences (Arias et al., 2014)

and compendium of common contaminants (GPM). The second search took all unmatched spectra from the first search and searched against the human trEMBL database (Uniprot, 26/0917). The following search parameters were used. MS1 Tol: 10 ppm, MS2 Tol: 0.6 Da, Fixed mods: Ist-alkylation (+113.084064 Da) (C) and TMT (N-term, K), Var mods: Oxidation (M), Enzyme: Trypsin (/P). MS3 spectra were used for reporter ion-based quantitation with a most confident centroid tolerance of 20 ppm. PSM FDR was calculated using Mascot percolator and was controlled at 0.01% for 'high' confidence PSMs and 0.05% for 'medium' confidence PSMs. Normalization was automated and based on total s/n in each channel. Protein/peptide abundance was calculated and output in terms of 'scaled' values, where the total s/n across all reporter channels is calculated and a normalized contribution of each channel is output. Proteins/peptides satisfying at least a 'medium' FDR confidence were taken forward to statistical analysis in R. This consisted of a moderated t test (Limma) with Benjamini-Hochberg correction for multiple hypotheses to provide a q value for each comparison (Schwämmle et al., 2013). Hierarchical cluster analysis was performed with Morpheus analysis software (https://software.broadinstitute.org/morpheus) using Euclidean distance or one minus Pearson correlation metrics for distance measurements.

Gene Ontology analysis

Enrichment of Gene Ontology (GO) Molecular Function (GOTERM_MF_Direct) and Biological Process (GOTERM_BP_Direct) terms from proteins whose expression was dysregulated by lytic KSHV was determined using the Database for Annotation, Visualization and Integrated Discovery (DAVID) (accessed on 23/8/2019 at https://david.ncifcrf.gov/) with default settings (Huang et al., 2009a, 2009b). Proteins significantly downregulated or upregulated by lytic KSHV infection (FDR-adjusted $p < 0.05$) in two proteomic experiments (Figures 1D and 3A), quantitated with at least two unique peptides were used as a 'gene set' and analyzed against a background of all proteins quantitated.

Cell Reports, Volume *33*

Supplemental Information

Quantitative Proteomics Analysis of Lytic KSHV Infection in Human Endothelial Cells Reveals Targets of Viral Immune Modulation

Ildar Gabaev, James C. Williamson, Thomas W.M. Crozier, Thomas F. Schulz, and Paul J. Lehner

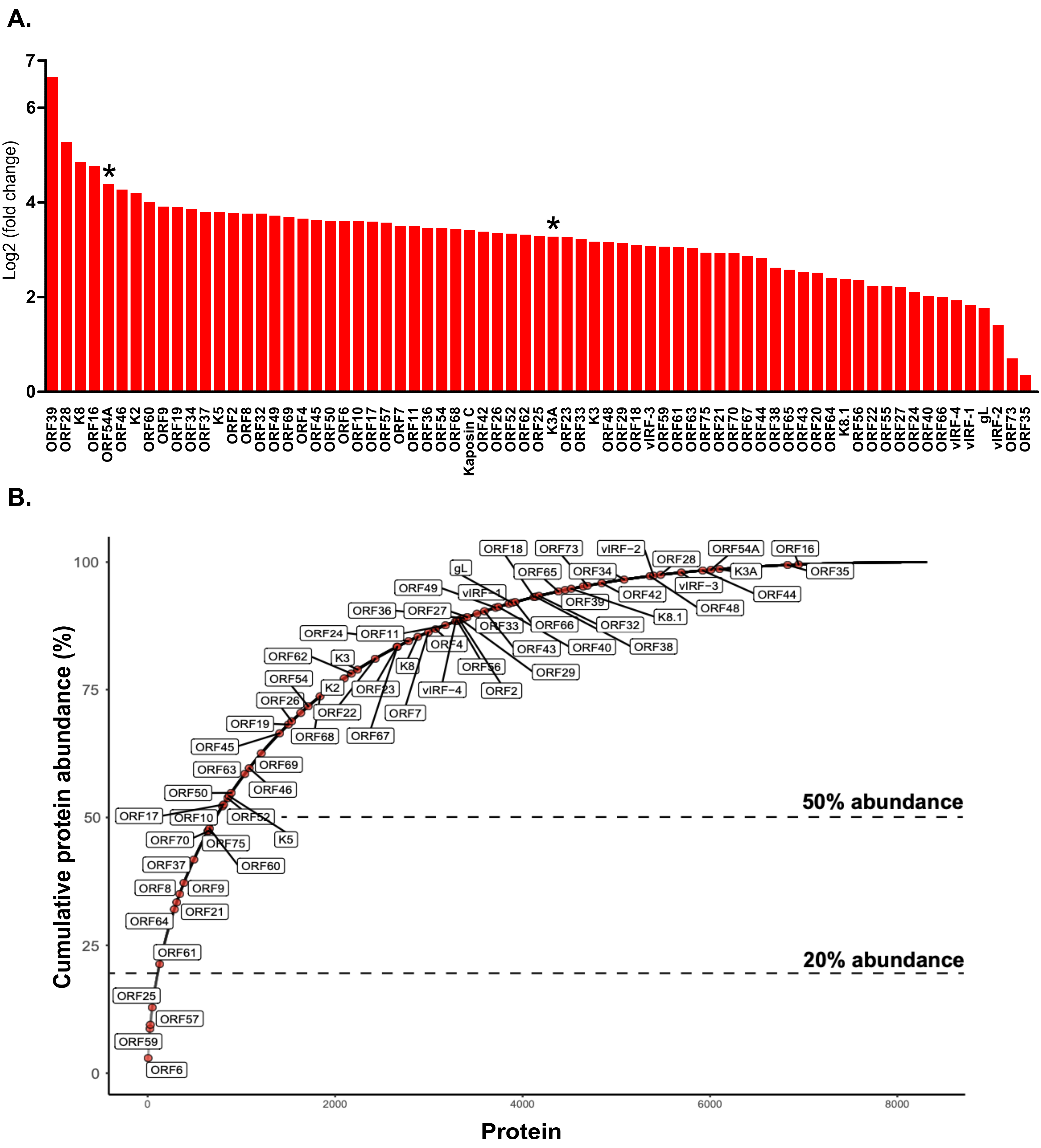

Figure S1. KSHV proteins identified in quantitative proteomics experiments. Related to Figure 1.
(A) The histogram shows fold change in abundance of the KSHV proteins, quantified in one of two proteomics experiments with two peptides and $q < 0.05$. An asterisk denotes alternative viral translational products reported in ribosome profiling study (Arias et al., 2014).
(B) A plot of cumulative sum of protein abundance versus proteins ranked by abundance (most abundant proteins are in the left bottom corner). Viral proteins are highlighted in red and annotated.

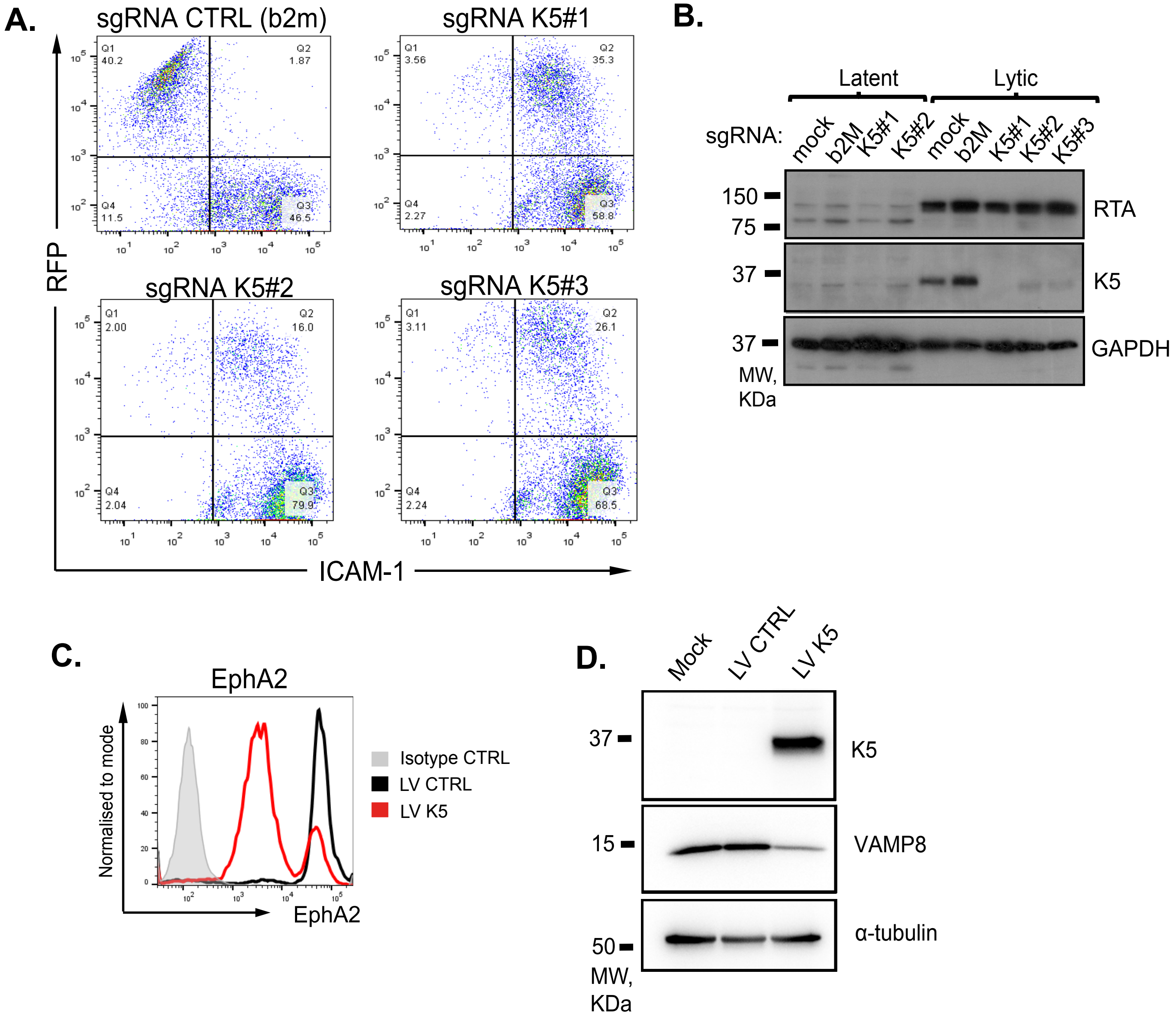

Figure S2. CRISPR Cas9-mediated depletion of KSHV K5 unravels novel K5 targets. Related to Figures 2 and 3.
(A) K5-specific sgRNAs rescue lytic KSHV-mediated downregulation of ICAM-1. HuAR2T.rKSHV.219 Cas9 cells were transduced and treated as indicated above, stained with ICAM-1-specific antibody and analysed by flow cytometry.
(B) KSHV K5 is depleted with CRISPR Cas9 and K5-specific sgRNAs. HuAR2T.rKSHV.219 Cas9 cells were transduced with lentivirus expressing individual KSHV K5-specific sgRNA or control (b2M) sgRNA, left untreated (latent) or treated with 'reactivation mix' to induce lytic KSHV cycle. Cells were harvested 65 h later and analysed by immunoblot with antibody specific for KSHV RTA and K5 proteins. GAPDH was used as the loading control.
(C) KSHV K5 downregulates EphA2 receptor. HuAR2T cells were transduced with control GFP (LV CTRL, black line) or K5 GFP (LV K5, red line) lentiviruses, harvested 72 hpt, stained with isotype control antibody (grey shading) or antibody specific for EphA2 and analysed by flow cytometry.
(D) KSHV K5 downregulates VAMP8. HuAR2T cells were transduced with control GFP (LV CTRL) or K5 GFP (LV K5) lentiviruses, harvested 72 h later, sorted on GFP+ and analysed by immunoblot with K5- and VAMP8-specific antibody. α- tubulin was used as the loading control.

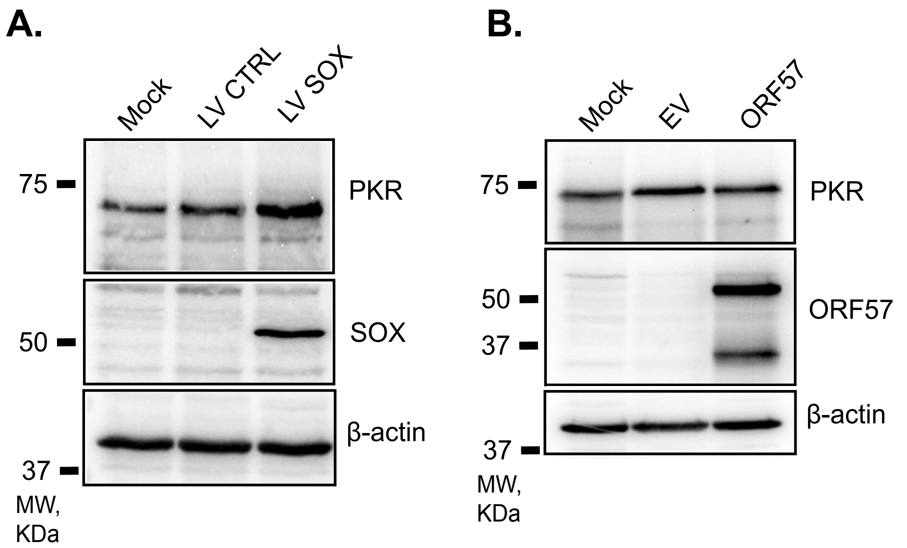

Figure S3. PKR is not downregulated by KSHV ORF37 (SOX) or ORF57. Related to Figure 4.
(A) HuAR2T cells were transduced with control lentivirus (LV CTRL) or lentivirus expressing KSHV ORF37 (LV SOX), harvested 60 h later and analysed with antibody specific for PKR and KSHV SOX protein. β-actin was used as the loading control.
(B) HEK293 cells were transfected with expression vectors encoding ORF57 or empty vector (EV), harvested 60 h later and analysed by immunoblot with antibody specific for PKR and KSHV ORF57.

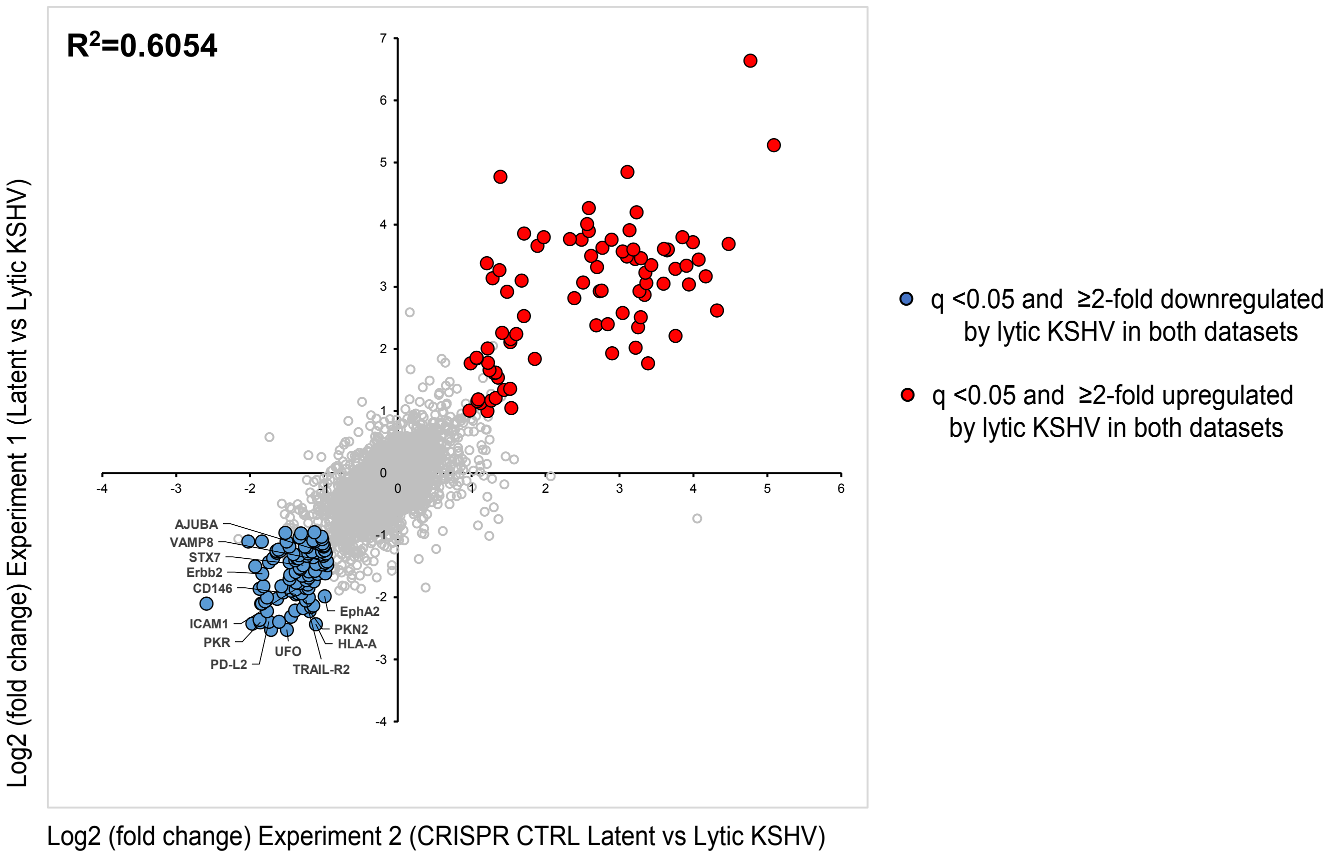

Figure S4. Scatterplot showing pairwise comparison from two independent proteomics experiments. Related to Figures 1 and 3. Each point represents a single protein, plotted by its log2 (fold change in abundance) in the experiment 1 (X axis, as shown in Figure 1D) vs experiment 2 (Y axis, as shown in Figure 3A). Annotated proteins were selected for validation by flow cytometry and/or immunoblot analysis (Figure 3F-H, Figure S3A,B, Figure 4B,D, Figure 5E,I,J).

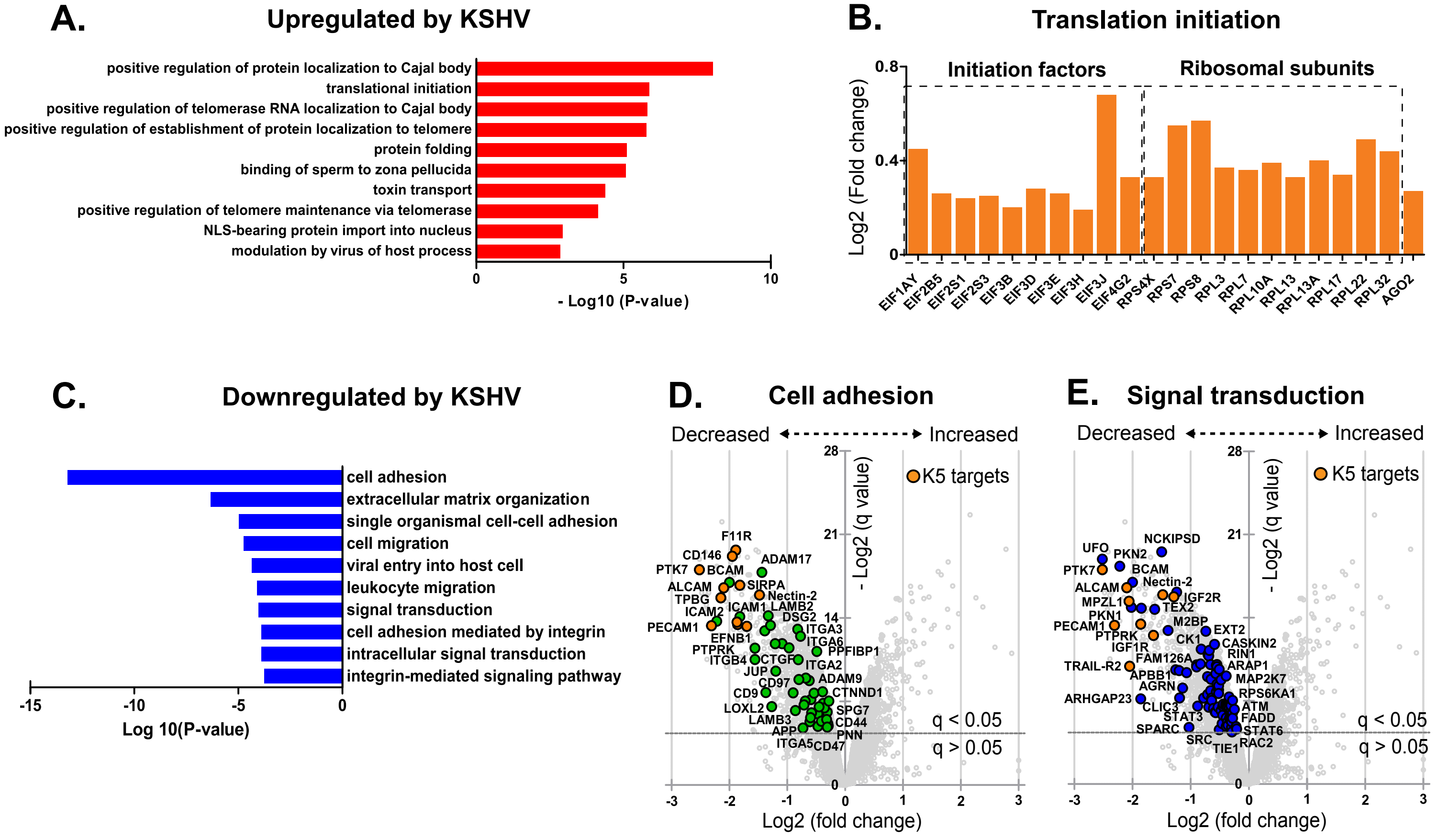

Figure S5. DAVID GO term analysis of cellular proteins from the category 'Biological process' dysregulated by lytic KSHV infection. Related to Figure 5. See also Table S4.

(A) Gene Ontology (GO) functional annotation terms enriched amongst upregulated proteins with q < 0.05 in cells with lytic vs latent KSHV infection from two proteomic experiments (Figures 1E and 3D). Ten most enriched GO terms (ranked by p-value) in the category 'Biological process' amongst upregulated proteins are shown.

(B) The histogram shows fold change in abundance of the proteins from the GO term 'Translation initiation'. Factors of translation initiation and ribosomal subunits are highlighted with dashed line squares.

(C) Ten most enriched GO terms (ranked by p-value) in the category 'Biological process' amongst downregulated proteins are shown.

(D, E) Downregulated proteins from the GO terms 'Cell adhesion' and 'Signal transduction' are highlighted, respectively as green and blue points on the scatterplots that display pairwise comparison between latent and lytic KSHV infection. Each point represents a single protein, plotted by its log2 (fold change in abundance) vs the statistical significance of that change. Orange points represent targets of KSHV K5 protein.

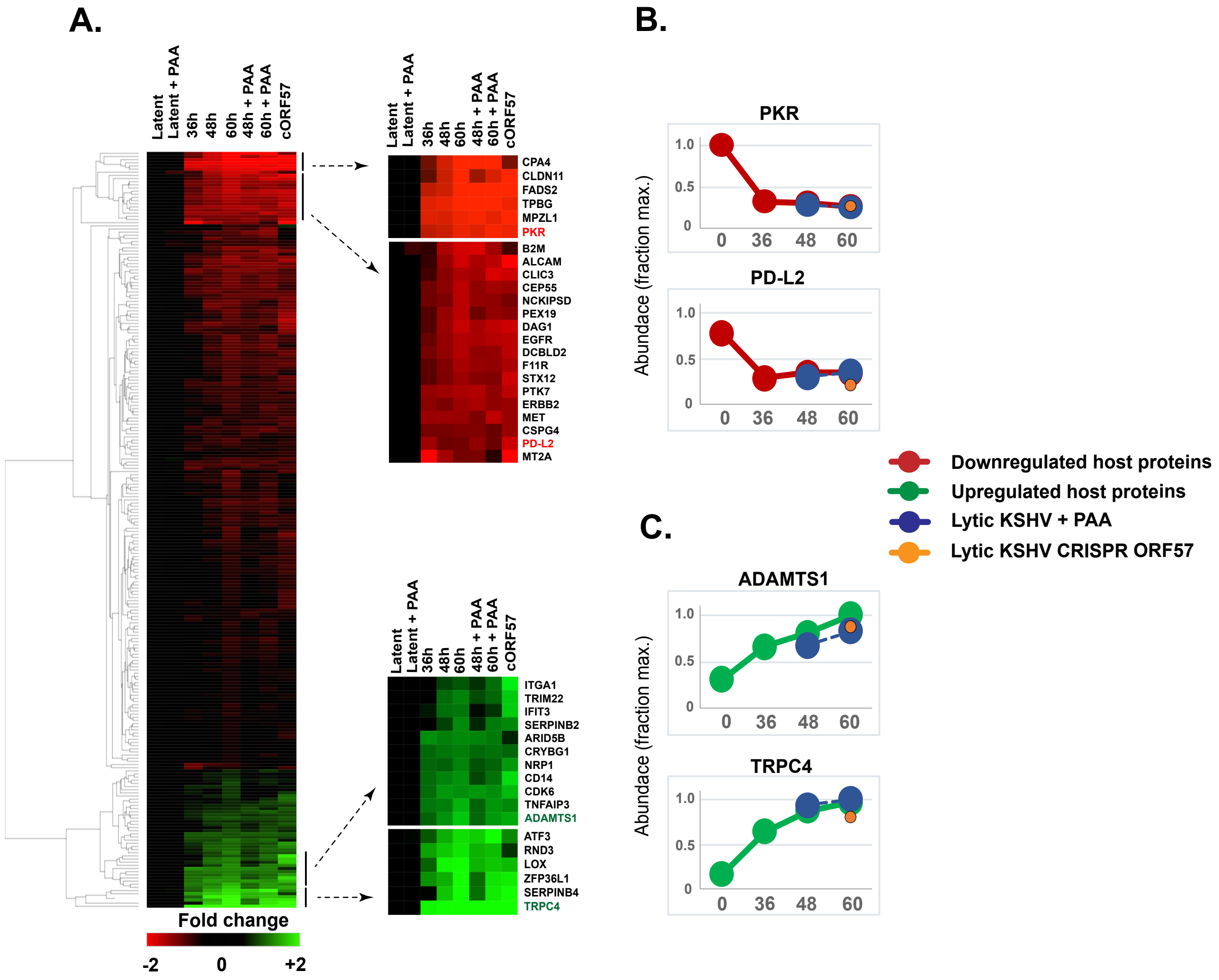

Figure S6. Kinetic profiling of cellular proteins in lytic KSHV infection. Related to Figure 6.
(A) Hierarchical cluster analysis of host cell proteins dysregulated by lytic KSHV infection. Heatmap diagram (left side) shows temporal kinetic (0,36,48,60 hours upon transduction), PAA-sensitivity and ORF57-dependency profiles for cellular proteins dysregulated by lytic KSHV. Rows and columns represent, respectively, individual cellular proteins and experimental samples. Cellular proteins 1.5-fold dysregulated by lytic KSHV infection in the proteomics experiment 3 (Figure 6A) and 1.5-fold dysregulated with $q < 0.05$ in the proteomics experiment 1 (Figure 1D) and experiment 2 (Figure 3A) were taken for cluster analysis. Enlarged segments of the heatmap (right side) show top two clusters of host proteins down- or upregulated by lytic KSHV infection.
(B, C) Kinetic profiles of host cell proteins dysregulated by lytic KSHV from the clusters shown in the Figure S6A. Protein abundance is calculated as a fraction of maximum TMT reporter ion intensity.

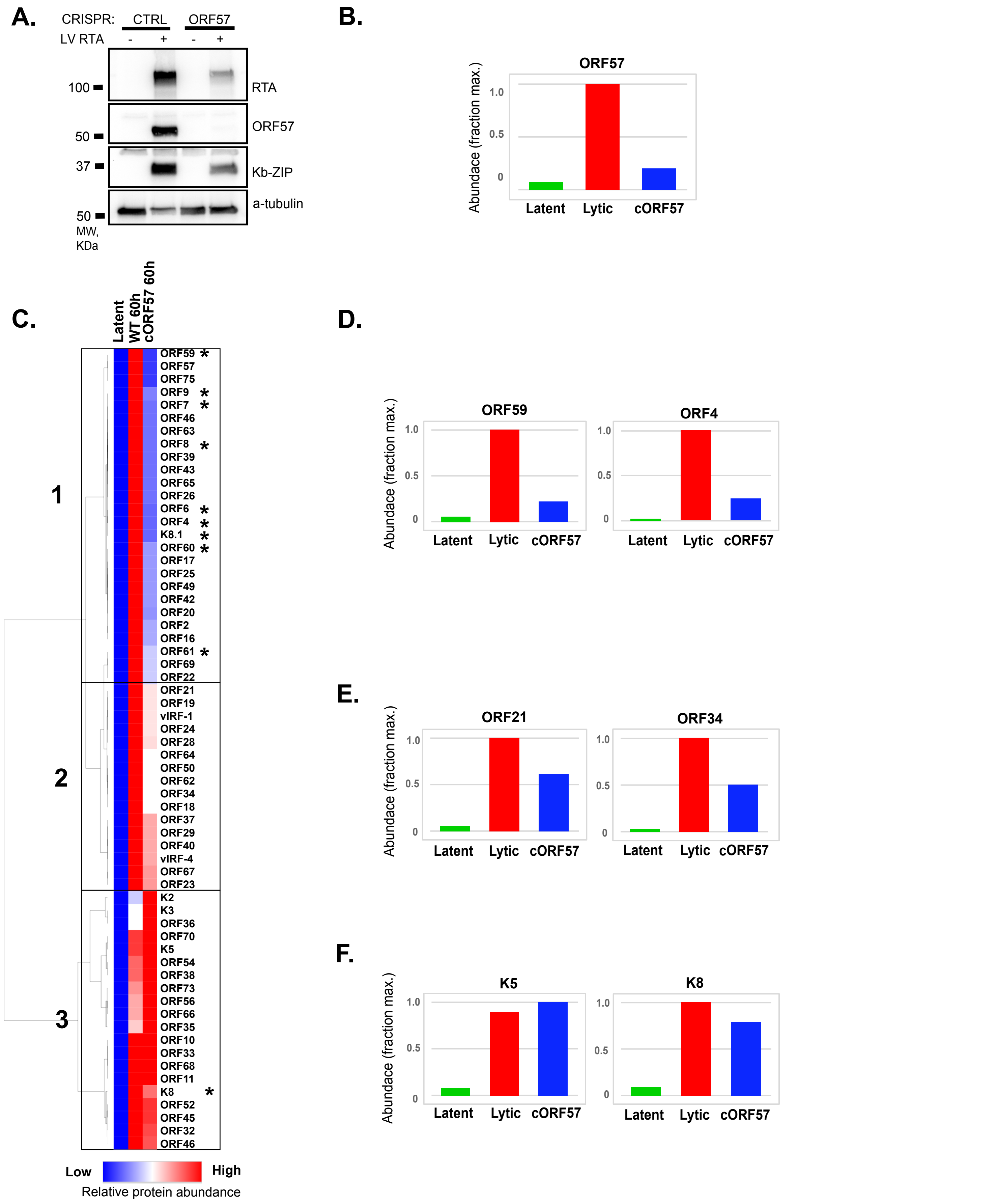

Figure S7. ORF57-dependent expression of viral ORFs. Related to Figure 6. (A) Immunoblot analysis of HuAR2T.rKSHV.219 Cas9 cells stably expressing control or ORF57-specific sgRNAs, transduced with LV RTA, harvested 60h later and probed with antibody against RTA, ORF57, Kb-ZIP proteins. (B) Relative abundance of ORF57 in the cells with KSHV latent (green), lytic control (blue) and lytic CRISPR ORF57 (red) infections. Protein abundance is calculated as a fraction of maximum TMT reporter ion intensity. (C) Hierarchical cluster analysis of all viral proteins quantified. Asterisks denote viral ORFs previously reported to be dependent on ORF57. Rows and columns represent, respectively, individual viral ORFs and experimental samples. Black squares show groups of viral proteins identified by hierarchical cluster analysis. (D-F) Relative abundances of the indicated viral proteins in the cells with KSHV latent (green), lytic control (blue) and lytic CRISPR ORF57 (red) infections.
